# Synthesis of Paclitaxel Derivatives for Remote Loading into Liposomes and Improved Therapeutic Effect

**DOI:** 10.3390/molecules27227967

**Published:** 2022-11-17

**Authors:** Xiangwei Xu, Yanhua Liu, Shanshan Shao, Jinbo Li, Zhaochu Xu, Yueling Yin, Linxiang Zhao, Yongjun Wang, Dan Liu

**Affiliations:** 1 Key Laboratory of Structure-Based Drugs Design & Discovery of Ministry of Education, Shenyang Pharmaceutical University, Shenyang 110016, China; 2 Department of Pharmaceutics, School of Pharmacy, Ningxia Medical University, Yinchuan 750004, China; 3 Wuya College of Innovation, Shenyang Pharmaceutical University, Shenyang 110016, China

**Keywords:** palictaxel derivatives, boronic acid, liposome, anti-tumor, drug delivery

## Abstract

A series of novel paclitaxel derivatives modified by boronic acid according to the characteristics of the interaction between RB(OH)_2_ and different strapping agents of intraliposomal aqueous phase were designed and synthesized, which were then used to develop remote poorly water-soluble drugs loading into liposomes. Meanwhile, we screened nineteen paclitaxel boronic acid derivatives for their cytotoxic activities against three cancer cell lines (A549, HCT-116 and 4T1) and one normal cell line (LO2), and performed liposome formulation screening of active compounds. Among all the compounds, the liposome of **4d**, with excellent drug-encapsulated efficiency (>95% for drug-to-lipid ratio of 0.1 *w*/*w*), was the most stable. Furthermore, the liposomes of compound **4d** (8 mg/kg, 4 times) and higher dose of compound **4d** (24 mg/kg, 4 times) showed better therapeutic effect than paclitaxel (8 mg/kg, 4 times) in the 4T1 tumor model in vivo, and the rates of tumor inhibition were 74.3%, 81.9% and 58.5%, respectively. This study provided a reasonable design strategy for the insoluble drugs to improve their drug loading into liposomes and anti-tumor effect in vivo.

## 1. Introduction

Paclitaxel, a natural product with effectiveness, is one of the most widely used clinical anticancer agents [1,2] and shows commendable antitumor activity against various cancers, especially breast cancer [3,4]. The mechanism of paclitaxel is to inhibit the proliferation of tumor cells by stabilizing the microtubules and inhibiting the G2 or M phases of the cell cycle [5]. It has been reported that Taxol^®^ is the first marketed preparation of paclitaxel, but histamine may be produced with the degradation of its cosolvent Cremophor EL(CrEL), which results in allergic reactions in patients [6,7]. Similar to Taxol^®^, and although other marketed paclitaxel formulations have shown potent antitumor activity against various cancers, there are still problems which limit their clinical applications, such as poor water solubility and low bioavailability, as well as drug resistance [8,9,10,11].

The advantage of a drug carrier is to improve the targeting ability, water solubility and PK properties of a drug [12,13,14,15,16,17]. Paclitaxel, as a non-ionizable drug, is difficult to stably encapsulate in a drug carrier. Fascicle chemical modification or derivatization of drugs is effective in developing remote poorly water-soluble drugs loading into liposomes. In our previous study, we found that weak acidic or basic derivatives of insoluble drugs can be actively encapsulated into liposomes by pH gradients [18,19,20]. In addition to pH gradients, there are many other remote loading gradients, such as copper ion gradients, which can complex with and encapsulate drugs into liposomes [21]. Boronic acid RB(OH)_2_ can both spontaneously complex with copper ion and undergo dynamic covalent reactions with diols, such as catechol, sugar and mannitol. Inspired by the characteristics of boronic acid RB(OH)_2_, we expected that the boronic acid derivatives would form precipitates by the complexation after entering the inner phase of liposomes, so as to achieve the active drug loading in liposomes.

Therefore, we designed and synthesized nineteen boric-acid-modified paclitaxel derivatives and evaluated their cytotoxic activities against three cancer cell lines (lung cancer cell line A549, colon cancer cell line HCT-116, breast cancer cell line 4T1) and one normal cell line (hepatocyte cell line LO2). In addition, the selected compounds **4a**, **4d**, **13a**, **17a** and **17c** were further screened by liposome prescription. As a result, compound **4d** was chosen as the preferred prodrug as it possessed better loading efficiencies (>95% for drug-to-lipid ratio of 0.1 *w*/*w*). Additionally, liposomes loaded with compound **4d** showed the best therapeutic effect in the 4T1 tumor model in vivo.

## 2. Results and Discussion

### 2.1. Design the Combination Strategy of Paclitaxel Derivatives

**Figure 1:**
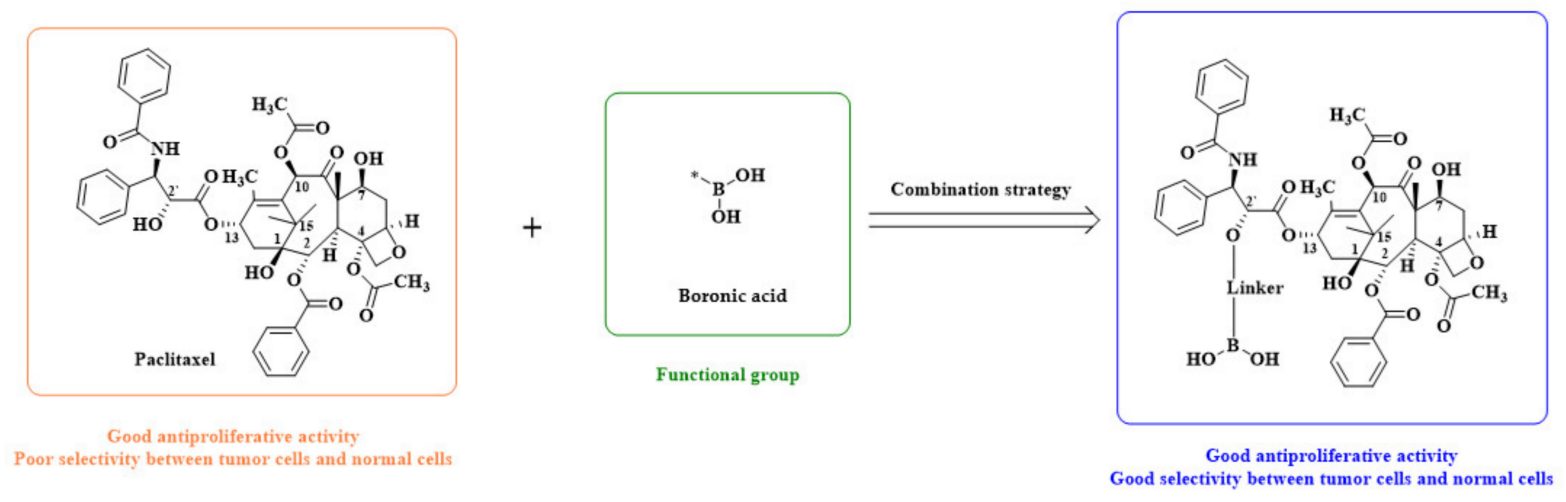
Combination strategy of designing paclitaxel derivatives by linking paclitaxel (as a parent drug) with a boronic acid (as the functional group) via ester bond or carbonate bond.

According to the structure-activity relationship of paclitaxel [22], the 2’-OH of paclitaxel is both an active site and a common structural modification site. Therefore, we introduced boronic acid into 2’-OH by using the combination strategy. There are three key parts in the designed derivatives (Figure 1): (i) Boronic acid group; (ii) Linker fragment; (iii) Paclitaxel drug. Boronic acid groups interact with the trapping agents of the intraliposomal aqueous phase to achieve the purpose that derivatives are encapsulated by liposomes. The Linker fragment is a breakable ester bond or carbonate bond.

### 2.2. Chemistry

**Scheme 1:**
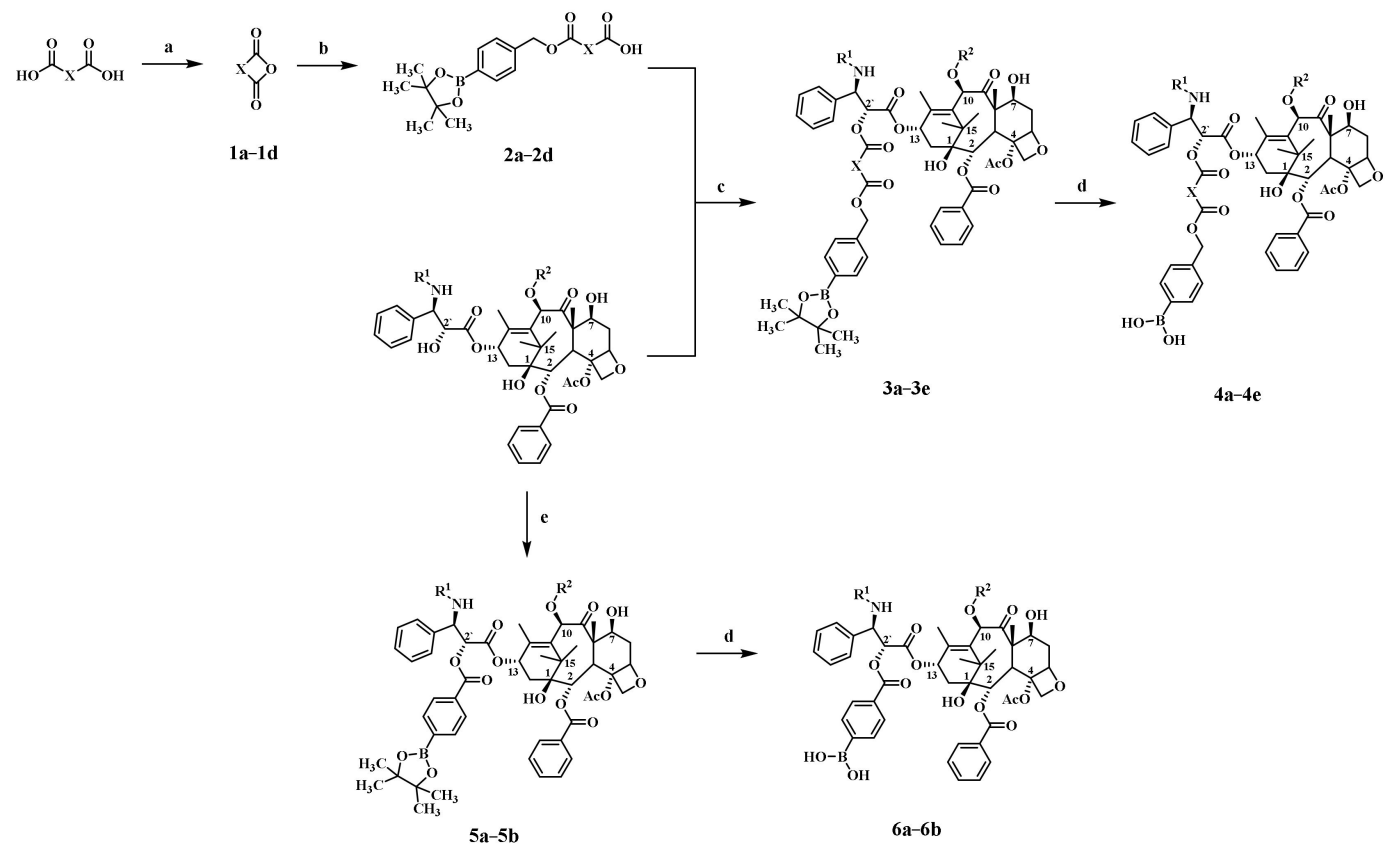
Reagents and conditions: a. Ac_2_O, r.t.; b. (4-(4,4,5,5-tetramethyl-1,3,2-dioxaborolan-2-yl)phenyl)methanol, DMAP, dry DCM, r.t.; c. EDCI, DMAP, dry DCM, r.t.; d. NaIO_4_, NH_4_OAc, H_2_O, acetone, r.t.; e. 4-(4,4,5,5-tetramethyl-1,3,2-dioxaborolan-2-yl)benzoic acid, EDCI, DMAP, dry DCM, r.t.

The synthetic routes of the target compounds are illustrated in Scheme 1. Anhydrides **1a–1d** were obtained via a dehydration reaction of alkyldicarboxylic acids, then reacted with 4-(hydroxymethyl)benzeneboronic acid pinacol ester to obtain intermediates **2a–2d**, and then esterified with paclitaxel or docetaxel to obtain key intermediates **3a–3e**. Finally, the target compounds **4a–4e** and **6a–6b** were obtained by oxidation hydrolysis.

**Scheme 2:**
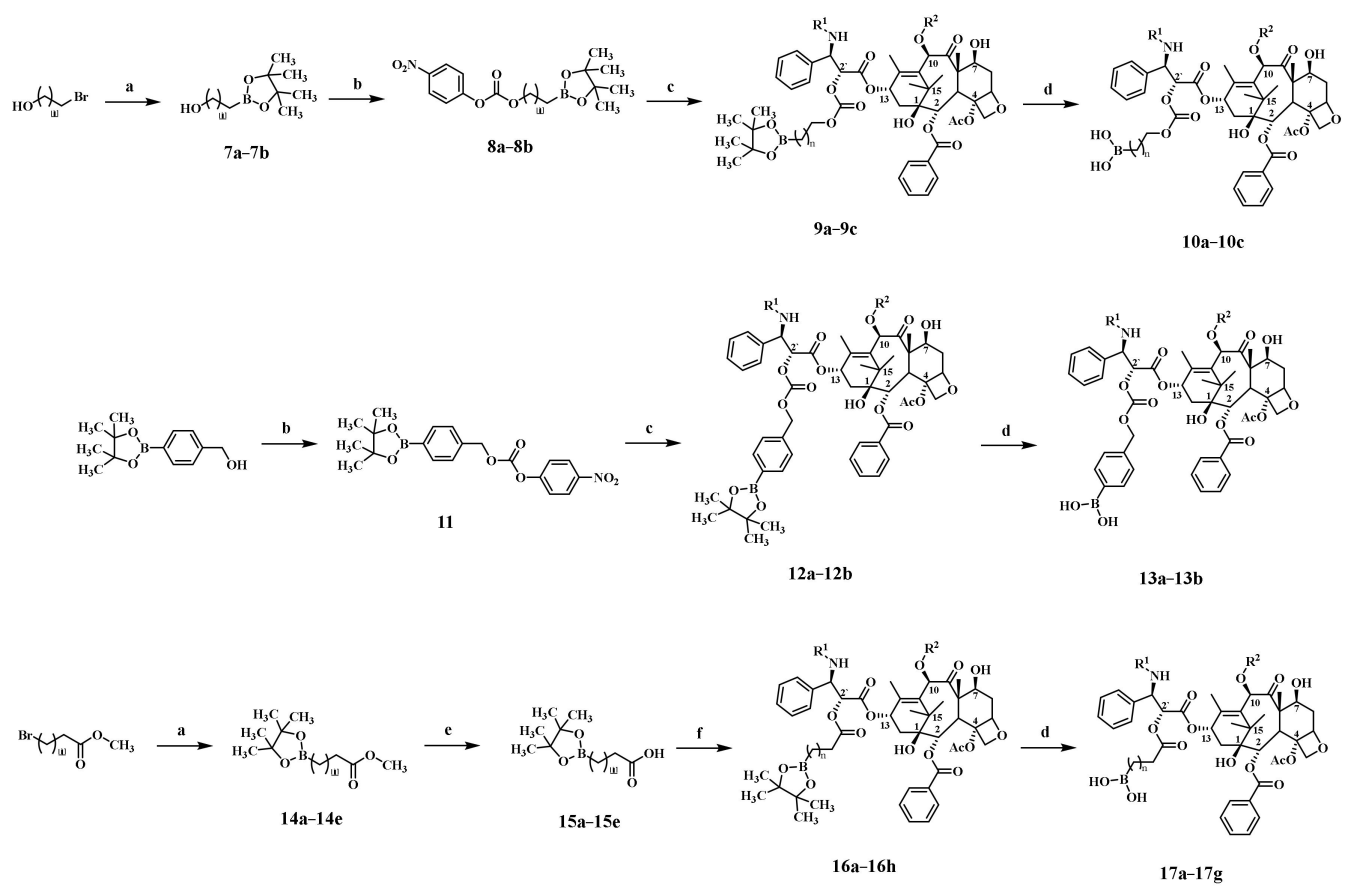
Reagents and conditions: a. bis(pinacolato)diboron, CuCl, PCy_3_, *t*-BuOK, dry THF, N_2_, 0 °C; b. 4-Nitrophenyl chloroformate, Py, dry DCM, −20 °C; c. paclitaxel or docetaxel, DMAP, dry DCM, r.t.; d. NaIO_4_, NH_4_OAc, H_2_O, acetone, r.t.; e. NaOH, MeOH, r.t.; f. paclitaxel or docetaxel, EDCI, DMAP, dry DCM, r.t.

The key intermediates **7a–7b** were obtained by Arbuzov reaction of bromoalkyl alcohols, and then reacted with 4-nitrophenyl chloroformate to give **8a–8b**, followed by esterification and oxidative hydrolysis to give the target compounds **10a–10c** (Scheme 2). The synthetic routes of the target compounds **13a–13b** and **17a–17g** were similar to those of the compounds **10a–10c**. The structures of target compounds, as listed in Table 1, were confirmed by ^1^H-NMR, ^13^C-NMR and HRMS.

### 2.3. In Vitro Cytotoxicity Studies

MTT assay was applied to verify the rationality of the molecular combination strategy. The cytotoxicity of the compounds was evaluated on lung cancer cell line A549, colon cancer cell line HCT-116, breast cancer cell line 4T1 and normal hepatocyte cell line LO2. Paclitaxel was used as a positive drug. The results showed that, compared with LO2, the tested compounds were more sensitive to HCT-116, A549 and 4T1, which suggested the cell selectivity of these compounds. The therapeutic index (TI) for LO2/HCT-116, LO2/A549 and LO2/4T1 of active compounds **4a**, **4d**, **13a**, **17a**, **17c** and paclitaxel are shown in Table 2.

According to the results of cytotoxicity, we roughly summarized the structure-activity relationship (SAR): (a) Compound **4d** with disulfide bonds showed better selectivity for tumor cells than compound **4a** without disulfide bonds. (b) The selectivity of aromatic boronic acid compound **13a** was much better than that of alkyl boronic acid compounds (**10a**–**10c**). (c) Compound **17c** (*n* = 3) showed the best selectivity, but when the carbon chain was further increased, the compounds **17d–17e** (*n* = 4, 5) showed no selectivity.

### 2.4. Design and Optimization of Compound **4d** Liposome Formulation

Since boronic acid RB(OH)_2_ is not only a weak acid, but also a structure that can complex with copper ions, ATP and saccharides, we investigated whether different inner phases could be employed to encapsulate compounds (**4a**, **4d**, **13a**, **17a**, **17c**) with good therapeutic index. Firstly, the encapsulated efficiency of calcium acetate gradients was systematically explored and it was found that it was not feasible with the amount of sediment precipitated after loading (Supplementary Materials: Table S1).

The reasons may be that log*D* (**4a**:2.466; **4d**: 2.413; **13a**: 2.540; **17a**: 2.309; **17c**: 2.559) and pKa (**4a**: 12.302; **4d**: 12.301; **13a**: 12.297; **17a**: 12.302; **17c**: 12.302) of the compounds (**4a**, **4d**, **13a**, **17a**, **17c**) were not suitable for calcium acetate gradients (−2.5 < log*D <* 2; pKa < 11) [23]. Vyxeos^®^ is a liposomal formulation of a fixed molar ratio (1:5) combination of the antineoplastic drugs daunorubicin (DN) and cytarabine (Cyt) [24]. Daunorubicin is subsequently actively encapsulated with high efficiency through complexation with intra-liposomal copper. The copper gluconate buffer forms a complex with daunorubicin at a ratio of 1:1 to 1:2 [25]. Referring to Vyxeos^®^, copper ion was selected as a complexed metal ion in the inner phase. In addition to compound **4d**, most derivatives (**4a**, **13a**, **17a**, **17c**) failed in loading into the liposomes, showing obvious sedimentation. Therefore, compound **4d** was selected to investigate the effect of the anions on the loading efficiency. Among them, only the neutral gluconate copper was a suitable inner phase to facilitate the encapsulation of compound **4d** (Table 3).

The reason for the stability of the neutral formulations was potentially due to the fact that PBA was effectively protonated and interacted with copper more tightly in the neutral condition compared with the acidic condition [26]. Thus, the neutral Cu-gluconate was selected as the inner phase. The concentration of copper ions was also investigated, showing that the higher the copper concentration, the higher the EE. Considering the safety of copper ions, 300 mM was chosen. For lipid composition, the content of Chol was 45% (molar ratio), which revealed higher EE. The EE of compound **4d** active loading liposome was more than 95% with copper ion gradient. Furthermore, we also evaluated the ATP and saccharides as remote gradients with the optimal formulation of copper ion gradients. The results of encapsulation efficiency are shown in Table 4.

The inner phase of ATP was not successful, which may be caused by the weak binding ability between PBA and ATP. The results of different saccharides gradients showed that saccharides could be selected as the inner phase of liposomes to load PBA-modified derivates, but the EE of compound **4d** was not high enough. Thus, the copper ion gradient was chosen for further experiments.

**Figure 2:**
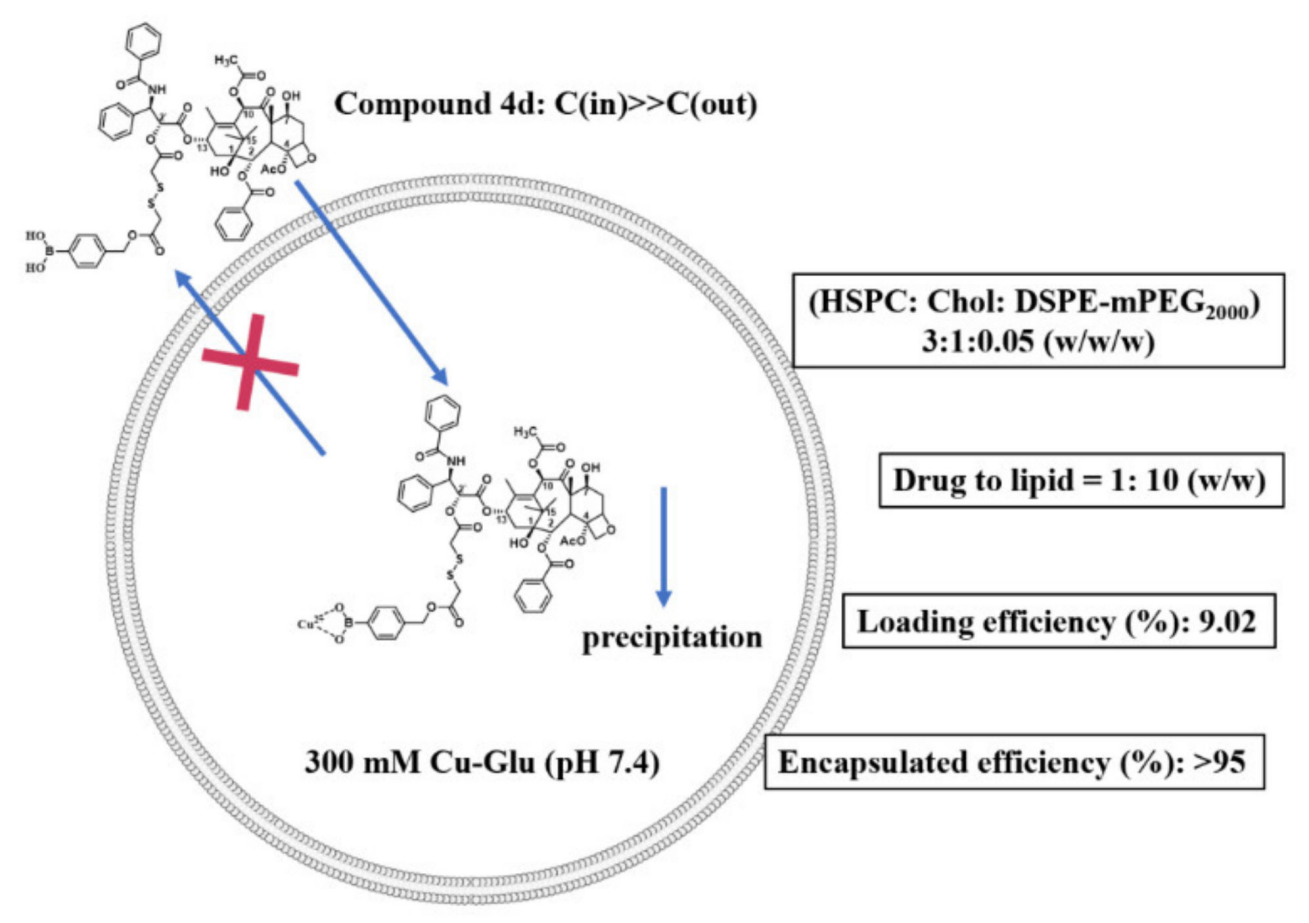
The structure of compound **4d** and its optimal formulations.

The optimal formulation was shown in Figure 2. The drug to lipid ratio was more than 1:10, drug loading efficiency was up to 9.02% and the prepared liposomes remained stable for more than two weeks at 4 °C.

**Figure 3:**
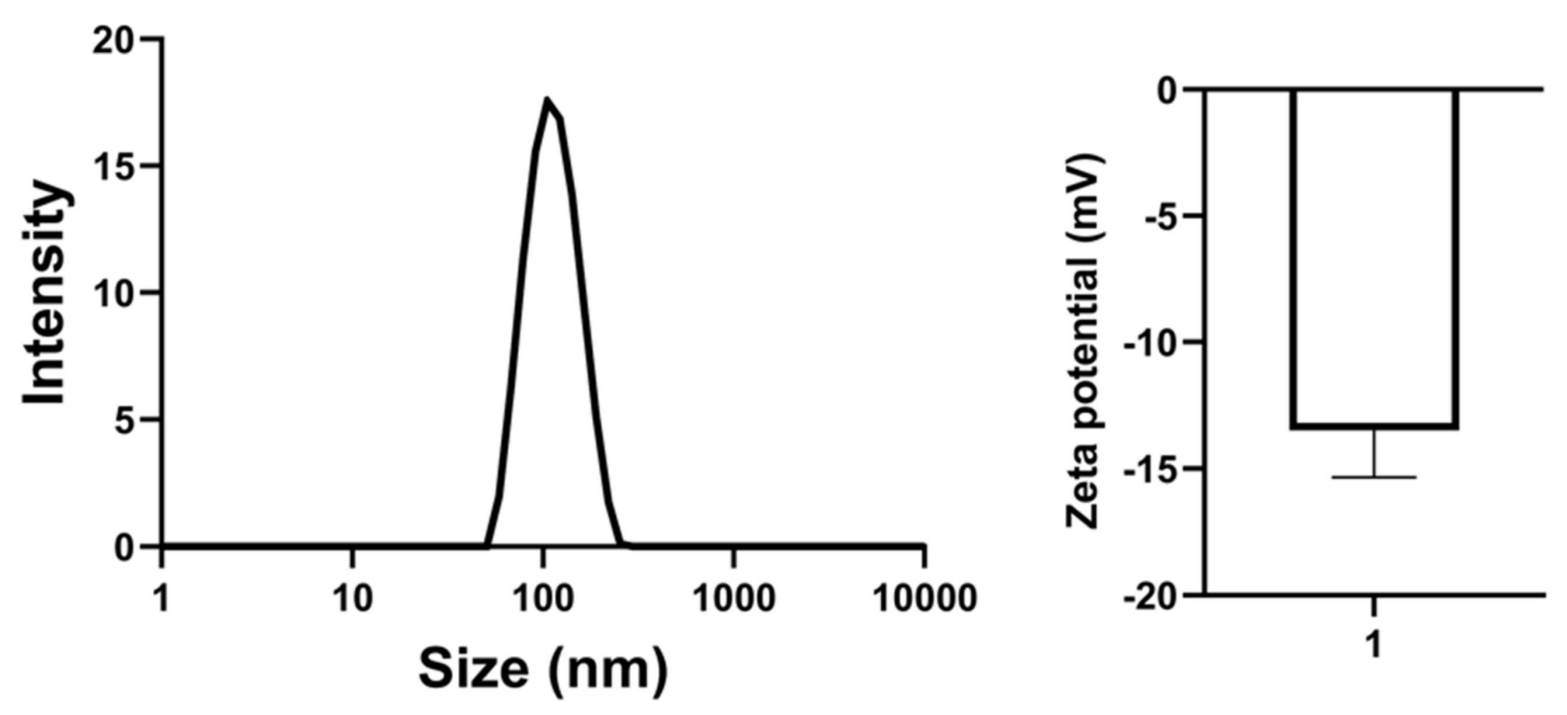
Particle size and zeta potential of compound 4d liposome.

**Figure 4:**
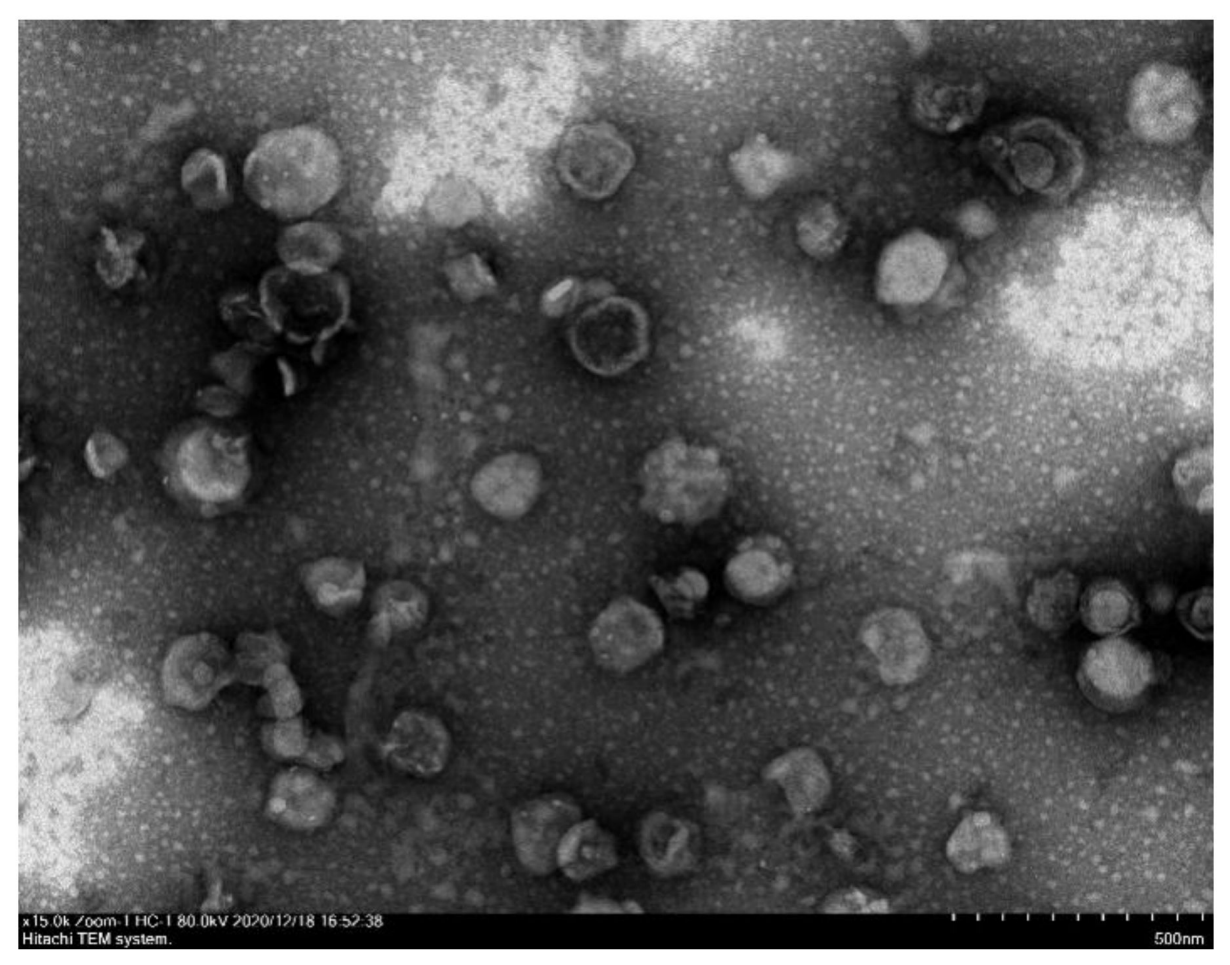
TEM image of compound 4d liposome.

The morphology of compound **4d** liposome is shown in Figure 3 and Figure 4, with spherical particles, a narrow size distribution at ~110 nm and zeta potential at ~−15 mV (Table S2).

### 2.5. In Vitro Release of PTX Derivative **4d** Liposomes

**Figure 5:**
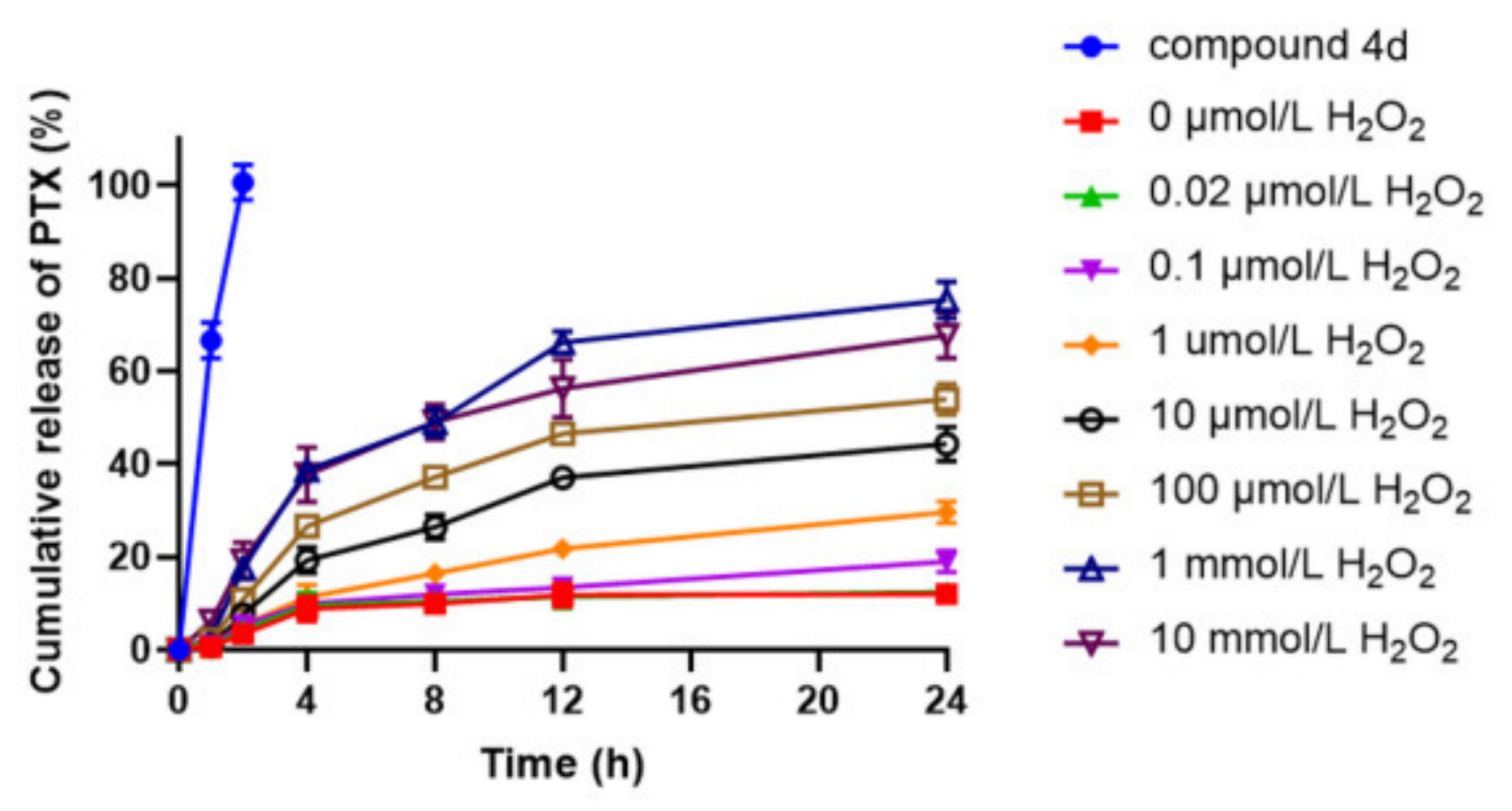
In vitro ROS-sensitive release studies of PTX.

In vitro release study of compound **4d** liposome (Figure 5) showed that compound **4d** was a ROS-sensitive paclitaxel derivative. When the content of H_2_O_2_ increased from 0 to 10 mmol/L, the paclitaxel released by compound **4d** significantly increased. Paclitaxel release at high H_2_O_2_ content (1 mM and 10 mM) for 24 h (75.3% and 67.7%) was much better than that at low H_2_O_2_ content (0.1 μM and 1 μM, 19.1% and 29.6%). The results showed that compound **4d** liposome was stable in plasma and could reduce the toxicity of paclitaxel in systemic circulation, indicating that compound **4d** liposome could effectively release paclitaxel in tumor tissue to achieve targeted anti-tumor effect.

### 2.6. Study of Palictaxel Derivative Loaded Liposomes In Vivo

**Figure 6:**
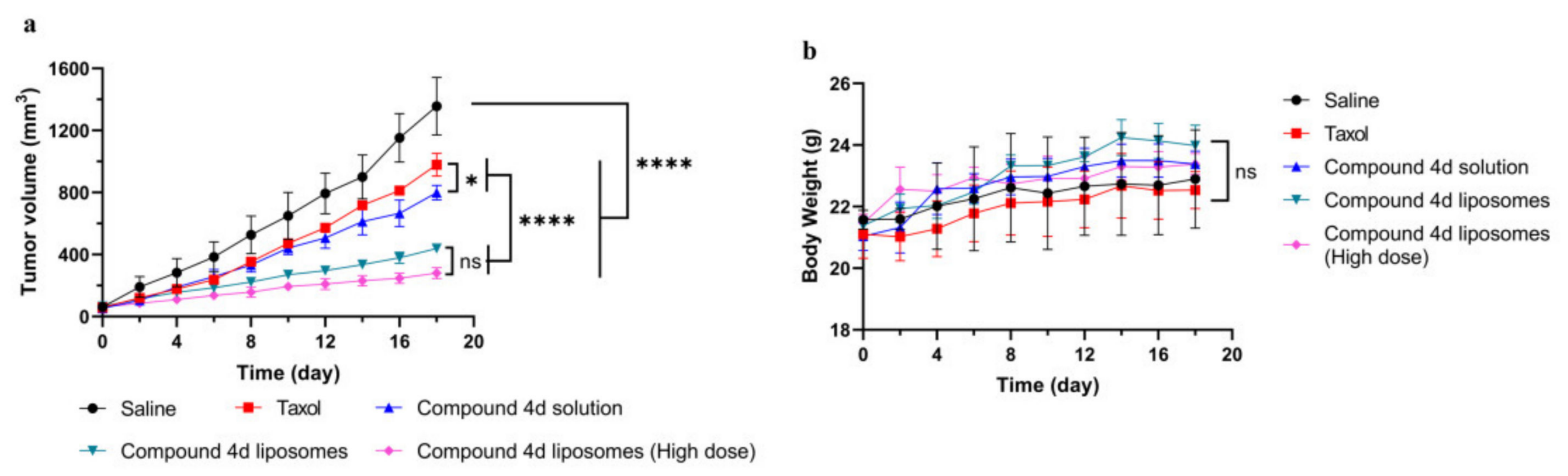
Compound **4d** liposome against 4T1 xenograft tumor in vivo. (**a**) Tumor growth profiles. (**b**) Body weight. All data are presented as mean ± SD (*n* = 5), * *p* < 0.05 and **** *p* < 0.0001 by two-tailed Student’s *t* test. “ns” mean “no significance”.

A549 and HCT-116 cells are human tumor cells, while 4T1 cells are mouse tumor cells. Based on the availability of the tumor model, mice bearing a 4T1 xenograft tumor were used to study the in vivo antitumor efficacy of the compound **4d** liposome. As depicted in Figure 6a, there was difference between compound **4d** (8 mg/kg) and the paclitaxel control group (8 mg/kg). Furthermore, compound **4d** liposome (8 mg/kg) significantly inhibited tumor growth, which may be due to its better stability, better drug delivery advantages and more effective drug release from tumor cells. When administered with a higher dose of compound **4d** liposome (24 mg/kg), the tumor volume further decreased, showing obviously anti-tumor effect in vivo. In addition, according to changes in animal weight, all liposome preparations showed no obvious toxic effects (Figure 6b).

## 3. Materials and Methods

### 3.1. Materials

All chemical materials and solvents were obtained from commercial suppliers without further purification. Intermediates were purified by silica gel column chromatography (200–300 mesh). Nuclear magnetic resonance (NMR) spectra were generated on a Bruker Avance III-600 instrument (600 MHz for ^1^H and 150 MHz for ^13^C), using TMS as an internal standard. High-resolution mass spectra experiments (HR-MS) were performed on an Agilent Accurate-Mass Q-TOF 6530 instrument. Hydrogenated soybean phosphatidylcholine (HSPC), cholesterol (for injection) (chol), and 2-distearoyl-snglycero-3-phosphoethanolamine-N-[methyl(polyethyleneglycol)-2000 (DSPE-PEG2000) were purchased from Shanghai Advanced Vehicle Technology Pharmaceutical Ltd. (Shanghai, China).

### 3.2. Chemistry

#### 3.2.1. General Synthesis Procedure for Intermediates **1a–1d**

Compounds **1a–1d** were prepared according to the literature [27]. Diacids (2.89 mmol) were added in acetic anhydride (5 mL), followed by nitrogen protection. The reaction was stirred at room temperature for 2 h. Then, dry toluene (10 mL) was added and the solvent was removed by reduced pressure. The product did not need to be purified.

#### 3.2.2. General Synthesis Procedure (A) for Esterification

(4-(4,4,5,5-tetramethyl-1,3,2-dioxaborolan-2-yl)phenyl)methanol (2.03 mmol), acid anhydride (6.09 mmol) and DMAP (0.61 mmol) were added to dry DCM (10 mL), followed by nitrogen protection. The reaction was stirred at room temperature for 3 h and monitored by TLC. The mixture was washed with 2 M HCl solution and extracted with DCM three times. The combined organic layer was dried over Na_2_SO_4_ and evaporated in a vacuum. The crude product was purified by silica gel column chromatography (petroleum ether: acetone = 10:1) to obtain the product.

#### 3.2.3. General Synthesis Procedure (B) for Esterification

Carboxylic acid (0.35 mmol) and DMAP (0.04 mmol) were added to dry DCM (10 mL). The mixture was stirred at −20 °C for 10 min, followed by adding EDCI (0.35 mmol). Then, the reaction was stirred at −20 °C for 30 min, followed by adding paclitaxel or docetaxel (0.12 mmol). Then, the reaction was stirred at −20 °C for 60 min, and transferred to room temperature for 5–8 h. The mixture was washed with 2 M HCl solution and extracted with DCM three times. The combined organic layer was dried over Na_2_SO_4_ and evaporated in a vacuum. The crude product was purified by thin layer silica gel column chromatography (dichloromethane: methanol = 20:1) to obtain the product.

#### 3.2.4. General Synthesis Procedure (C) for the Hydrolysis Reaction

To a solution of boronic acid ester (0.13 mmol) in acetone (5 mL), a solution of sodium periodate (0.52 mmol) and ammonium acetate (0.65 mmol) in water (5 mL) was added and the reaction was stirred at room temperature for 8 h. The solid was precipitated after acetone was evaporated in a vacuum. The crude product was purified by thin layer silica gel column chromatography (dichloromethane: methanol = 20:1) to obtain the product.

#### 3.2.5. General Synthesis Procedure (D) for the Arbuzov Reaction

CuCl (0.39 mmol), bis(pinacolato)diboron (1.97 mmol) and PCy_3_ (0.39 mmol) were added to DCM (20 mL), followed by nitrogen protection. Then, *t*-BuOK (1.97 mmol) was added and the reaction was at 0 °C for 30 min. Finally, bromide (1.31 mmol) was added and the reaction was at 0 °C for 30 min. The mixture was washed with water and extracted with DCM three times. The combined organic layer was dried over anhydrous sodium sulfate and evaporated in a vacuum. The crude product was purified by silica gel column chromatography (petroleum ether: acetone = 100:1) to obtain the product.

#### 3.2.6. General Synthesis Procedure (E) for Esterification

Alcohol (5.13 mmol), pyridine (14.89 mmol) and 4-nitrophenyl chloroformate (7.66 mmol) were added to dry DCM (10 mL), followed by nitrogen protection. The reaction was stirred at −20 °C for 5 h. The mixture was washed with 2 M HCl solution and extracted with DCM three times. The combined organic layer was dried over Na_2_SO_4_ and evaporated in a vacuum. The crude product was purified by silica gel column chromatography (petroleum ether: acetone = 100:3) to obtain the product.

#### 3.2.7. General Synthesis Procedure (F) for Esterification

Paclitaxel or docetaxel (0.12 mmol), DMAP (0.36 mmol) and ester (0.36 mmol) were added to dry DCM (10 mL). Then, the reaction was stirred at room temperature for 2 h. The mixture was washed with 2 M HCl solution and extracted with DCM three times. The combined organic layer was dried over Na_2_SO_4_ and evaporated in a vacuum. The crude product was purified by thin layer silica gel column chromatography (dichloromethane: methanol = 20:1) to obtain the product.

#### 3.2.8. General Synthesis Procedure (G) for the Hydrolysis Reaction

To a solution of methyl carboxylate (2.20 mmol) in methanol (5 mL), 2 N sodium hydroxide solution (10 mL) was added. The reaction was stirred at room temperature for 3 h. The mixture was added to 2 M HCl solution, adjusted to pH = 3, and extracted with DCM three times. The combined organic layer was dried over Na_2_SO_4_ and evaporated in a vacuum. The product did not need to be purified.

#### 3.2.9. Preparation of Compound **4a–4e** (Exemplified by **4a**)

4-oxo-4-((4-(4,4,5,5-tetramethyl-1,3,2-dioxaborolan-2-yl)benzyl)oxy)butanoic acid (**2a**).

Following general procedure A, compound **2a** was obtained from compound **1a** and 4-hydroxymethyl phenylborate pinacol ester (0.53g, 78%).^1^H-NMR (600 MHz, DMSO-*d*_6_) *δ*_H_ 12.20 (s, 1H), 7.66 (d, *J* = 8.0 Hz, 2H), 7.36 (d, *J* = 7.8 Hz, 2H), 5.12 (s, 2H), 2.59–2.57 (m, 2H), 2.49–2.46 (m, 2H), 1.29 (s, 12H).

2’-*O*-[4-((4-(4,4,5,5-tetramethyl-1,3,2-dioxaborolan-2-yl))oxy)-4-oxobutyryl] taxol (**3a**).

Following general procedure B, compound **3a** was obtained from compound **2a** and palictaxel (0.13g, 24%). ^1^H-NMR (600 MHz, CDCl_3_) *δ*_H_ 8.14 (d, *J* = 7.2 Hz, 2H), 7.78 (d, *J* = 7.2 Hz, 4H), 7.60 (t, *J* = 7.2 Hz, 1H), 7.53–7.47 (m, 3H), 7.42–7.37 (m, 6H), 7.34–7.31 (m, 1H), 7.25–7.24 (m, 2H), 7.06 (d, *J* = 9.2 Hz, 1H), 6.29 (s, 1H), 6.24 (t, *J* = 8.4 Hz, 1H), 5.99 (dd, *J* = 9.1, 3.0 Hz, 1H), 5.68 (d, *J* = 7.1 Hz, 1H), 5.50 (d, *J* = 3.1 Hz, 1H), 4.99 (s, 2H), 4.97 (dd, *J* = 9.7, 2.3 Hz, 1H), 4.44 (dd, *J* = 11.0, 6.6 Hz, 1H), 4.31 (d, *J* = 8.4 Hz, 1H), 4.20 (d, *J* = 8.4 Hz, 1H), 3.81 (d, *J* = 7.0 Hz, 1H), 2.77–2.75 (m, 2H), 2.66–2.64 (m, 2H), 2.52–2.50 (m, 1H), 2.44 (s, 3H), 2.39–2.35 (m, 2H), 2.22 (s, 3H), 1.93 (s, 3H), 1.88 (ddd, *J* = 14.7, 11.0, 2.3 Hz, 1H), 1.68 (s, 3H), 1.33 (s, 12H), 1.23 (s, 3H), 1.13 (s, 3H).

2’-*O*-[4-((4-boronobenzyl)oxy)-4-oxobutyryl]taxol (**4a**).

Following general procedure C, compound **4a** was obtained from compound **3a** (40 mg, 33%). ^1^H-NMR (600 MHz, CDCl_3_) *δ*_H_ 8.13 (d, *J* = 7.1 Hz, 2H), 7.78 (d, *J* = 7.1 Hz, 2H), 7.74 (d, *J* = 7.8 Hz, 2H), 7.61 (t, *J* = 7.2 Hz, 1H), 7.53–7.51 (m, 4H), 7.42–7.38 (m, 6H), 7.32–7.31 (m, 2H), 7.04 (d, *J* = 9.0 Hz, 1H), 6.32 (s, 1H), 6.22 (t, *J* = 9.7 Hz, 1H), 5.92 (dd, *J* = 9.1, 3.2 Hz, 1H), 5.69 (d, *J* = 6.8 Hz, 1H), 5.45 (d, *J* = 3.3 Hz, 1H), 5.08 (s, 2H), 4.97 (d, *J* = 9.7 Hz, 1H), 4.43 (dd, *J* = 11.0, 6.9 Hz, 1H), 4.32 (d, *J* = 8.5 Hz, 1H), 4.20 (d, *J* = 8.5 Hz, 1H), 3.81 (d, *J* = 7.2 Hz, 1H), 2.81–2.77 (m, 2H), 2.67–2.65 (m, 2H), 2.45–2.43 (m, 1H), 2.41 (s, 3H), 2.38–2.33 (m, 2H), 2.24 (s, 3H), 1.89 (s, 3H), 1.88–1.85 (m, 1H), 1.69 (s, 3H), 1.23 (s, 3H), 1.15 (s, 3H). ^13^C-NMR (150 MHz, CDCl_3_) *δ*c 203.7, 171.9, 171.5, 171.2, 169.9, 168.0, 167.2, 167.0, 142.7, 138.5, 136.9, 133.9 (2C), 133.7, 133.5, 132.8, 132.0, 130.2 (2C), 130.2, 129.1 (2C), 128.7 (4C), 128.5, 127.3 (2C), 127.2 (3C), 126.6 (2C), 84.4, 81.1, 79.1, 76.4, 75.7, 75.0, 74.2, 71.9, 71.8, 66.4, 58.5, 52.8, 45.7, 43.2, 35.7, 35.5, 29.7, 29.2, 29.0, 26.7, 22.6, 20.9, 14.7, 9.6. HR-MS (ESI): *m**/**z* calcd for C_58_H_63_BNO_19_^+^ ([M+H]^+^) 1088.4082, found: 1088.4093.

Compounds **4b–4e** and **6a–6b** were prepared by the same method.

2’-*O*-[4-((4-boronobenzyl)oxy)-4-oxobutyryl]docetaxel (**4b**).

Following general procedure A, B and C, compound **4b** was obtained (35 mg, 6%). ^1^H-NMR (600 MHz, CDCl_3_) *δ*_H_ 8.09 (d, *J* = 7.5 Hz, 2H), 7.74 (d, *J* = 7.5 Hz, 2H), 7.60 (t, *J* = 7.5 Hz, 1H), 7.50 (d, *J* = 7.6 Hz, 2H), 7.36 (d, *J* = 7.6 Hz, 2H), 7.30–7.27 (m, 5H), 6.14 (t, *J* = 9.1 Hz, 1H), 5.65 (d, *J* = 6.7 Hz, 1H), 5.57 (d, *J* = 9.5 Hz, 1H), 5.43 (s, 1H), 5.28 (d, *J* = 9.5 Hz, 1H), 5.24 (s, 1H), 5.11 (s, 2H), 4.95 (dd, *J* = 8.8, 2.0 Hz, 1H), 4.31 (d, *J* = 8.8 Hz, 1H), 4.25 (dd, *J* = 11.0, 6.7 Hz, 1H), 4.18 (d, *J* = 8.4 Hz, 1H), 3.88 (d, *J* = 7.0 Hz, 1H), 2.78–2.75 (m, 1H), 2.70–2.66 (m, 2H), 2.56–2.53 (m, 2H), 2.40 (s, 3H), 2.32–2.28 (m, 2H), 1.99 (s, 3H), 1.97–1.96 (m, 1H), 1.83 (s, 3H), 1.33 (s, 9H), 1.19 (s, 3H), 1.09 (s, 3H). ^13^C-NMR (150 MHz, CDCl_3_) *δ*c 211.2, 172.0, 171.8, 171.4, 168.0, 167.1, 155.3, 138.3, 138.3, 134.0 (2C), 133.7, 130.2 (2C), 128.9 (2C), 128.8 (2C), 128.7 (2C), 128.7, 128.2, 127.4, 127.3, 126.4 (3C), 84.2, 82.1, 80.4, 78.8, 77.9, 76.5, 75.9, 74.9, 74.7, 71.8, 66.5, 57.6, 57.2, 46.4, 43.0, 29.7, 29.1, 28.8, 28.1 (3C), 26.3, 22.6, 20.8, 16.8, 14.2, 9.9. HR-MS (ESI): *m*/*z* calcd for C_54_H_64_BNO_19_Na^+^ ([M+Na]^+^) 1064.4058, found: 1064.4065.

2’-*O*-[5-((4-boronobenzyl)oxy)-5-oxopentanoyl]taxol (**4c**).

Following general procedure A, B and C, compound **4c** was obtained (43 mg, 7%). ^1^H-NMR (600 MHz, CDCl_3_) *δ*_H_ 8.13 (d, *J* = 7.3 Hz, 2H), 7.73 (d, *J* = 7.7 Hz, 2H), 7.71 (d, *J* = 7.8 Hz, 2H), 7.60 (t, *J* = 7.5 Hz, 1H), 7.52–7.46 (m, 3H), 7.41–7.35 (m, 6H), 7.33–7.29 (m, 3H), 7.13 (d, *J* = 9.2 Hz, 1H), 6.30 (s, 1H), 6.23 (t, *J* = 9.0 Hz, 1H), 5.96 (dd, *J* = 9.2, 3.4 Hz, 1H), 5.68 (d, *J* = 7.1 Hz, 1H), 5.49 (d, *J* = 3.5 Hz, 1H), 5.10 (s, 2H), 4.96 (dd, *J* = 9.7, 2.3 Hz, 1H), 4.42 (dd, *J* = 10.9, 6.7 Hz, 1H), 4.30 (d, *J* = 8.4 Hz, 1H), 4.20 (d, *J* = 8.5 Hz, 1H), 3.80 (d, *J* = 7.1 Hz, 1H), 2.56–2.52 (m, 1H), 2.49–2.46 (m, 2H), 2.43 (s, 3H), 2.39–2.33 (m, 2H), 2.21 (s, 3H), 2.04–1.96 (m, 4H), 1.92 (s, 3H), 1.89–1.87(m, 1H), 1.67 (s, 3H), 1.22 (s, 3H), 1.13 (s, 3H). ^13^C-NMR (150 MHz, CDCl_3_) *δ*c 203.8, 172.7, 171.9, 171.3, 169.8, 168.2, 167.4, 167.0, 142.6, 138.3, 136.8, 134.0, 133.7, 133.5, 132.8, 132.0, 130.2 (2C), 129.2, 129.0 (2C), 128.7 (4C), 128.5, 127.2, 127.1 (4C), 127.1, 126.5, 126.5, 84.4, 81.0, 79.0, 76.4, 75.6, 75.0, 74.1, 72.0, 71.8, 66.1, 58.4, 52.8, 45.6, 43.1, 35.5, 35.5, 32.8, 32.6, 26.7, 22.6, 22.0, 20.8, 20.0, 14.8, 9.6. HR-MS (ESI): *m*/*z* calcd for C_59_H_65_BNO_19_^+^ ([M+H]^+^) 1102.4238, found: 1102.4249.

2’-*O*-[2-((2-((4-boronobenzyl)oxy)-2-oxoethyl)disulfaneyl)acetyl]taxol (**4d**).

Following general procedure A, B and C, compound **4d** was obtained (44 mg, 7%). ^1^H-NMR (600 MHz, CDCl_3_) *δ*_H_ 8.14 (d, *J* = 7.2 Hz, 2H), 7.76 (d, *J* = 7.0 Hz, 2H), 7.73 (d, *J* = 8.4 Hz, 2H), 7.60 (t, *J* = 7.2 Hz, 1H), 7.53–7.48 (m, 4H), 7.43–7.41 (m, 6H), 7.33 (d, *J* = 7.0 Hz, 2H), 7.16 (d, *J* = 9.1 Hz, 1H), 6.29 (s, 1H), 6.23 (t, *J* = 8.4 Hz, 1H), 5.97 (dd, *J* = 9.0, 3.4 Hz, 1H), 5.69 (d, *J* = 7.1 Hz, 1H), 5.50 (d, *J* = 2.9 Hz, 1H), 5.16–5.15 (m, 2H), 4.97 (dd, *J* = 9.7, 2.3 Hz, 1H), 4.43–4.41 (m, 1H), 4.32 (d, *J* = 8.6 Hz, 1H), 4.20 (d, *J* = 8.4 Hz, 1H), 3.80 (d, *J* = 7.4 Hz, 1H), 3.50–3.49 (m, 4H), 2.57–2.52 (m, 1H), 2.42 (s, 3H), 2.35–2.31 (m, 2H), 2.22 (s, 3H), 1.89 (s, 3H), 1.88–1.85 (m, 1H), 1.68 (s, 3H), 1.22 (s, 3H), 1.13 (s, 3H). ^13^C-NMR (150 MHz, CDCl_3_) *δ*_C_ 203.8, 171.5, 170.0, 169.6, 168.4, 167.8, 167.5, 167.0, 142.7, 137.7, 136.7, 134.0 (2C), 133.7 (2C), 132.8, 132.0, 130.2 (2C), 129.2, 129.1 (2C), 128.8 (2C), 128.7 (3C), 128.6, 127.7 (2C), 127.2 (2C), 126.8 (2C), 84.4, 81.2, 79.1, 76.5, 75.7, 75.0 (2C), 72.1, 72.0, 67.4, 58.5, 53.0, 45.7, 43.2, 41.4, 40.3, 35.6, 35.5, 26.8, 22.7, 22.0, 20.9, 14.8, 9.6. HR-MS (ESI): *m*/*z* calcd for C_58_H_63_BNO_19_S_2_^+^ ([M+H]^+^) 1152.3523, found: 1152.3539.

2’-*O*-[3-((3-((4-boronobenzyl)oxy)-3-oxopropyl)disulfaneyl)propionyl]taxol (**4e**).

Following general procedure A, B and C, compound **4e** was obtained (49 mg, 9%). ^1^H-NMR (600 MHz, CDCl_3_) *δ*_H_ 8.13 (d, *J* = 8.1 Hz, 2H), 7.74 (d, *J* = 8.1 Hz, 2H), 7.70 (d, *J* = 6.8 Hz, 2H), 7.61 (t, *J* = 6.7 Hz, 1H), 7.53–7.52 (m, 5H), 7.41–7.37 (m, 6H), 7.32–7.30 (m, 1H), 7.05 (d, *J* = 10.8 Hz, 1H), 6.29 (s, 1H), 6.23 (t, *J* = 9.0 Hz, 1H), 5.68 (d, *J* = 7.1 Hz, 1H), 5.61 (d, *J* = 7.5 Hz, 1H), 5.53 (d, *J* = 3.5 Hz, 1H), 5.30 (s, 2H), 4.96 (dd, *J* = 11.7, 2.4 Hz, 1H), 4.42 (dd, *J* = 10.9, 6.7 Hz, 1H), 4.31 (d, *J* = 8.6 Hz, 1H), 4.20 (d, *J* = 8.3 Hz, 1H), 3.80 (d, *J* = 6.9 Hz, 1H), 2.90–2.88 (m, 2H), 2.86–2.81 (m, 4H), 2.76–2.72 (m, 2H), 2.58–2.52 (m, 1H), 2.43 (s, 3H), 2.37–2.31(m, 2H), 2.17 (s, 3H), 1.88–1.85 (m, 1H), 1.79 (s, 3H), 1.68 (s, 3H), 1.21 (s, 3H), 1.12 (s, 3H). ^13^C-NMR (150 MHz, CDCl_3_) *δ*c 203.8, 171.5, 170.0, 169.6, 168.4, 167.8, 167.4, 167.0, 142.7, 137.7, 136.7, 134.0 (2C), 133.7 (2C), 133.6, 132.8, 132.0, 130.2 (2C), 129.1, 128.8 (2C), 128.7 (3C), 128.6, 128.5, 127.7 (2C), 127.2 (2C), 126.8 (2C), 84.4, 81.2, 79.1, 76.5, 75.7, 75.0 (2C), 72.1, 72.0, 67.4, 58.5, 53.0, 45.7, 43.2, 35.6 (2C), 35.5, 30.8, 26.8 (2C), 26.5, 22.7, 22.0, 20.9, 14.8, 9.6. HR-MS (ESI): *m/z* calcd for C_60_H_67_BNO_19_S_2_^+^ ([M+H]^+^) 1180.3836, found: 1180.3837.

2’-*O*-[4-boronobenzoyl]taxol (**6a**).

Following general procedure B and C, compound **6a** was obtained (44 mg, 10%). ^1^H-NMR (600 MHz, CDCl_3_) *δ_H_* 8.12 (d, *J* = 6.8 Hz, 2H), 7.99 (d, *J* = 7.9 Hz, 2H), 7.85 (d, *J* = 7.8 Hz, 2H), 7.76 (d, *J* = 7.8 Hz, 2H), 7.60 (t, *J* = 7.2 Hz, 1H), 7.53–7.50 (m, 3H), 7.46–7.40 (m, 6H), 7.34–7.32 (m, 1H),7.06 (d, *J* = 9.1 Hz, 1H), 6.29 (s, 1H), 6.26 (t, *J* = 9.7 Hz, 1H), 6.04 (dd, *J* = 9.0, 3.9 Hz, 1H), 5.70 (d, *J* = 3.9 Hz, 1H), 5.68 (d, *J* = 7.0 Hz, 1H), 4.97 (d, *J* = 9.7 Hz, 1H), 4.45 (dd, *J* = 11.0, 6.6 Hz, 1H), 4.31 (d, *J* = 8.5 Hz, 1H), 4.19 (d, *J* = 8.3 Hz, 1H), 3.81 (d, *J* = 7.1 Hz, 1H), 2.57–2.53 (m, 1H), 2.43 (s, 3H), 2.35–2.31 (m, 2H), 2.23 (s, 3H), 1.96 (s, 3H), 1.91–1.86 (m, 1H), 1.68 (s, 3H), 1.23 (s, 3H), 1.13 (s, 3H). ^13^C-NMR (150 MHz, CDCl_3_) *δ*c 203.8, 171.3 (2C), 169.8, 167.1 (2C), 165.6, 142.8, 136.9, 134.2, 133.9, 133.7, 132.7, 132.0, 130.2 (2C), 130.0, 129.7 (2C), 129.6 (2C), 129.5 (2C), 129.1 (2C), 128.8 (4C), 127.1 (2C), 126.7 (2C), 84.4, 81.0, 79.2, 76.4, 75.6, 75.1, 74.8, 72.1, 67.8, 58.5, 53.8, 45.5, 43.2, 35.5, 31.9, 29.7, 26.8, 22.7, 20.8, 14.1, 9.6. HR-MS (ESI): *m*/*z* calcd for C_54_H_57_BNO_17_^+^ ([M+H]^+^) 1002.3714, found: 1002.3721.

2’-*O*-[4-boronobenzoyl]docetaxel (**6b**).

Following general procedure B and C, compound **6b** was obtained (34 mg, 9%). ^1^H-NMR (600 MHz, CDCl_3_) *δ_H_* 8.10 (d, *J* = 8.2 Hz, 2H), 7.97 (d, *J* = 7.6 Hz, 2H), 7.88 (d, *J* = 7.6 Hz, 1H), 7.82 (d, *J* = 7.6 Hz, 2H), 7.62 (d, *J* = 7.4 Hz, 2H), 7.52–7.50 (m, 2H), 7.38–7.37 (m, 3H), 6.23 (t, *J* = 9.0 Hz, 1H), 5.68 (d, *J* = 7.9 Hz, 1H), 5.54 (s, 2H), 5.46 (d, *J* = 7.0 Hz, 1H), 5.22 (s, 1H), 4.96 (d, *J* = 9.4 Hz, 1H), 4.31 (d, *J* = 7.8 Hz, 1H), 4.27 (dd, *J* = 7.3, 5.7 Hz, 1H), 4.19 (d, *J* = 8.7 Hz, 1H)), 3.93 (d, *J* = 6.6 Hz, 1H), 2.60–2.54 (m, 1H), 2.42 (s, 3H), 2.33–2.28 (m, 2H), 1.97 (s, 3H), 1.91–1.88 (m, 1H), 1.74 (s, 3H), 1.34 (s, 9H), 1.21 (s, 3H), 1.11 (s, 3H). ^13^C-NMR (150 MHz, CDCl_3_) *δ*c 211.2, 173.3, 169.9, 167.9, 167.0, 155.3, 139.0, 137.3 (2C), 135.6, 133.7, 130.2 (2C), 130.1 (2C), 130.0, 129.2, 128.8 (2C), 128.7 (2C), 128.2, 126.7, 126.4 (3C), 84.3, 81.1, 78.8, 76.5, 75.0, 74.4, 74.3, 72.1, 72.0, 71.7, 57.5, 57.2, 46.5, 43.1, 35.7, 29.3, 28.1 (3C), 26.3, 22.6, 20.8, 14.2, 9.9. HR-MS (ESI): *m/z* calcd for C_50_H_59_BNO_17_^+^ ([M+H]^+^) 956.3871, found: 956.3862.

#### 3.2.10. Preparation of Compounds **10a–10c** and **13a–13b** (Exemplified by **10b**)

6-(4,4,5,5-tetramethyl-1,3,2-dioxaborolan-2-yl)hexan-1-ol (**7b**).

Following general procedure D, compound **7b** was obtained from bromide (0.77 g, 65%).

4-nitrophenyl (6-(4,4,5,5-tetramethyl-1,3,2-dioxaborolan-2-yl)hexyl) carbonate (**8b**).

Following general procedure E, compound **8b** was obtained from **7b** (0.51 g, 77%). ^1^H-NMR (600 MHz, CDCl_3_) *δ*_H_ 8.21 (d, *J* = 9.1 Hz, 2H), 7.31 (d, *J* = 9.1 Hz, 2H), 4.21 (t, *J* = 6.7 Hz, 2H), 1.71–1.66 (m, 2H), 1.39–1.33 (m, 4H), 1.31–1.27 (m, 2H), 1.18 (s, 12H), 0.72 (t, *J* = 7.7 Hz, 2H).

2’-*O*-[6-(4,4,5,5-tetramethyl-1,3,2-dioxaborolan-2-yl)hexyl carbonate]taxol (**9b**).

Following general procedure F, compound **9b** was obtained from **8b** and paclitaxel (0.13 g, 33%).

2’-*O*-[6-boronohexyl carbonate]taxol (**10b**).

Following general procedure C, compound **10b** was obtained from **9b** (53 mg, 43%). ^1^H-NMR (600 MHz, CDCl_3_) *δ*_H_ 8.13 (d, *J* = 7.1 Hz, 2H), 7.74 (d, *J* = 7.1 Hz, 2H), 7.60 (t, *J* = 7.1 Hz, 1H), 7.52–7.49 (m, 3H), 7.42–7.39 (m, 6H), 7.36 -7.34 (m, 1H), 7.07 (d, *J* = 9.3 Hz, 1H), 6.30 (s, 1H), 6.25 (t, *J* = 9.1 Hz, 1H), 5.97 (dd, *J* = 9.1, 3.7 Hz, 1H), 5.69 (d, *J* = 7.2 Hz, 1H), 5.44 (d, *J* = 3.1Hz, 1H), 4.97 (dd, *J* = 9.6, 2.4 Hz, 1H), 4.31 (d, *J* = 8.5 Hz, 1H), 4.20 (d, *J* = 8.4 Hz, 1H), 4.13–4.10 (m, 1H), 3.81 (d, *J* = 7.1 Hz, 1H), 2.57–2.52 (m, 1H), 2.45 (s, 3H), 2.40–2.36 (m, 2H), 2.22 (s, 3H), 2.00–1.97 (m, 2H), 1.92 (s, 3H), 1.91–1.86 (m, 1H), 1.68 (s, 3H), 1.67–1.63 (m, 2H), 1.37–1.29 (m, 6H), 1.23 (s, 3H), 1.14 (s, 3H), 0.73 (t, *J* = 7.6 Hz, 2H). ^13^C-NMR (150 MHz, CDCl_3_) *δ*c 203.7, 171.3, 169.9, 168.1, 167.3, 167.0, 154.2, 142.5, 136.7, 133.7, 133.5, 132.8, 132.0, 130.2 (2C), 129.2, 129.1 (2C), 128.7 (4C), 128.5, 127.2 (2C), 126.6 (2C), 84.4, 81.1, 79.1, 76.6, 76.4, 75.6, 75.0, 72.1, 72.0, 69.4, 58.4, 52.8, 45.6, 43.2, 35.6, 35.5, 31.4, 29.7, 28.1, 26.8, 25.2, 23.9, 22.6, 22.1, 20.8, 14.8, 9.6. HR-MS (ESI): *m*/*z* calcd for C_54_H_65_BNO_18_^+^ ([M+H]^+^) 1026.4289, found: 1026.4290.

Compounds **10a**, **10c** and **13a–13b** were prepared by the same method.

2’-*O*-[5-boronopentyl carbonate]taxol (**10a**).

Following general procedure D, E, F and C, compound **10a** was obtained (54 mg, 7%). ^1^H-NMR (600 MHz, CDCl_3_) *δ*_H_ 8.13 (d, *J* = 6.7 Hz, 2H), 7.74 (d, *J* = 6.7 Hz, 2H), 7.61 (t, *J* = 6.7 Hz, 1H), 7.53–7.50 (m, 3H), 7.43–7.39 (m, 5H), 7.37–7.34 (m, 2H), 7.02 (d, *J* = 9.3 Hz, 1H), 6.30 (s, 1H), 6.22 (t, *J* = 9.7 Hz, 1H), 5.98 (dd, *J* = 9.3, 3.1 Hz, 1H), 5.69 (d, *J* = 7.1 Hz, 1H), 5.45 (d, *J* = 3.2 Hz, 1H), 4.97 (dd, *J* = 9.6, 2.4 Hz, 1H), 4.43 (dd, *J* = 10.9, 6.7 Hz, 1H), 4.32 (d, *J* = 8.5 Hz, 1H), 4.20 (d, *J* = 8.4 Hz, 1H), 3.81 (d, *J* = 7.1 Hz, 1H), 2.58–2.53 (m, 1H), 2.45 (s, 3H), 2.40–2.35 (m, 2H), 2.23 (s, 3H), 2.22–2.19 (m, 2H), 1.92 (s, 3H), 1.89–1.87 (m, 1H), 1.68 (s, 3H), 1.65–1.62 (m, 2H), 1.41–1.35 (m, 4H), 1.23 (s, 3H), 1.14 (s, 3H), 0.78 (t, *J* = 7.5 Hz, 2H). ^13^C-NMR (150 MHz, CDCl_3_) *δ*c 203.7, 171.3, 169.9, 168.1, 167.1, 167.0, 154.2, 142.5, 136.8, 133.7, 133.6, 132.8, 132.0, 130.2 (2C), 129.1 (3C), 128.7 (4C), 128.5, 127.2 (2C), 126.7 (2C), 84.4, 81.1, 79.2, 76.6, 76.4, 75.6, 75.0, 72.1, 72.0, 69.3, 58.5, 52.8, 45.7, 43.2, 35.6, 31.9, 29.7, 28.1 (2C), 26.8, 23.6, 22.6, 22.1, 20.8, 14.8, 9.6. HR-MS (ESI): *m*/*z* calcd for C_53_H_63_BNO_18_^+^ ([M+H]^+^) 1012.4133, found: 1012.4160.

2’-*O*-[6-boronohexyl carbonate] docetaxel (**10c**).

Following general procedure D, E, F and C, compound **10c** was obtained (36 mg, 6%).^1^H-NMR (600 MHz, CDCl_3_) *δ*_H_ 8.11 (d, *J* = 7.9 Hz, 2H), 7.60 (t, *J* = 7.5 Hz, 1H), 7.50 (d, *J* = 7.5 Hz, 2H), 7.41–7.38 (m, 2H), 7.35–7.31 (m, 3H), 6.25 (t, *J* = 8.9 Hz, 1H), 5.68 (d, *J* = 6.7 Hz, 1H), 5.62–5.52 (m, 1H), 5.45 (s, 1H), 5.29–5.23 (m, 2H), 4.96 (d, *J* = 9.0 Hz, 1H), 4.32 (d, *J* = 8.8 Hz, 1H), 4.21–4.18 (m, 1H), 4.10–4.06 (m, 1H), 3.92 (d, *J* = 7.1 Hz, 1H), 2.57–2.51 (m, 1H), 2.43 (s, 3H), 2.35–2.31 (m, 2H), 2.25–2.20 (m, 2H), 1.94 (s, 3H), 1.87–1.85 (m, 1H), 1.74 (s, 3H), 1.68–1.63 (m, 2H), 1.39–1.36 (m, 6H), 1.33 (s, 9H), 1.25 (s, 3H), 1.23 (s, 3H), 0.77 (t, *J* = 7.3 Hz, 2H). ^13^C-NMR (150 MHz, CDCl_3_) *δ*c 202.2, 169.7, 168.0, 167.0, 167.0, 154.2, 137.3, 133.6, 130.2 (2C), 128.9 (2C), 128.8 (2C), 128.7 (2C), 128.2, 126.5 (3C), 84.3, 81.0, 80.6, 79.3, 78.9, 76.6, 76.1, 74.7, 74.5, 71.8, 69.2, 58.3, 57.5, 46.4, 45.3, 43.6, 43.1, 41.7, 40.9, 35.6, 29.7, 28.1 (3C), 26.9, 26.3, 21.5, 20.9, 14.1, 9.9. HR-MS (ESI): *m*/*z* calcd for C_50_H_67_BNO_18_^+^ ([M+H]^+^) 980.4446, found: 980.4452.

2’-*O*-[4-boronobenzyl carbonate]taxol (**13a**).

Following general procedure E, F and C, compound **13a** was obtained (36 mg, 13%). ^1^H-NMR (600 MHz, CDCl_3_) *δ*_H_ 8.14 (d, *J* = 7.2 Hz, 2H), 7.73 (d, *J* = 8.4 Hz, 2H), 7.60 (t, *J* = 7.2 Hz, 1H), 7.51–7.48 (m, 5H), 7.43–7.35 (m, 9H), 6.93 (d, *J* = 9.3 Hz, 1H), 6.30 (s, 1H), 6.25 (t, *J* = 8.4 Hz, 1H), 5.99 (dd, *J* = 9.0, 3.4 Hz, 1H), 5.69 (d, *J* = 7.1 Hz, 1H), 5.48 (d, *J* = 2.9 Hz, 1H), 5.28–5.22 (m, 2H), 4.98 (dd, *J* = 9.7, 2.3 Hz, 1H), 4.44–4.42 (m, 1H), 4.32 (d, *J* = 8.6 Hz, 1H), 4.20 (d, *J* = 8.4 Hz, 1H), 3.82 (d, *J* = 7.4 Hz, 1H), 2.59–2.53 (m, 1H), 2.46 (s, 3H), 2.40–2.38 (m, 2H), 2.22 (s, 3H), 1.97–1.94 (m, 1H), 1.87 (s, 3H), 1.68 (s, 3H), 1.23 (s, 3H), 1.14 (s, 3H). ^13^C-NMR (100 MHz, CDCl_3_) *δ*c 203.8, 171.3, 169.9, 167.8, 167.2, 167.0, 154.1, 142.5, 136.6, 134.1, 133.7, 133.5, 132.8, 132.0, 130.2 (2C), 129.2, 129.1 (2C), 128.7 (2C), 128.7 (3C), 128.6, 127.6, 127.1 (4C), 126.7, 126.6 (2C), 84.4, 81.1, 79.1 (2C), 77.1, 76.5, 75.6, 75.1, 72.2, 70.6, 58.5, 52.8, 45.7, 43.2, 35.6, 29.7, 27.2, 26.8, 22.6, 20.8, 14.8, 9.6. HR-MS (ESI): *m*/*z* calcd for C_55_H_59_BNO_18_^+^ ([M+H]^+^) 1032.3820, found: 1032.3833.

2’-*O*-[4-boronobenzyl carbonate] docetaxel (**13b**).

Following general procedure E, F and C, compound **13b** was obtained (36 mg, 11%). ^1^H-NMR (600 MHz, CDCl_3_) *δ*_H_ 8.21 (d, *J* = 8.2 Hz, 2H), 8.09 (d, *J* = 8.2 Hz, 2H), 7.71 (t, *J* = 7.6 Hz, 1H), 7.59 (d, *J* = 7.6 Hz, 2H), 7.49 (d, *J* = 7.3 Hz, 2H), 7.39–7.36 (m, 2H), 7.33–7.30 (m, 3H), 6.20 (t, *J* = 9.0 Hz, 1H), 5.66 (d, *J* = 7.9 Hz, 1H), 5.51 (s, 1H), 5.45 (d, *J* = 7.0 Hz, 1H), 5.30 (s, 1H), 5.18 (s, 1H), 5.08 (d, *J* = 7.0 Hz, 1H), 4.96 (d, *J* = 9.6 Hz, 1H), 4.31 (d, *J* = 8.7 Hz, 1H), 4.26–4.23 (m, 1H), 4.18 (d, *J* = 8.7 Hz, 1H)), 3.88 (d, *J* = 6.6 Hz, 1H), 2.59–2.53 (m, 1H), 2.42 (s, 3H), 2.35–2.21 (m, 2H), 1.88 (s, 3H), 1.87–1.82 (m, 1H), 1.73 (s, 3H), 1.26 (s, 9H), 1.21 (s, 3H), 1.09 (s, 3H). ^13^C-NMR (150 MHz, CDCl_3_) *δ*c 211.2, 173.3, 169.9, 167.9, 167.0, 155.3, 139.0, 135.6, 133.7 (2C), 130.2 (2C), 130.1, 129.2, 129.1, 128.8 (3C), 128.7 (2C), 128.2, 127.2, 126.7, 126.4 (3C), 84.3, 81.1, 78.8, 76.5, 75.0, 74.4, 74.3, 72.1, 72.0, 71.7, 67.9, 57.5, 57.2, 46.5, 43.1, 35.7, 29.3, 28.1 (3C), 26.3, 22.6, 20.8, 14.2, 9.9. HR-MS (ESI): *m*/*z* calcd for C_51_H_61_BNO_18_^+^ ([M+H]^+^) 986.3976, found: 986.3981.

#### 3.2.11. Preparation of Compound **17a–17g** (Exemplified by **17b**)

Methyl 4-(4,4,5,5-tetramethyl-1,3,2-dioxaborolan-2-yl)butanoate (**14b**).

Following general procedure D, compound **14b** was obtained (0.32 g, 54%). ^1^H-NMR (600 MHz, CDCl_3_) *δ*_H_ 3.65 (s, 3H), 2.32 (t, *J* = 7.6 Hz, 2H), 1.77–1.72 (m, 2H), 1.24 (s, 12H), 0.81 (t, *J* = 7.9 Hz, 2H).

4-(4,4,5,5-tetramethyl-1,3,2-dioxaborolan-2-yl)butanoic acid (**15b**).

Following general procedure G, compound **15b** was obtained from compound **14b** (0.25 g, 85%).

2’-*O*-[4-(4,4,5,5-tetramethyl-1,3,2-dioxaborolan-2-yl)butanoyl]taxol (**16b**).

Following general procedure B, compound **16b** was obtained from compound **15b** and paclitaxel (0.15 g, 44%).

2’-*O*-[4-boronobutanoyl]taxol (**17b**).

Following general procedure C, compound **17b** was obtained from compound **16b** (36 mg, 26%). ^1^H-NMR (600 MHz, CDCl_3_) *δ*_H_ 8.13 (d, *J* = 8.3 Hz, 2H), 7.75 (d, *J* = 8.3 Hz, 2H), 7.61 (t, *J* = 8.3 Hz, 1H), 7.53–7.49 (m, 3H), 7.43–7.37 (m, 6H), 7.35–7.32 (m, 1H), 6.98 (d, *J* = 9.1 Hz, 1H), 6.29 (s, 1H), 6.23 (t, *J* = 8.4 Hz, 1H), 5.96 (dd, *J* = 9.2, 3.6 Hz, 1H), 5.68 (d, *J* = 7.0 Hz, 1H), 5.54 (d, *J* = 3.7 Hz, 1H), 4.97 (dd, *J* = 9.5, 2.2 Hz, 1H), 4.43 (dd, *J* = 10.9, 6.6 Hz, 1H), 4.31 (d, *J* = 8.4 Hz, 1H), 4.19 (d, *J* = 8.4 Hz, 1H), 3.80 (d, *J* = 7.3 Hz, 1H), 2.58–2.53 (m, 1H), 2.45 (s, 3H), 2.43–2.40 (m, 2H), 2.36–2.31 (m, 2H), 2.22 (s, 3H), 1.92 (s, 3H), 1.88 (ddd, *J* = 14.7, 11.0, 2.3 Hz, 1H), 1.76–1.74 (m, 2H), 1.68 (s, 3H), 1.22 (s, 3H), 1.13 (s, 3H), 0.77 (t, *J* = 8.4 Hz, 2H). ^13^C-NMR (100 MHz, CDCl_3_) *δ*c 203.7, 173.0, 171.2, 169.9, 168.5, 167.2, 167.0, 142.6, 136.9, 133.7, 133.6, 132.9, 132.0, 130.2 (2C), 129.2, 129.1(2C), 129.0, 128.7 (3C), 128.5, 127.1 (4C), 84.4, 81.1, 79.1, 76.4, 75.6, 75.1, 73.9, 72.1, 72.0, 58.5, 52.9, 45.6, 43.2, 35.7, 35.5, 29.7, 26.8, 22.7, 22.1, 20.8, 19.7, 14.8, 14.1, 9.6. HR-MS (ESI): *m*/*z* calcd for C_51_H_58_BNO_17_Na^+^ ([M+Na]^+^) 990.3690, found: 990.3698.

Compounds **17a**, **17c–17g** were prepared by the same method.

2’-*O*-[3-boronopropanoyl]taxol (**17a**).

Following general procedure D, G, B and C, compound **17a** was obtained (37 mg, 5%). ^1^H-NMR (600 MHz, CDCl_3_) *δ*_H_ 8.13 (d, *J* = 8.3 Hz, 2H), 7.75 (d, *J* = 8.3 Hz, 2H), 7.61 (t, *J* = 8.3 Hz, 1H), 7.53–7.50 (m, 3H), 7.42–7.37 (m, 7H), 7.06 (d, *J* = 9.3 Hz, 1H), 6.29 (s, 1H), 6.24 (t, *J* = 8.4 Hz, 1H), 5.98 (dd, *J* = 9.2, 3.3 Hz, 1H), 5.68 (d, *J* = 7.0 Hz, 1H), 5.53 (d, *J* = 3.3 Hz, 1H), 4.98 (dd, *J* = 9.5, 2.2 Hz, 1H), 4.43 (dd, *J* = 10.9, 6.6 Hz, 1H), 4.31 (d, *J* = 7.8 Hz, 1H), 4.20 (d, *J* = 8.3 Hz, 1H), 3.80 (d, *J* = 7.0 Hz, 1H), 2.58–2.53 (m, 1H), 2.45 (s, 3H), 2.43–2.41 (m, 2H), 2.39–2.34 (m, 2H), 2.22 (s, 3H), 1.92 (s, 3H), 1.87 (ddd, *J* = 14.7, 11.0, 2.3 Hz, 1H), 1.68 (s, 3H), 1.22 (s, 3H), 1.13 (s, 3H), 0.81 (t, *J* = 8.4 Hz, 2H). ^13^C-NMR (150 MHz, CDCl_3_) *δ*c 210.8, 171.9, 171.1, 169.9, 168.5, 167.5, 166.0, 140.6, 134.2, 130.2 (2C), 129.6, 129.5 (2C), 128.9, 128.9, 128.7 (3C), 128.5, 127.0 (4C), 126.8, 120.7, 115.3, 84.4, 83.3, 79.9, 74.9, 71.2, 70.2, 69.5, 67.8, 58.3, 53.8, 52.7, 45.6, 38.9, 31.7, 30.6, 29.7, 29.3, 29.0, 24.0, 23.0, 22.7, 14.0, 11.1. HR-MS (ESI): *m*/*z* calcd for C_50_H_56_BNO_17_Na^+^ ([M+Na]^+^) 976.3534, found: 976.3550.

2’-*O*-[5-boronopentanoyl]taxol (**17c**).

Following general procedure D, G, B and C, compound **17c** was obtained (45 mg, 6%).^1^H-NMR (600 MHz, CDCl_3_) *δ*_H_ 8.13 (d, *J* = 8.3 Hz, 2H), 7.74 (d, *J* = 8.3 Hz, 2H), 7.60 (t, *J* = 8.3 Hz, 1H), 7.53–7.50 (m, 3H), 7.41–7.38 (m, 6H), 7.35–7.32 (m, 1H), 6.97 (d, *J* = 9.2 Hz, 1H), 6.29 (s, 1H), 6.25 (t, *J* = 8.4 Hz, 1H), 5.97 (dd, *J* = 9.2, 3.6 Hz, 1H), 5.68 (d, *J* = 7.5 Hz, 1H), 5.54 (d, *J* = 3.7 Hz, 1H), 4.97 (dd, *J* = 9.5, 2.2 Hz, 1H), 4.44 (dd, *J* = 10.9, 6.6 Hz, 1H), 4.30 (d, *J* = 8.4 Hz, 1H), 4.19 (d, *J* = 8.4 Hz, 1H), 3.81 (d, *J* = 7.3 Hz, 1H), 2.58–2.53 (m, 1H), 2.46 (s, 3H), 2.40–2.38 (m, 2H), 2.36–2.34 (m, 2H), 2.22 (s, 3H), 2.13–2.10 (m, 2H), 1.93 (s, 3H), 1.88 (ddd, *J* = 14.7, 11.0, 2.3 Hz, 1H), 1.68 (s, 3H), 1.62–1.58 (m, 2H), 1.23 (s, 3H), 1.13 (s, 3H), 0.75 (t, *J* = 8.4 Hz, 2H). ^13^C-NMR (150 MHz, CDCl_3_) *δ*c 203.7, 173.1, 171.2, 169.8, 168.4, 167.2, 167.0, 142.6, 136.9, 133.7, 133.6, 132.8, 132.0, 130.2 (2C), 129.1 (2C), 129.0, 128.7 (3C), 128.5, 127.1 (4C), 126.6, 84.4, 81.0, 79.1, 76.4, 75.6, 75.0, 73.9, 72.0, 71.9, 58.4, 53.4, 45.6, 43.1, 35.5, 33.4, 29.7, 26.8, 26.8, 23.3, 22.7, 22.0, 20.8, 14.8, 10.3, 9.6. HR-MS (ESI): *m/z* calcd for C_52_H_61_BNO_17_^+^ ([M+H]^+^) 982.4027, found: 982.4025.

2’-*O*-[6-boronohexanoyl]taxol (**17d**).

Following general procedure D, G, B and C, compound **17d** was obtained (42 mg, 6%). ^1^H-NMR (600 MHz, CDCl_3_) *δ*_H_ 8.13 (d, *J* = 7.1 Hz, 2H), 7.74 (d, *J* = 7.2 Hz, 2H), 7.61 (t, *J* = 7.2 Hz, 1H), 7.53–7.50 (m, 3H), 7.43–7.37 (m, 5H), 7.35–7.32 (m, 2H), 6.90 (d, *J* = 9.2 Hz, 1H), 6.30 (s, 1H), 6.23 (t, *J* = 9.7 Hz, 1H), 5.94 (dd, *J* = 9.1, 3.9 Hz, 1H), 5.68 (d, *J* = 7.0 Hz, 1H), 5.54 (d, *J* = 3.9 Hz, 1H), 4.97 (dd, *J* = 9.7, 2.3 Hz, 1H), 4.44 (q, *J* = 6.4 Hz, 1H), 4.31 (d, *J* = 8.5 Hz, 1H), 4.19 (d, *J* = 8.5 Hz, 1H), 3.81 (d, *J* = 7.1 Hz, 1H), 2.57–2.53 (m, 1H), 2.44 (s, 3H), 2.42–2.38 (m, 2H), 2.35–2.30 (m, 2H), 2.23 (s, 3H), 2.17–2.13 (m, 2H), 1.93 (s, 3H), 1.91–1.88 (m, 1H), 1.87–1.85 (m, 2H), 1.75–1.72 (m, 2H), 1.68 (s, 3H), 1.23 (s, 3H), 1.13 (s, 3H), 0.74 (t, *J* = 8.6 Hz, 2H). ^13^C NMR (150 MHz, CDCl_3_) *δ*c 203.8, 172.9, 171.3, 169.8, 168.2, 167.1, 167.0, 142.7, 136.9, 133.7 (2C), 132.8, 132.0, 130.2 (2C), 129.2, 129.1 (2C), 128.7 (4C), 128.5, 127.1 (2C), 126.6 (2C), 84.4, 81.1, 79.2, 76.4, 75.6, 75.0, 73.8, 72.1, 71.8, 58.5, 53.0, 45.6, 43.1, 35.6, 33.5, 31.3, 29.7, 26.8, 24.3, 23.7, 22.7, 22.6, 22.0, 20.8, 14.8, 9.6. HR-MS (ESI): *m*/*z* calcd for C_53_H_62_BNO_17_Na^+^ ([M+Na]^+^) 1018.4003, found: 1018.4024.

2’-*O*-[7-boronoheptanoyl]taxol (**17e**).

Following general procedure D, G, B and C, compound **17e** was obtained (52 mg, 7%). ^1^H-NMR (600 MHz, CDCl_3_) *δ*_H_ 8.13 (d, *J* = 6.9 Hz, 2H), 7.74 (d, *J* = 7.1 Hz, 2H), 7.61 (t, *J* = 7.2 Hz, 1H), 7.53–7.50 (m, 3H), 7.43–7.38 (m, 6H), 7.35–7.32 (m, 1H), 6.93 (d, *J* = 9.3 Hz, 1H), 6.30 (s, 1H), 6.22 (t, *J* = 9.7 Hz, 1H), 5.93 (dd, *J* = 9.2, 4.0 Hz, 1H), 5.68 (d, *J* = 7.2 Hz, 1H), 5.54 (d, *J* = 3.9 Hz, 1H), 4.97 (dd, *J* = 9.7, 2.4 Hz, 1H), 4.43 (dd, *J* = 11.0, 6.9 Hz, 1H), 4.31 (d, *J* = 8.5 Hz, 1H), 4.19 (d, *J* = 8.4 Hz, 1H), 3.80 (d, *J* = 7.1 Hz, 1H), 2.58–2.54 (m, 1H), 2.43 (s, 3H), 2.41–2.37 (m, 2H), 2.35–2.30 (m, 2H), 2.23 (s, 3H), 2.16–2.12 (m, 2H), 1.93 (s, 3H), 1.91–1.86 (m, 1H), 1.68 (s, 3H), 1.61–1.56 (m, 2H), 1.36–1.32 (m, 2H), 1.22 (s, 3H), 1.13 (s, 3H), 0.75 (t, *J* = 8.6 Hz, 2H). ^13^C-NMR (150 MHz, CDCl_3_) *δ*c 203.7, 172.9, 171.3, 169.8, 168.2, 167.1, 167.0, 142.6, 136.9, 133.7, 133.6, 132.8, 132.0, 130.2 (2C), 129.2, 129.0 (2C), 128.7 (3C), 128.5, 127.1 (4C), 126.6, 84.4, 81.1, 79.1, 76.4, 75.6, 75.0, 73.8, 72.0, 71.8, 58.5, 53.0, 45.6, 43.1, 35.6, 35.5, 33.7, 31.5, 29.7, 28.4, 26.8, 24.5, 23.9, 22.6, 22.0, 20.8, 14.8, 9.6. HR-MS (ESI): *m/z* calcd for C_54_H_64_BNO_17_Na^+^ ([M+Na]^+^) 1032.4160, found: 1032.4185.

2’-*O*-[4-boronobutanoyl]docetaxel (**17f**).

Following general procedure D, G, B and C, compound **17f** was obtained (32 mg, 4%). ^1^H-NMR (600 MHz, CDCl_3_) *δ*_H_ 8.10 (d, *J* = 7.5 Hz, 2H), 7.60 (t, *J* = 7.5 Hz, 1H), 7.50 (d, *J* = 6.9 Hz, 2H), 7.39–7.37 (m, 2H), 7.32–7.29 (m, 3H), 6.20 (t, *J* = 9.1 Hz, 1H), 5.67 (d, *J* = 6.5 Hz, 1H), 5.54 (d, *J* = 9.2 Hz, 1H), 5.46 (s, 1H), 5.38 (d, *J* =10.9 Hz, 1H), 5.23 (s, 1H), 4.96 (d, *J* = 9.8 Hz, 1H), 4.31 (d, *J* = 8.9 Hz, 1H), 4.26 (dd, *J* = 11.0, 6.7 Hz, 1H), 4.19 (d, *J* = 7.8 Hz, 1H), 3.89 (d, *J* = 7.2 Hz, 1H), 2.55–2.53 (m, 1H), 2.42 (s, 3H), 2.36–2.32 (m, 2H), 2.31–2.29 (m, 2H), 1.92 (s, 3H), 1.88–1.86 (m, 1H), 1.85–1.82 (m, 2H), 1.74 (s, 3H), 1.34 (s, 9H), 1.21 (s, 3H), 1.11 (s, 3H), 0.74 (t, *J* = 8.8 Hz, 2H). ^13^C-NMR (150 MHz, CDCl_3_) *δ*c 211.2, 173.3, 169.9, 168.6, 167.0, 155.3, 135.6, 133.7, 130.2, 130.1 (2C), 129.2, 128.8 (2C), 128.7 (2C), 128.2, 126.4 (3C), 84.3, 81.1, 78.8, 76.5, 75.0, 74.4, 74.3, 72.1, 72.0, 71.7, 57.5, 57.2, 46.5, 43.1, 35.7, 29.7, 28.1 (3C), 27.2, 26.3, 25.8, 22.6, 20.8, 19.5, 14.2, 9.9. HR-MS (ESI): *m*/*z* calcd for C_47_H_61_BNO_17_^+^ ([M+H]^+^) 922.4027, found: 922.4022.

2’-*O*-[6-boronohexanoyl]docetaxel (**17g**).

Following general procedure D, G, B and C, compound **17g** was obtained (32 mg, 4%). ^1^H-NMR (600 MHz, CDCl_3_) *δ*_H_ 8.10 (d, *J* = 7.7 Hz, 2H), 7.61 (t, *J* = 7.5 Hz, 1H), 7.50 (d, *J* = 7.6 Hz, 2H), 7.38 (d, *J* = 7.5 Hz, 2H), 7.30–7.28 (m, 3H), 6.21 (t, *J* = 9.0 Hz, 1H), 5.67 (d, *J* = 7.4 Hz, 1H), 5.49 (s, 1H), 5.45–5.43 (m, 1H), 5.39–5.37 (m, 1H), 5.22 (s, 1H), 4.96 (d, *J* = 9.0 Hz, 1H), 4.31 (d, *J* = 8.8 Hz, 1H), 4.27–4.25 (m, 1H), 4.19 (d, *J* = 8.5 Hz, 1H)), 3.91 (d, *J* = 7.1 Hz, 1H), 2.59–2.52 (m, 1H), 2.42 (s, 3H), 2.33–2.31 (m, 2H), 2.22–2.14 (m, 2H), 1.93 (s, 3H), 1.88–1.84 (m, 1H), 1.78–1.77 (m, 2H), 1.74 (s, 3H), 1.60–1.55 (m, 2H), 1.34 (s, 9H), 1.27–1.25 (m, 2H), 1.21 (s, 3H), 1.11 (s, 3H), 0.76 (t, *J* = 7.7 Hz, 2H). ^13^C-NMR (150 MHz, CDCl_3_) *δ*c 211.4, 173.0, 169.8, 168.3, 167.0, 155.2, 135.5, 133.7, 130.2 (2C), 130.0, 129.2, 128.8 (2C), 128.7 (2C), 128.2, 126.4 (3C), 84.3, 81.0, 80.4, 78.9, 76.5, 75.0, 74.4, 74.3, 74.2, 71.9, 71.8, 57.5, 46.5, 43.1, 33.5, 31.2, 29.7, 29.3, 28.1 (3C), 26.3, 24.3, 23.6, 22.6, 14.2, 14.2, 14.1, 9.9. HR-MS (ESI): *m*/*z* calcd for C_49_H_64_BNO_17_Na^+^ ([M+Na]^+^) 972.4160, found: 972.4160.

### 3.3. MTT Assay In Vitro

Cells were inoculated in 96-well plates (cell density 2 × 10^4^ cells/well) followed by incubation for 24 h at 37 °C. Paclitaxel and target compounds were added to the corresponding well. Subsequently, the cultures were incubated for 72 h. After the MTT solution (50 µL, 2 mg/mL) was added to cultures, the plates were incubated for an additional 4 h. Purple colored formazan crystals were dissolved in 200 µL DMSO, and the absorbance was recorded at 570 nm using Synergy HT, Bio-Tek (Winooski, VT, USA).

### 3.4. Liposome Preparation

Blank liposomes were prepared for membrane hydration. HSPC, chol and DSPE-PEG2000 were dissolved using chloroform at a mass ratio of 3:1:0.02 and then spin-evaporated at 37 °C. The obtained film was then hydrated with 300 mM Cu-Glu (pH 7.4) at 65 °C for 30 min. The multi-layer vesicles were subsequently put into an extruder and passed through polycarbonate membranes with pore sizes of 400 nm, 200 nm and 100 nm 12 times at 65 °C containing 20 mM HEPES, 15 mM EDTA and 300 mM sucrose for removing the un-encapsulated Cu^2+^, and then EDTA was removed with 20 mM HEPES and 300 mM sucrose buffer (pH 7.4, adjusted with triethylamine). Compound **4d** was dissolved in DMSO and slowly added into the liposome at 65 °C for 30 min and then quenched in an ice bath for 5 min (the drug to lipid ratio was 1:10). The encapsulation efficiency and drug loading efficiency of compound **4d** were determined using HPLC. The morphologies of liposomes were characterized using a transition electron microscope (TEM, Hitachi HT7700, Tokyo, Japan); then, their size, PDI and Zeta potential were measured using a Zetasizer Nano ZS90 (Malvern, UK).

### 3.5. In Vitro Drug Release Study

The in vitro drug release of compound **4d** liposome was carried out by dialysis method at 37 °C under a shaking bed (100 rpm). An amount of 1 mL of compound **4d** liposome was added into a dialysis bag and suspended in 25 mL PBS (pH 7.4) containing 15% ethanol (*v*/*v*) and 10 mmol/L EDTA. The release behaviors for redox response were also accomplished as described above, except for the addition of 0, 0.02 μmol/L, 0.1 μmol/L, 1 μmol/L, 10 μmol/L, 100 μmol/L, 1 mmol/L and 10 mmol/L H_2_O_2_. At the predetermined time points, 200 μL samples were taken for analysis and an equal volume of medium was replenished. The concentrations of paclitaxel were determined by HPLC.

### 3.6. In Vivo Antitumor Efficacy and Toxicity

The antitumor effect was evaluated by the nude mice armpit tumor model bearing breast cancer 4T1 cells. When the average tumor volume reached 100 mm^3^, the treatment was initiated at a 3-day interval for 12 days. The mice were randomly divided into five groups (*n* = 5 per group) receiving different palictaxel at 8 mg/kg as follows: saline (the control group), taxol, compound **4d**, compound **4d** liposome, high dose **4d** liposome (3 times the dose, immediately 24 mg/kg). Tumor volume (V = length × width^2^/2) and body weight were recorded. At the end of the experiment, mice were sacrificed. The obtained tumors were accurately weighed to calculate the tumor burden (W_tumor_/W_body_) and tumor inhibition rate [TIR= (W_control tumor_ − W_sample tumor_)/W_control tumor_].

### 3.7. Statistical Analysis

Statistics data were presented as mean ± SD and were representative of at least three independent trials. Multiple-group comparisons were performed using a one-way ANOVA followed by Dunnett’s *t*-test. Two group comparisons were performed using Student’s *t*-test. The treatment effect was considered significant at *p* < 0.05.

## 4. Conclusions

By using a molecular combination strategy to link paclitaxel with boronic acid compounds, a series of new paclitaxel derivatives were designed and synthesized. The antitumor activities of these compounds against A549, HCT-116, 4T1 and LO2 cells in vitro were also determined. As a result, compound **4d**, one of the most effective compounds, showed considerable selectivity to tumor cells. The optimal formulation of compound **4d** liposome, with a drug-lipid ratio of 1:10, was obtained by screening liposomes prescriptions, and the encapsulated efficiency was more than 95%. Meanwhile, compound **4d** liposome (8 mg/kg, four times) and a higher dose of compound **4d** liposome (24 mg/kg, four times) showed better therapeutic effect than paclitaxel (8 mg/kg, four times) in 4T1 tumor models in vivo, and tumor inhibition rates were 74.3%, 81.9% and 58.5%, respectively. In summary, compound **4d**, which connected paclitaxel with boronic acid by using a combination strategy, showed the potential for further research and laid a foundation for exploring the universality of active drug loading of PBA-modified insoluble drugs.

## Figures and Tables

**Table 1:** The chemical structures of compounds **4a–4e**, **6a–6b**, **10a–10c**, **13a–13b** and **17a–17g**.

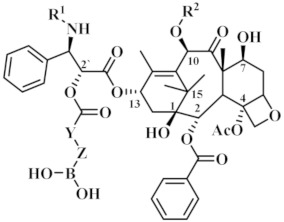				
Compound	R^1^	R^2^	Y	Z
**4a**	Bz	Ac	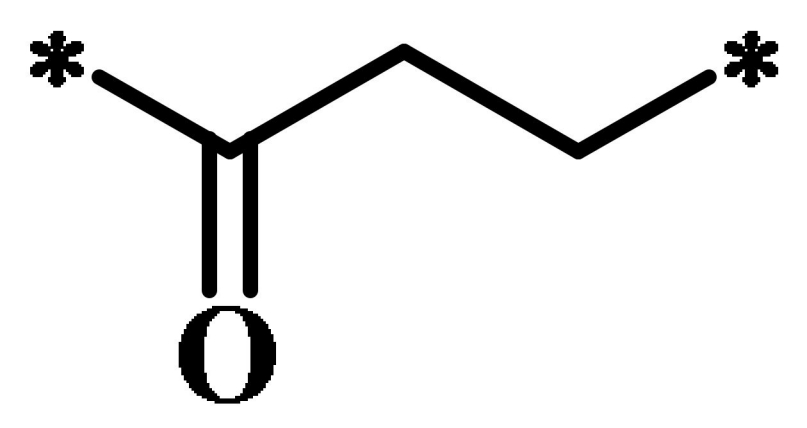	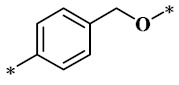
**4b**	Boc	H	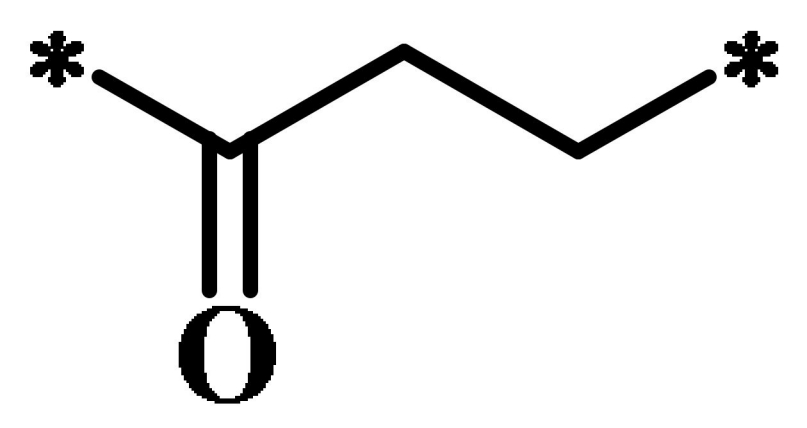	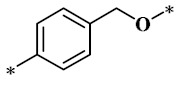
**4c**	Bz	Ac	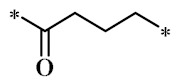	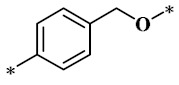
**4d**	Bz	Ac	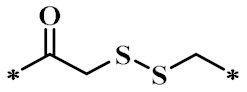	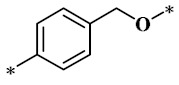
**4e**	Bz	Ac	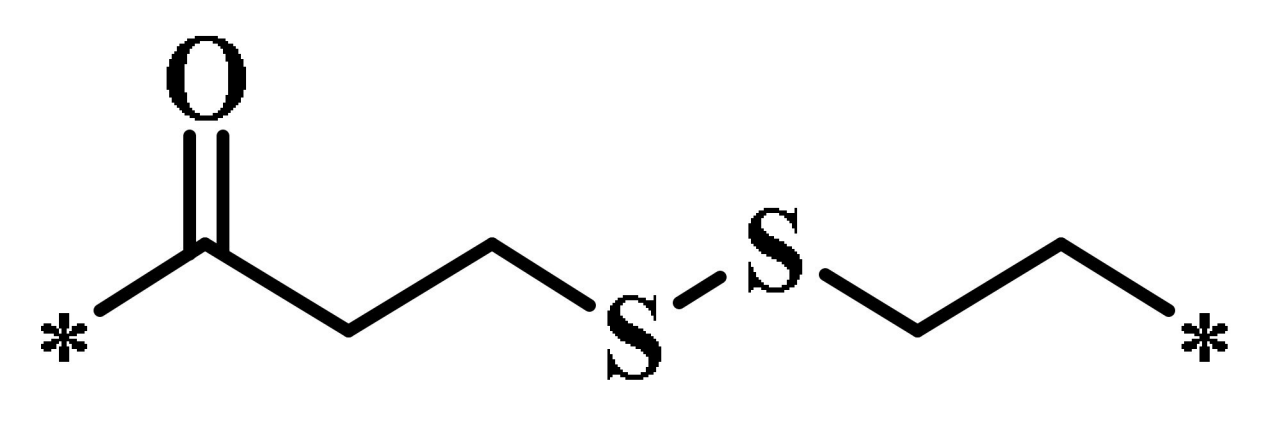	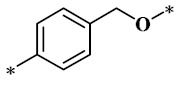
**6a**	Bz	Ac	----	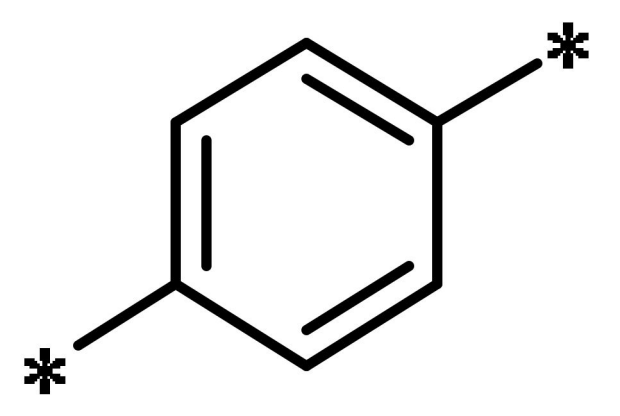
**6b**	Boc	H	----	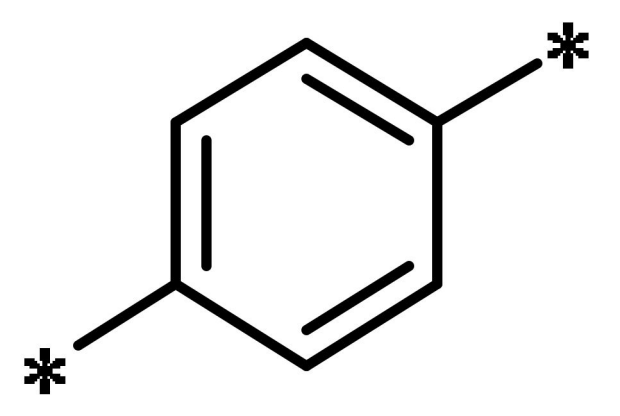
**10a**	Bz	Ac	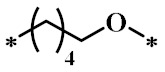	----
**10b**	Bz	Ac	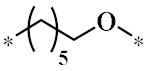	-----
**10c**	Boc	H	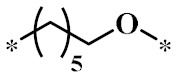	------
**13a**	Bz	Ac	----	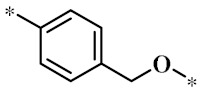
**13b**	Boc	H	----	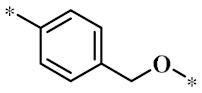
**17a**	Bz	Ac	----	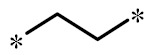
**17b**	Bz	Ac	----	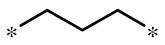
**17c**	Bz	Ac	----	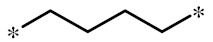
**17d**	Bz	Ac	----	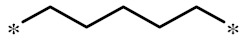
**17e**	Bz	Ac	----	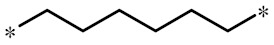
**17f**	Boc	H	----	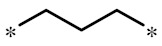
**17g**	Boc	H	----	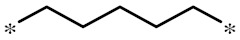

**Table 2:** Inhibitory activity against tumor cells A549, HCT-116, 4T1 and normal cell LO2.

Compound	GI_50_ (nM) ^a^				TI (LO2/A549)	TI (LO2/4T1)	TI (LO2/HCT-116)
	A549	HCT-116	4T1	LO2			
**4a**	**68.49**	**73.71**	20.85%@100 nM	1875	27.4		25.4
**4b**	NT	NT	54.68	NT			
**4c**	NT	NT	>100	NT			
**4d**	13.91	13.01	69.82	638.66	45.9	9.2	49.1
**4e**	NT	NT	>100	NT			
**6a**	>100	>100	>100	NT			
**6b**	NT	NT	>100	NT			
**10a**	>100	>100	>100	32.81%@2 μM			
**10b**	NT	>100	>100	744.90			
**10c**	NT	55.66%@100 nM	35.97%@100 nM	273.42			
**13a**	22.46	8.73	92.86	971.83	43.3	10.5	111.3
**13b**	NT	NT	33.09	NT			
**17a**	21.84	7.98	68.12	714.61	32.7	10.5	89.5
**17b**	69.24	38.27	17.89%@100 nM	38.06%@2 μM			
**17c**	44.76	6.38	87.21	1416.84	31.6	16.3	222.1
**17d**	>100	>100	>100	NT			
**17e**	>100	>100	>100	NT			
**17f**	NT	NT	14.50	NT			
**17g**	NT	NT	35.83%@100 nM	NT			
**paclitaxel**	64.96%@6.25 nM	81.86%@6.25 nM	25.31	69.10		2.7	

^a^ Values are the mean of two independent determinations; NT means “no test”; TI means “therapeutic index”.

**Table 3:** Encapsulation efficiency of copper ion gradients.

HSPC:Chol:DSPE-PEG_2000_ (*w*/*w*/*w*)	Inner Phase (mM)		HBS Buffer	D/L	EE (%)
19:1:0.25	300	Cu-Glu (pH 7.4)	20 mM HEPES + 300 mM sucrose buffer (pH 7.4, adjusted with triethylamine)	1:10	76.34 ± 3.21
3:1:0.05	300				96.13 ± 1.33
3:1:0.05	100				50.34 ± 2.34
3:1:0.05	200				82.34 ± 3.98
3:1:0.05	300	Cu-Glu			85.23 ± 2.34
3:1:0.05	300	Cu(Ac)_2_			71.78 ± 4.32

**Table 4:** Encapsulation efficiency of ATP and saccharides gradients.

Gradients	EE (%)
glucose	75.67 ± 3.89
sucrose	59.425 ± 7.89
lactose	69.87 ± 6.89
maltose	63.71 ± 10.98
mannose	82.77 ± 2.98
trehalose	65.51 ± 8.88
ATP	---

## Supplementary Materials

The following supporting information can be downloaded at: https://www.mdpi.com/article/10.3390/molecules27227967/s1, Table S1. Encapsulation efficiency of calcium acetate gradients; Table S2. Parameters of compound **4d** liposome; Figure S1. Tumor burden of compound **4d** liposome against 4T1 xenograft tumor in vivo; Figure S2. 1H-NMR spectra of compound **2a**; Figure S3. 1H-NMR spectra of compound **3a**; Figure S4. 1H-NMR spectra of compound **8b**; Figure S5. 1H-NMR spectra of compound **14b**; Figure S6–Figure S81: ^1^H-NMR, ^13^C-NMR, HR-MS spectra and HPLC chromatograms of the title compounds.
