# Supplementary material for: Synthesis of Paclitaxel Derivatives for Remote Loading into Liposomes and Improved Therapeutic Effect
Source: Molecules. 2022 Nov 17;27(22):7967. doi: 10.3390/molecules27227967 (PMC9694711; doi:10.3390/molecules27227967)
Supplement: Supplementary file 1 [file molecules-27-07967-s001.zip › molecules-2015680-supplementary.pdf]

Supporting Information

Synthesis of paclitaxel derivatives for remote loading into liposomes and improved therapeutic effect

Xiang-Wei Xu[a, 1], Yan-Hua Liu[b, 1], Shan-Shan Shao[a], Jin-Bo Li[c], Zhao-Chu Xu[c], Yue-Ling Yin[a], Lin-Xiang Zhao[a*], Yong-Jun Wang[c*], Dan Liu[a*]

[a]Key Laboratory of Structure-Based Drugs Design & Discovery of Ministry of Education, Shenyang Pharmaceutical University, Shenyang 110016, China.

[b]Department of Pharmaceutics, School of Pharmacy, Ningxia Medical University, Yinchuan 750004, China

[c]Wuya College of Innovation, Shenyang Pharmaceutical University, Shenyang 110016, China

*Corresponding authors.

E-mail address: sammyld@163.com (D. Liu), wangyongjun@syphu.edu.cn (Y. Wang) and linxiang.zhao@vip.sina.com (L. Zhao);

Tel.: +86-24-4352 0218.

HPLC purity of conditions: Agilent 1260 liquid chromatograph (CH_3CN-H_2O, UV 227 nm, flow rate: 0.5 mL/min).

Table S1. Encapsulation efficiency of calcium acetate gradients.

HSPC:Chol:DSPE-PEG_{2000} (w/w/w)	Con ($Ca(Ac)_2$) (mM)	Con (Na_2SO_4) (mM)	D/L	EE (%)
3:1:0.05	200	200	1:10	---
19:1:0.25	200	200	1:10	---
3:1:0.05	120	120	1:10	---

3:1:0.05	250	250	1:10	---
3:1:0.05	200	200+50 mM Citric acid	1:10	---
3:1:0.05	200	200+100 mM Citric acid	1:10	---

Table S2. Parameters of compound 4d liposome.

Size (nm)	115.6 ± 1.112
PDI	0.065 ± 0.004
Zeta potential (mV)	-14.65 ± 1.891
Loading efficiency (%)	9.02 ± 0.087
EE (%)	96.13 ± 1.33

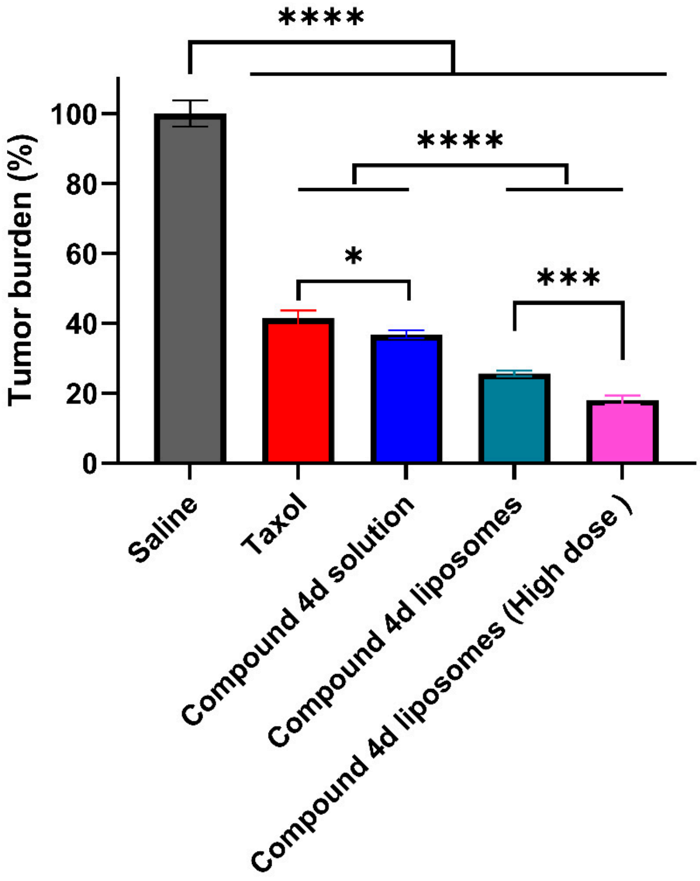

Figure S1. Tumor burden of compound 4d liposome against 4T1 xenograft tumor *in vivo*. All data

are presented as mean ± SD (n = 5), *$P < 0.05$, **$P < 0.01$, ***$P < 0.001$, and ****$P < 0.0001$ by two-tailed Student's *t* test.

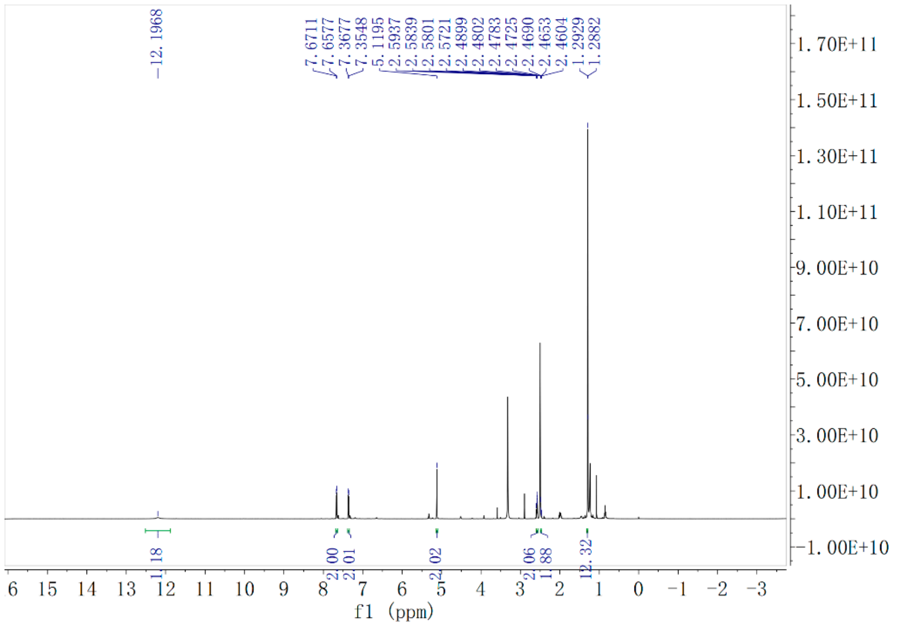

Figure S2. ^{1}H-NMR spectra of compound 2a

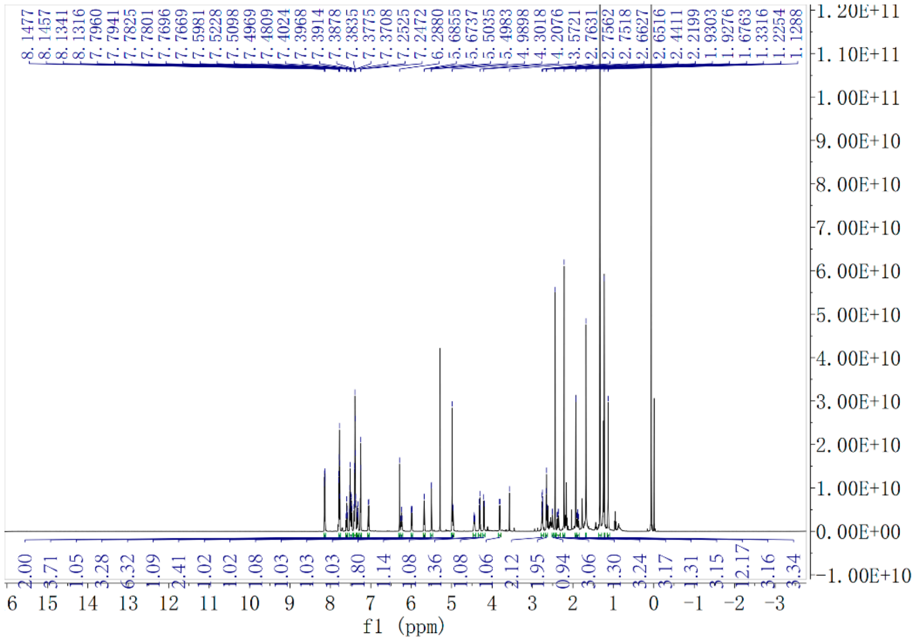

Figure S3. ^{1}H-NMR spectra of compound 3a

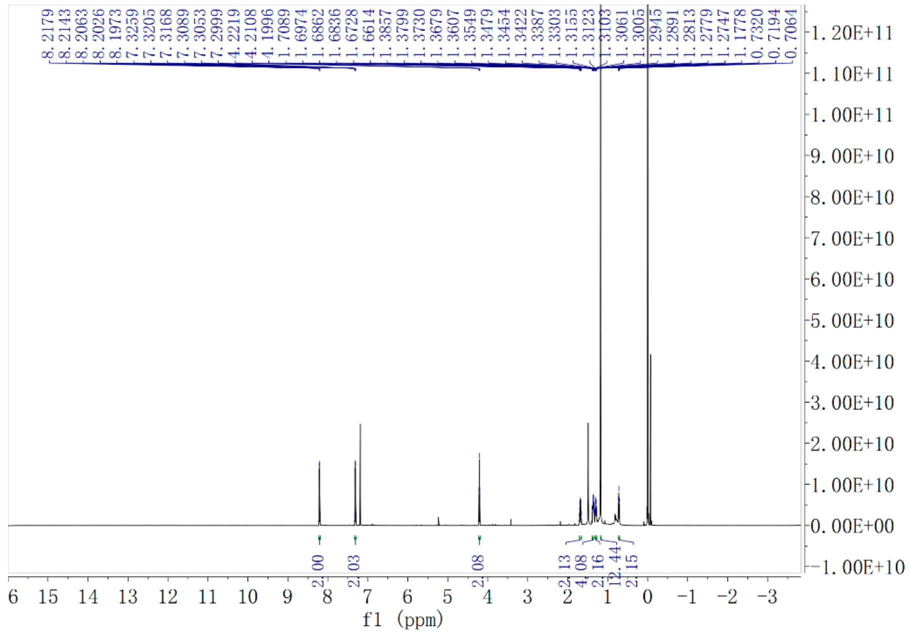

Figure S4. ¹H-NMR spectra of compound 8b

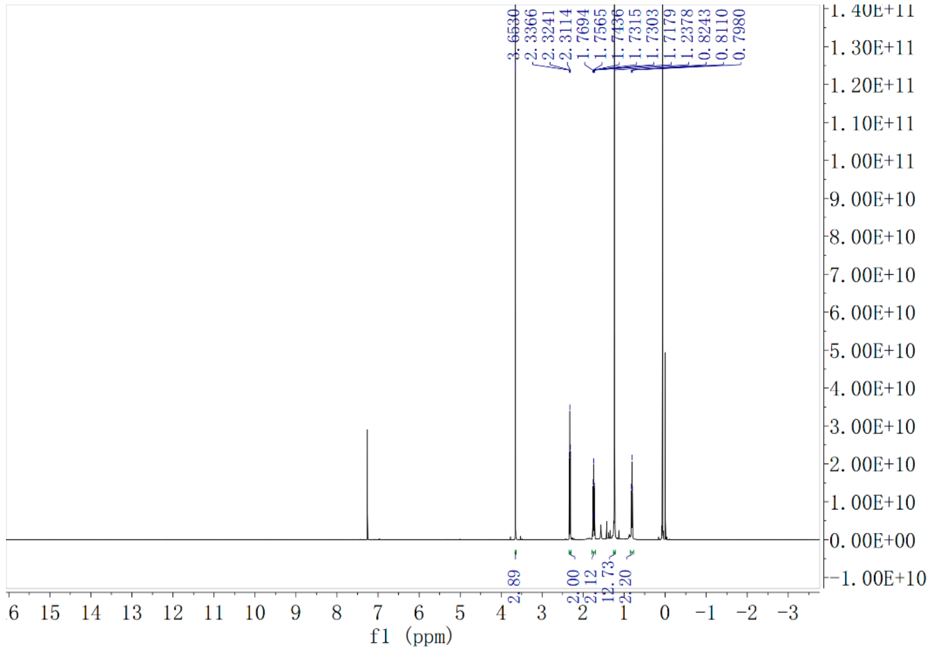

Figure S5. ¹H-NMR spectra of compound 14b

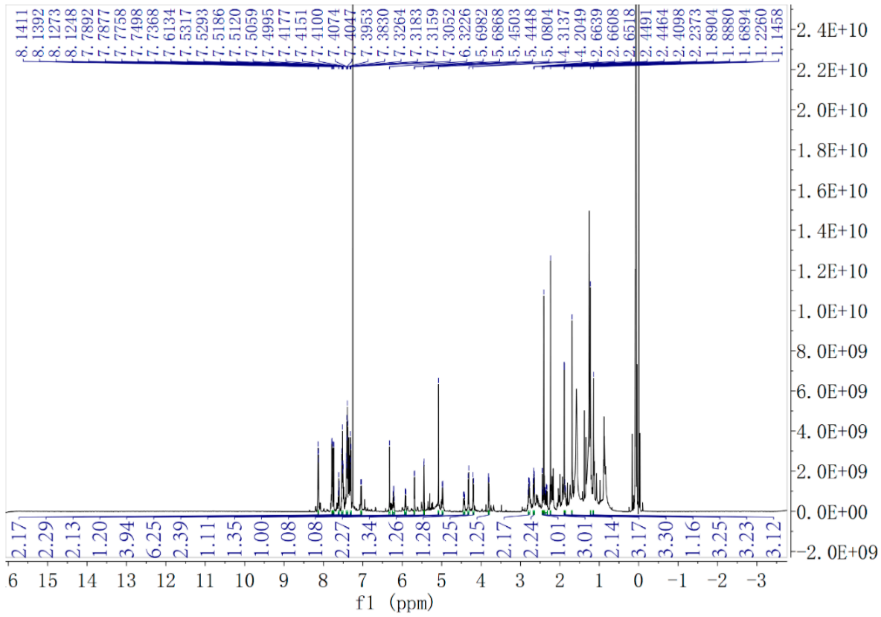

Figure S6. ^{1}H-NMR spectra of compound 4a

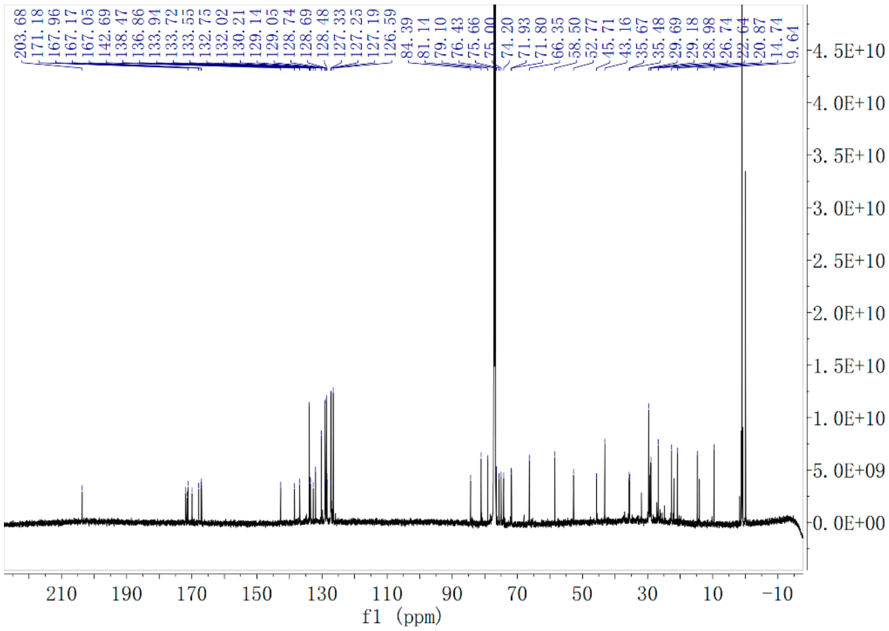

Figure S7. ^{13}C-NMR spectra of compound 4a

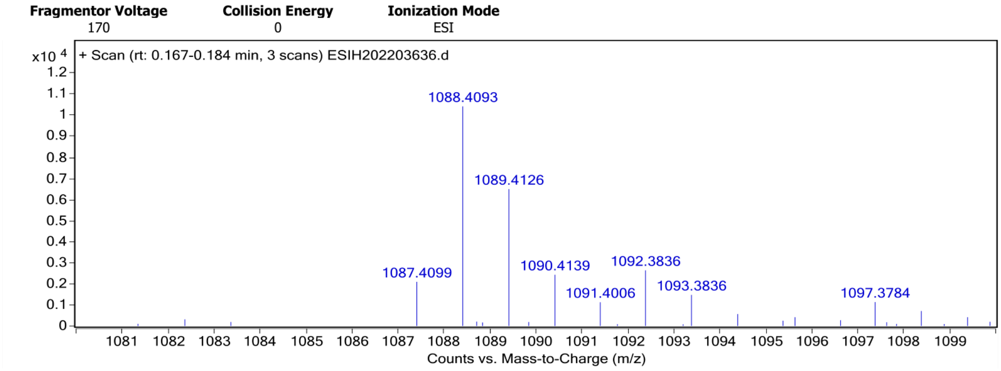

Formula Calculator Results

m/z	Calc m/z	Diff (mDa)	Diff (ppm)	Ion Formula	Ion
1088.4093	1088.4082	-1.14	-1.04	C58 H63 [11B] N O19	(M+H)+

Figure S8. HR-MS spectra of compound 4a

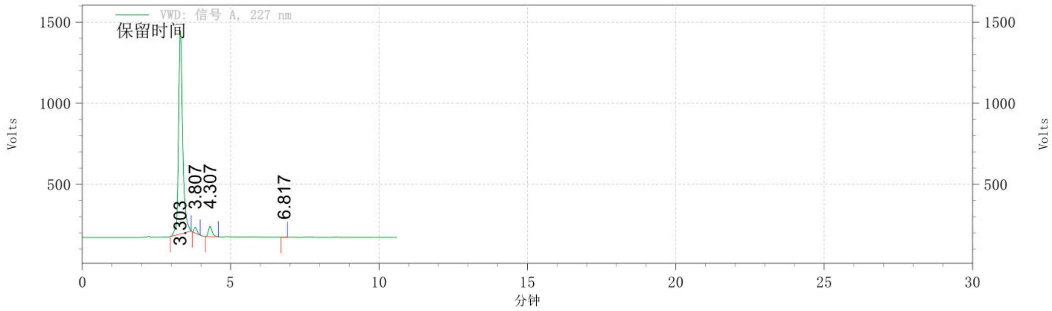

VWD: 信号 A,
227 nm 结果

保留时间	面积	面积百分比	峰高	峰高百分比
3.303	202850228	93.70	21013610	92.67
3.807	4332322	2.00	606503	2.67
4.307	9274418	4.28	1051798	4.64
6.817	23580	0.01	3011	0.01

Figure S9. HPLC chromatograms of compound 4a

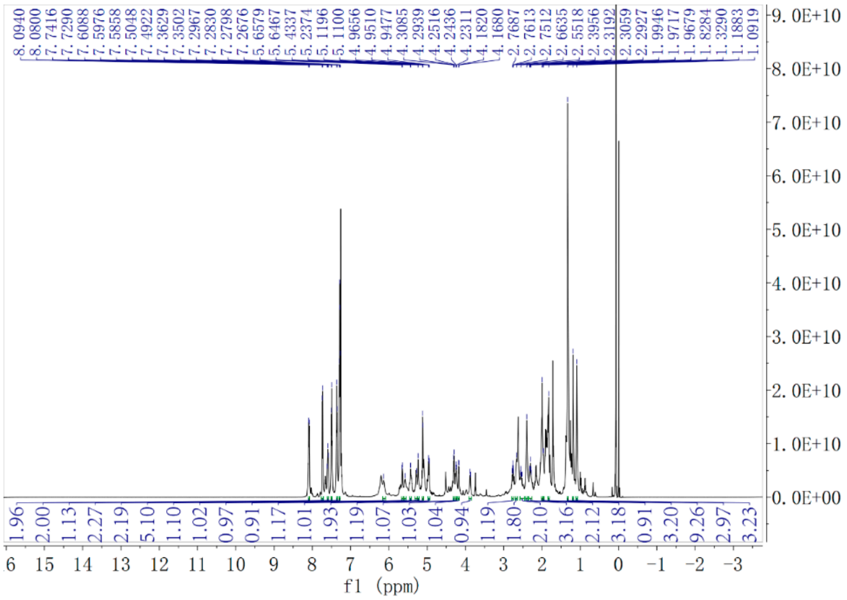

Figure S10. ^{1}H-NMR spectra of compound 4b

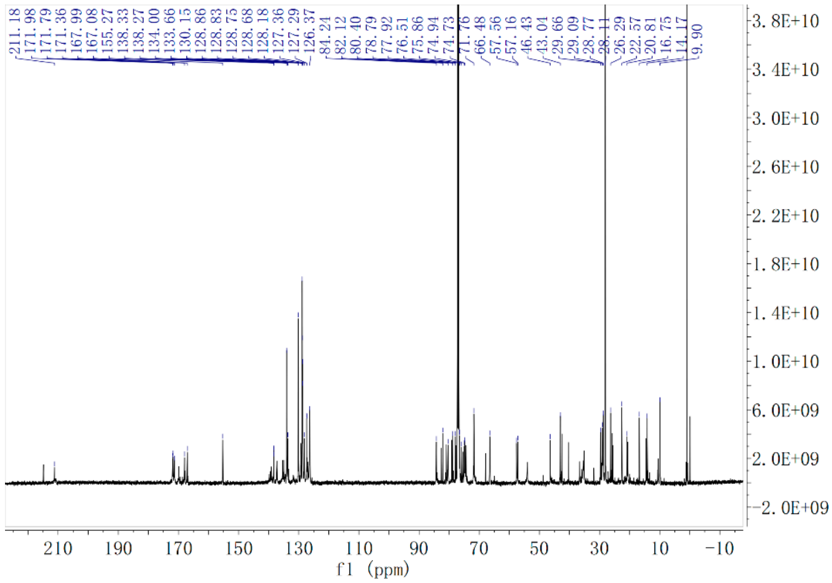

Figure S11. ^{13}C-NMR spectra of compound 4b

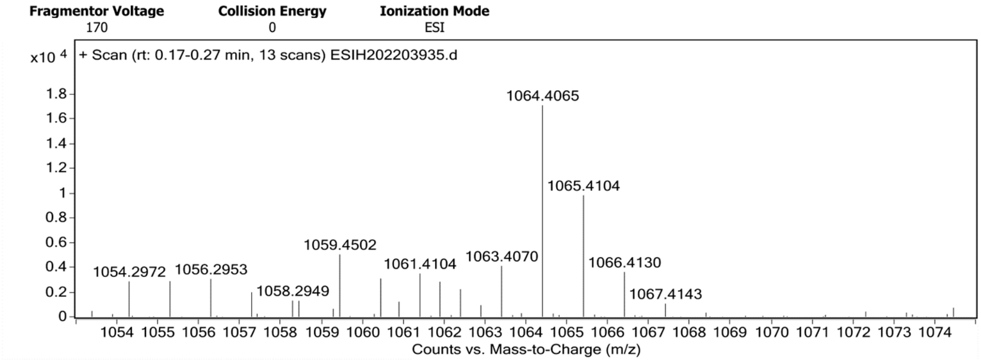

Formula Calculator Results

m/z	Calc m/z	Diff (mDa)	Diff (ppm)	Ion Formula	Ion
1064.4065	1064.4058	-0.77	-0.72	C54 H64 [11B] N Na O19	(M+Na)+

Figure S12. HR-MS spectra of compound 4b

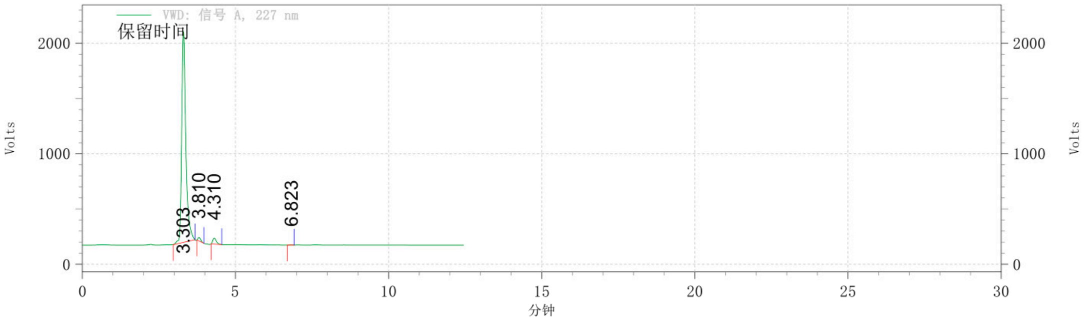

VWD: 信号 A,
227 nm 结果

保留时间	面积	面积百分比	峰高	峰高百分比
3.303	301689476	96.56	32014628	95.72
3.810	3387846	1.08	527497	1.58
4.310	7341443	2.35	901936	2.70
6.823	16648	0.01	2122	0.01

Figure S13. HPLC chromatograms of compound 4b

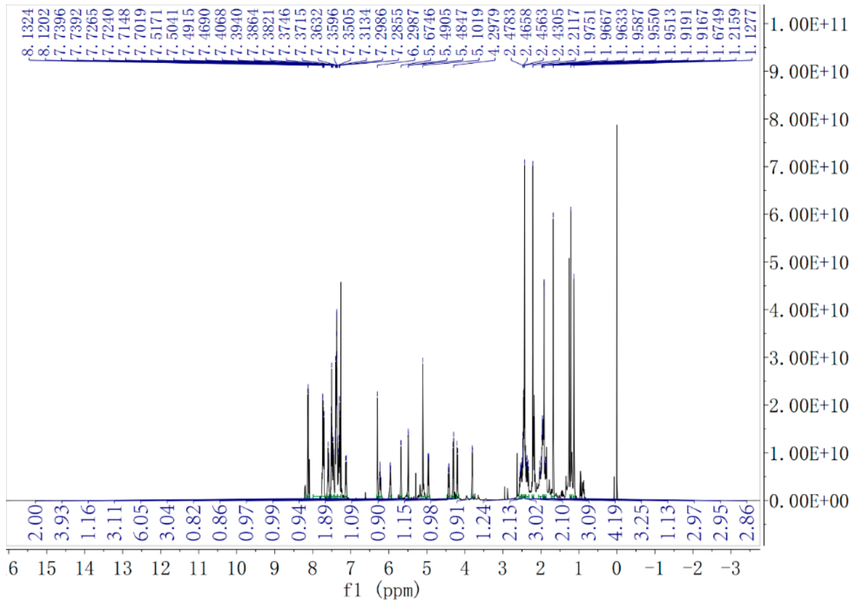

Figure S14. ^{1}H-NMR spectra of compound 4c

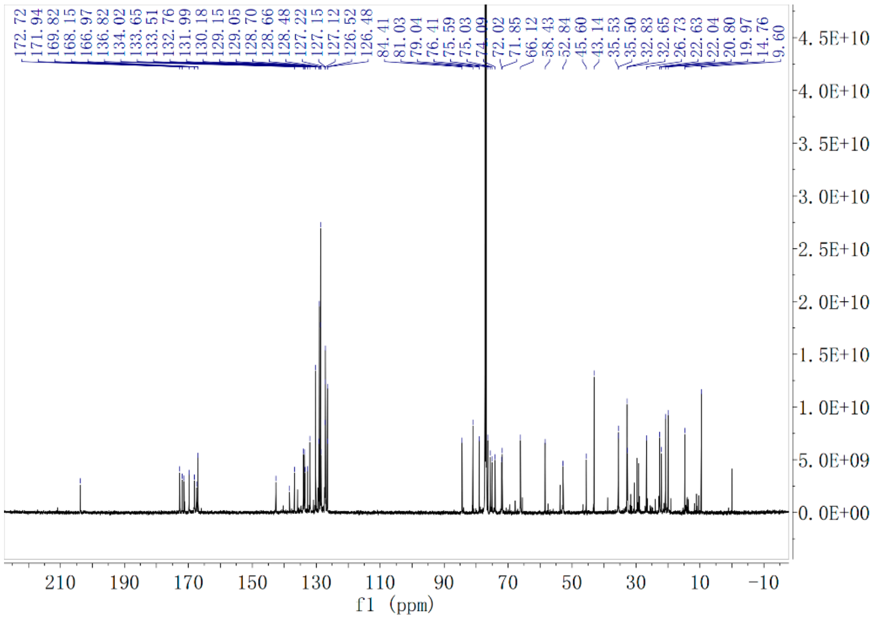

Figure S15. ^{13}C-NMR spectra of compound 4c

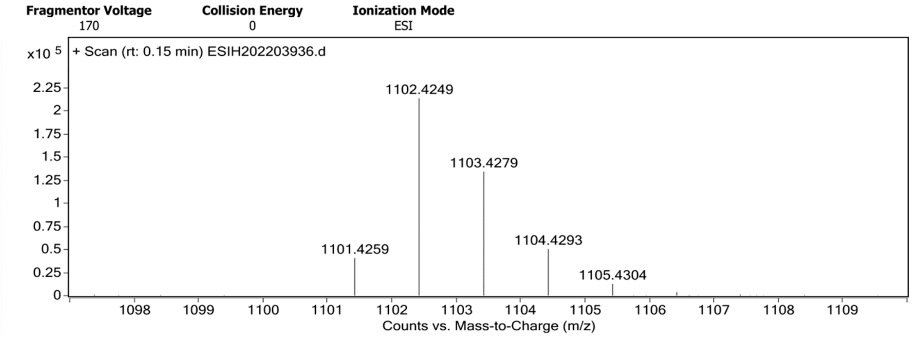

Formula Calculator Results

m/z	Calc m/z	Diff (mDa)	Diff (ppm)	Ion Formula	Ion
1102.4249	1102.4238	-1.11	-1.01	C59 H65 [11B] N O19	(M+H)+

Figure S16. HR-MS spectra of compound 4c

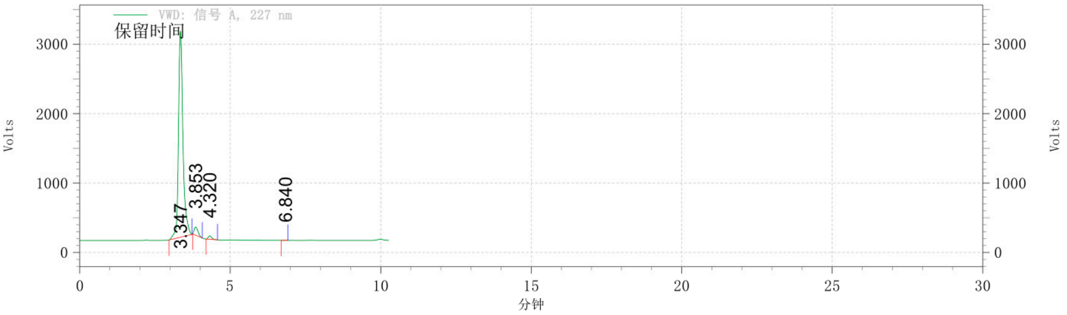

VWD: 信号 A,
227 nm 结果

保留时间	面积	面积百分比	峰高	峰高百分比
3.347	516357052	95.51	49816412	94.75
3.853	17584581	3.25	1946608	3.70
4.320	6676862	1.24	811820	1.54
6.840	15527	0.00	2121	0.00

Figure S17. HPLC chromatograms of compound 4c

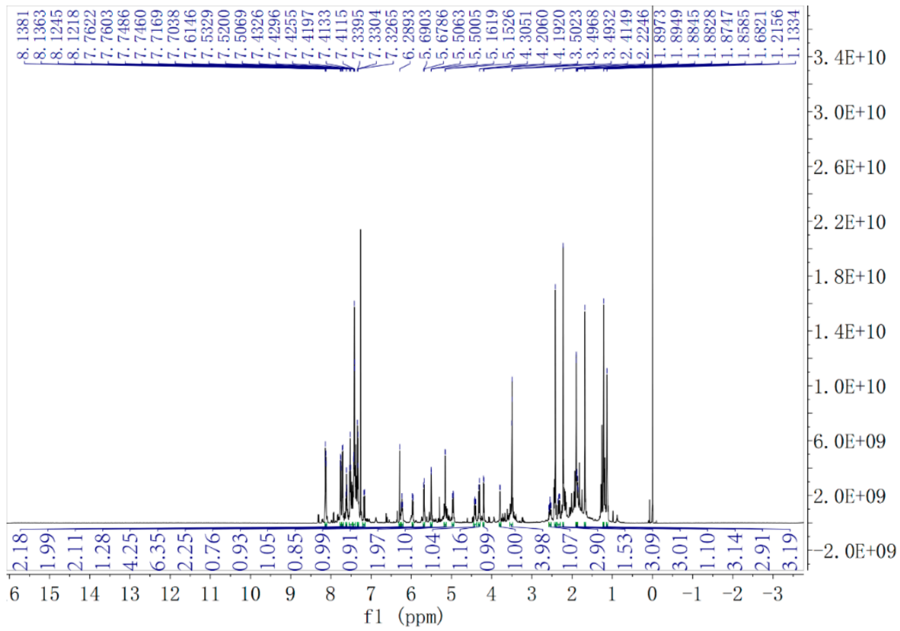

Figure S18. ^{1}H-NMR spectra of compound 4d

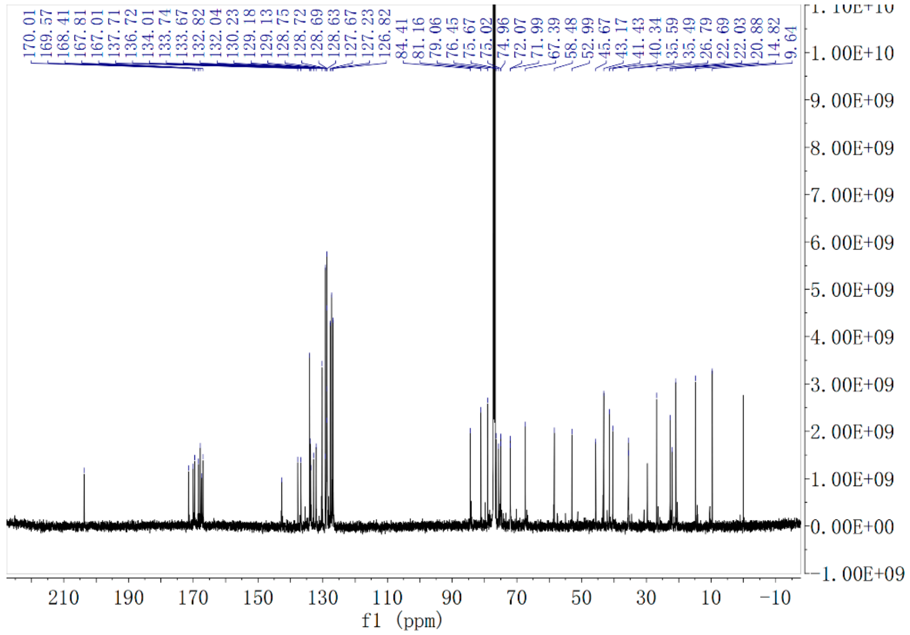

Figure S19. ^{13}C-NMR spectra of compound 4d

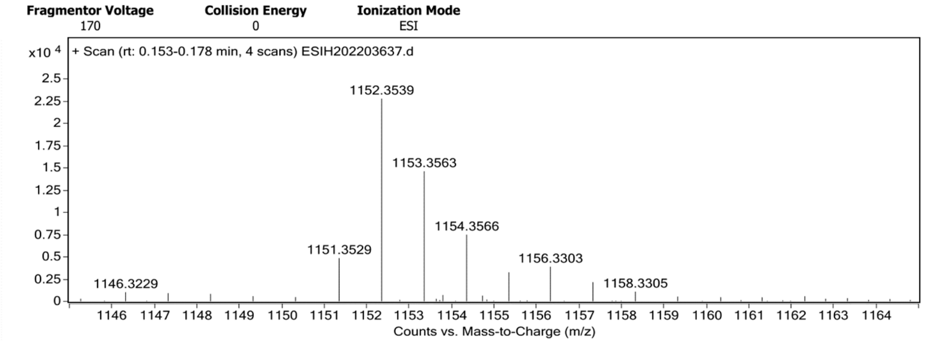

Formula Calculator Results

m/z	Calc m/z	Diff (mDa)	Diff (ppm)	Ion Formula	Ion
1152.3539	1152.3523	-1.52	-1.32	C58 H63 [11B] N O19 S2	(M+H)+

Figure S20. HR-MS spectra of compound 4d

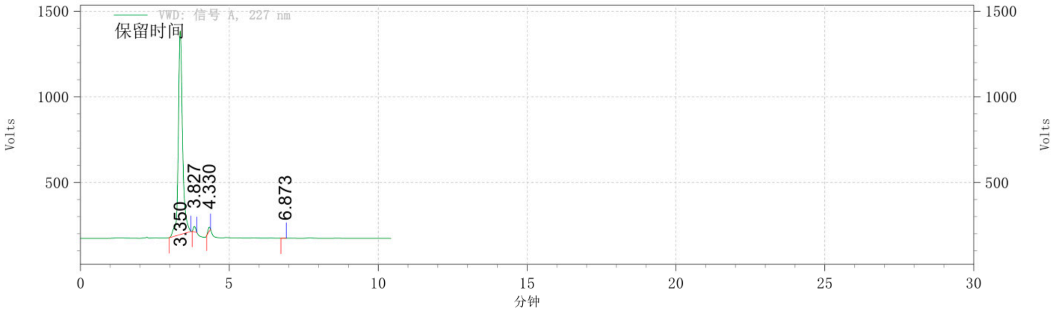

VWD: 信号 A,
227 nm 结果

保留时间	面积	面积百分比	峰高	峰高百分比
3.350	196844443	97.58	19958088	95.55
3.827	2947768	1.46	528704	2.53
4.330	1929437	0.96	399810	1.91
6.873	7430	0.00	1028	0.00

Figure S21. HPLC chromatograms of compound 4d

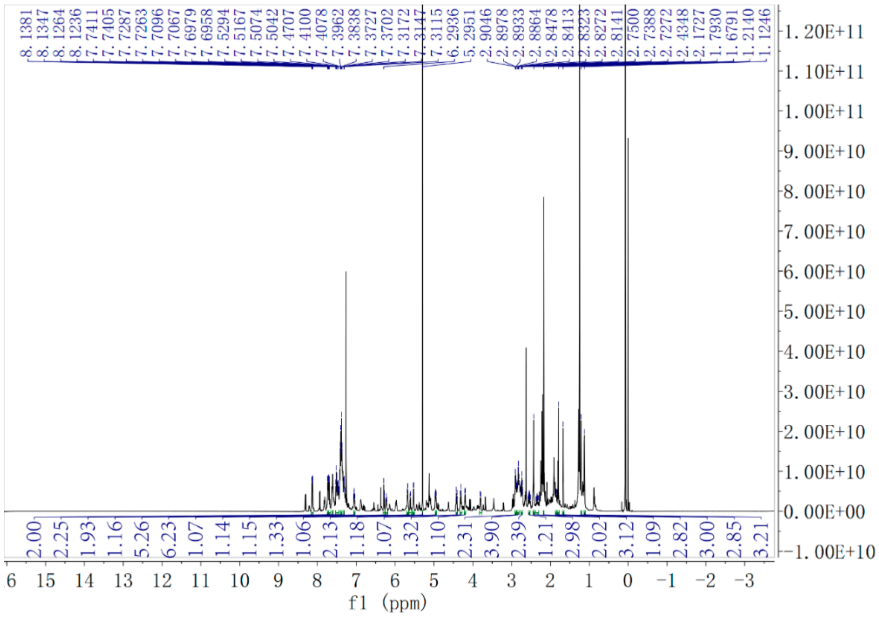

Figure S22. ^{1}H-NMR spectra of compound 4e

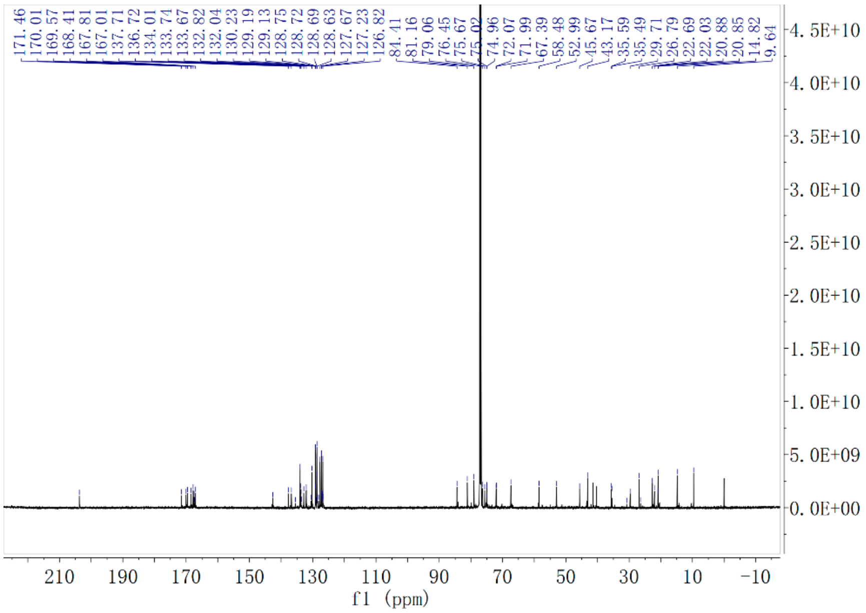

Figure S23. ^{13}C-NMR spectra of compound 4e

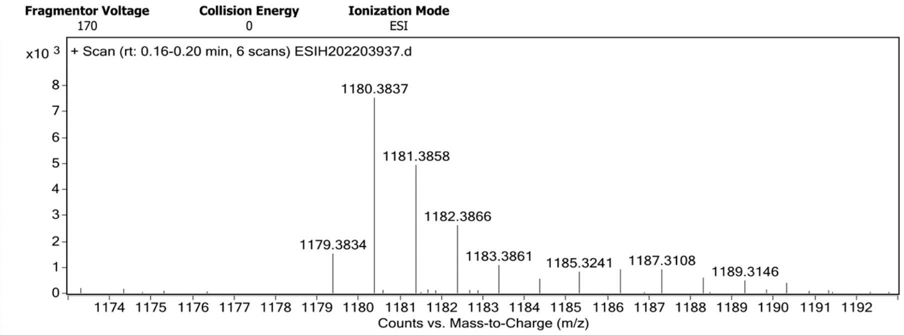

Formula Calculator Results

m/z	Calc m/z	Diff (mDa)	Diff (ppm)	Ion Formula	Ion
1180.3837	1180.3836	-0.09	-0.08	C60 H67 [11B] N O19 S2	(M+H)+

Figure S24. HR-MS spectra of compound 4e

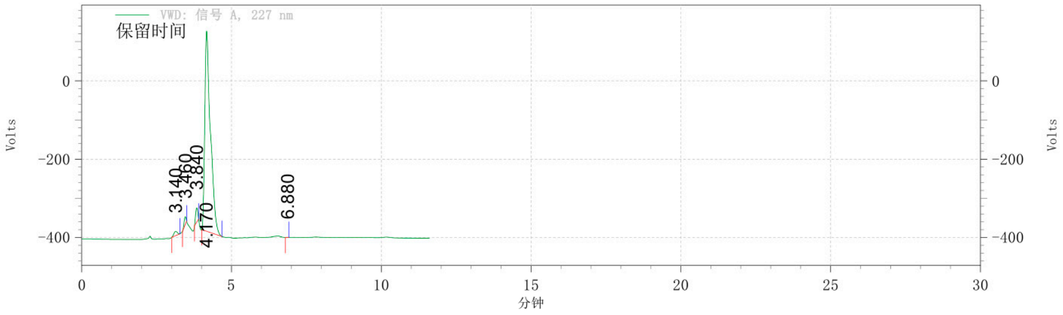

VWD: 信号 A,
227 nm 结果

保留时间	面积	面积百分比	峰高	峰高百分比
3.140	1460606	1.24	179465	1.85
3.460	1465230	1.24	321094	3.31
3.840	3017699	2.55	610289	6.30
4.170	112237745	94.97	8579314	88.53
6.880	2690	0.00	594	0.01

Figure S25. HPLC chromatograms of compound 4e

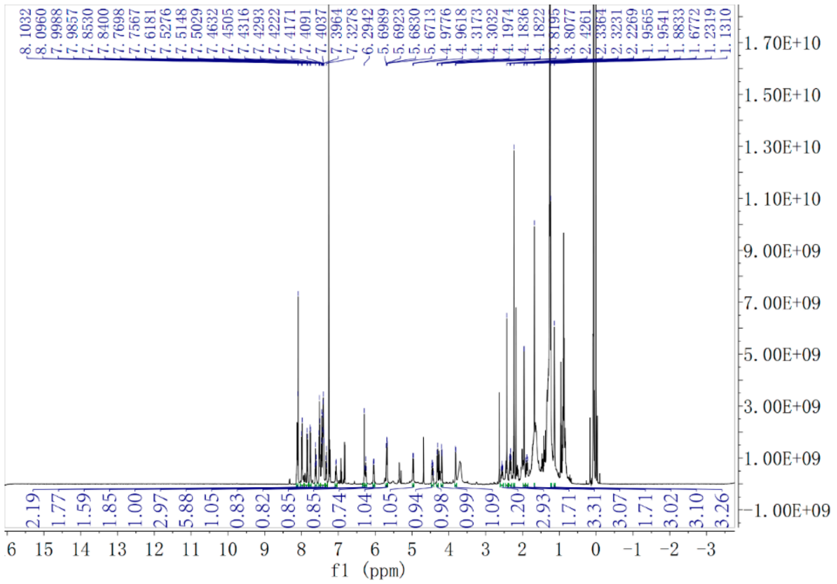

Figure S26. ^{1}H-NMR spectra of compound 6a

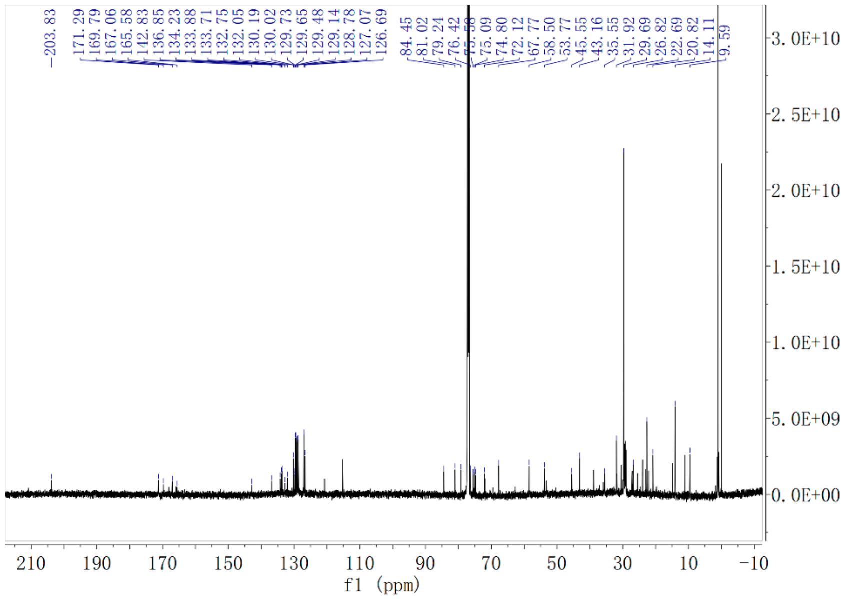

Figure S27. ^{13}C-NMR spectra of compound 6a

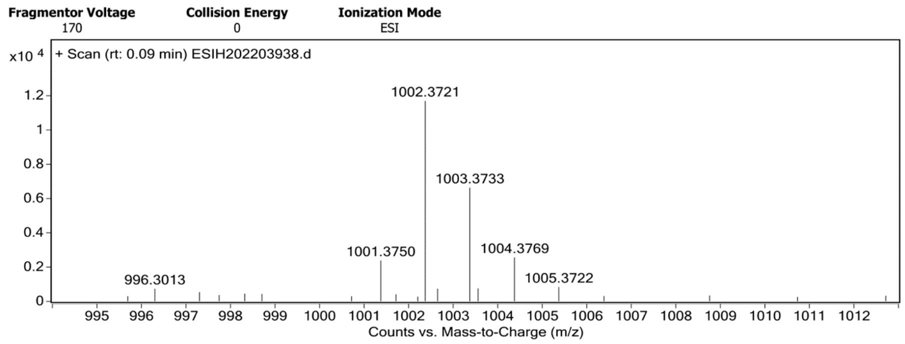

Formula Calculator Results

m/z	Calc m/z	Diff (mDa)	Diff (ppm)	Ion Formula	Ion
1002.3721	1002.3714	-0.67	-0.66	C54 H57 [11B] N O17	(M+H)+

Figure S28. HR-MS spectra of compound 6a

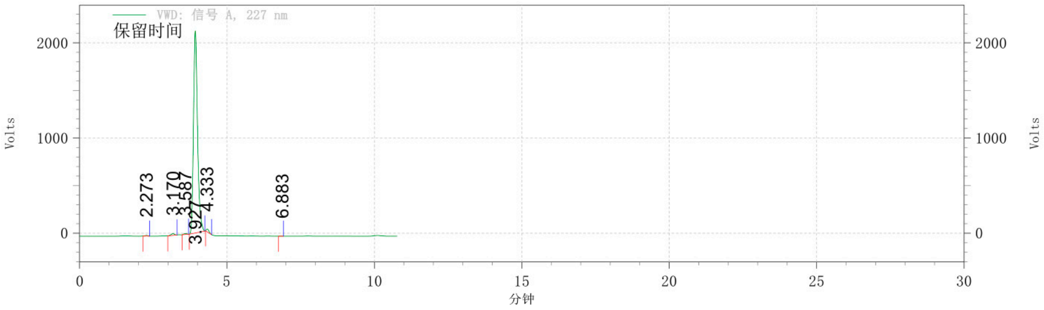

VWD: 信号 A,
227 nm 结果

保留时间	面积	面积百分比	峰高	峰高百分比
2.273	680939	0.20	145230	0.40
3.170	2510468	0.73	296388	0.81
3.587	1138676	0.33	159563	0.43
3.927	338468078	97.86	35635624	97.01
4.333	3056780	0.88	496256	1.35
6.883	5349	0.00	782	0.00

Figure S29. HPLC chromatograms of compound 6a

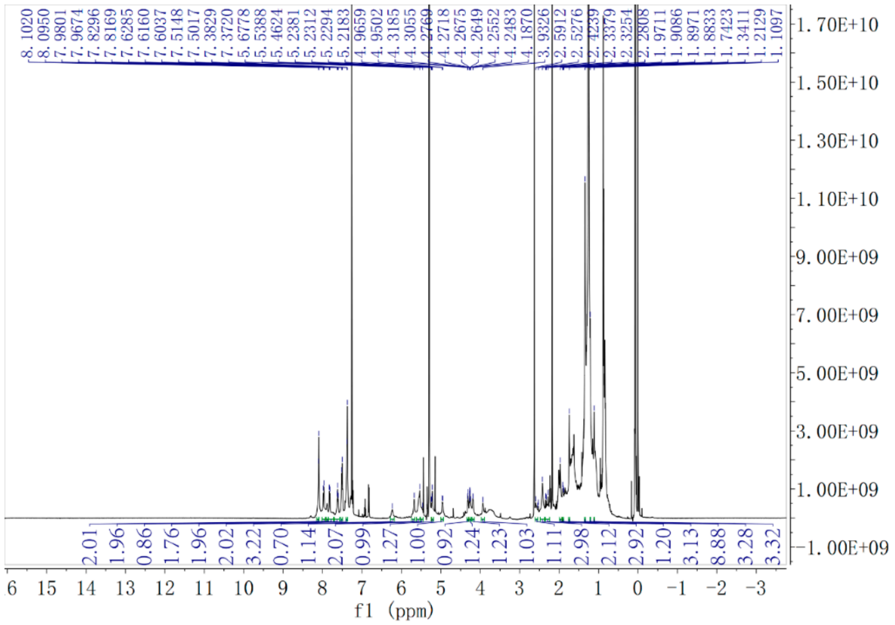

Figure S30. ^{1}H-NMR spectra of compound 6b

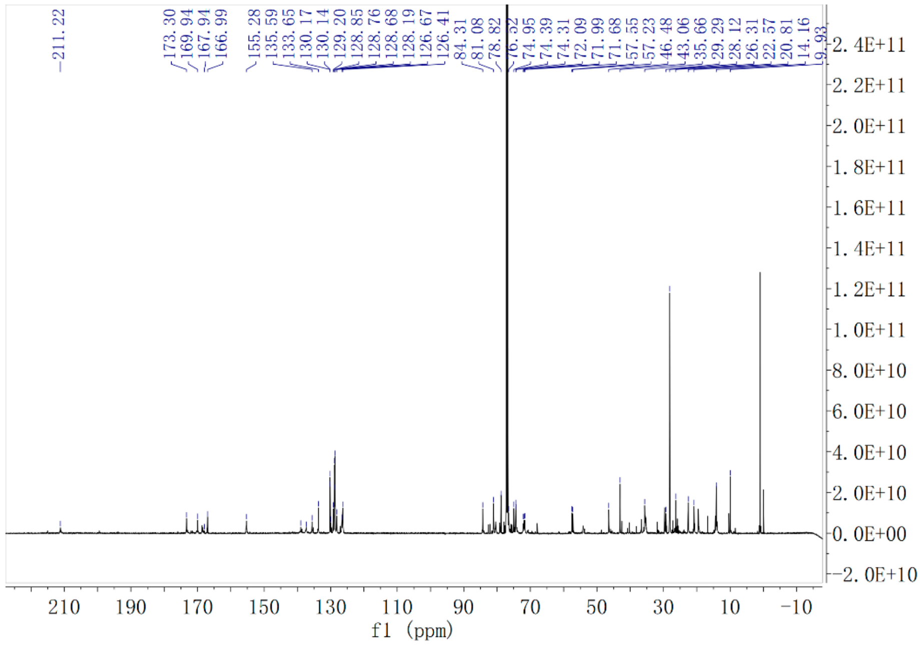

Figure S31. ^{13}C-NMR spectra of compound 6b

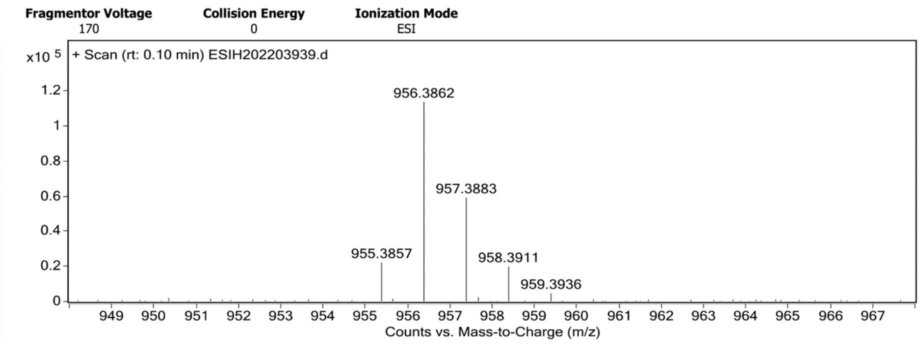

Formula Calculator Results

m/z	Calc m/z	Diff (mDa)	Diff (ppm)	Ion Formula	Ion
956.3862	956.3871	0.83	0.86	C50 H59 [11B] N O17	(M+H)+

Figure S32. HR-MS spectra of compound 6b

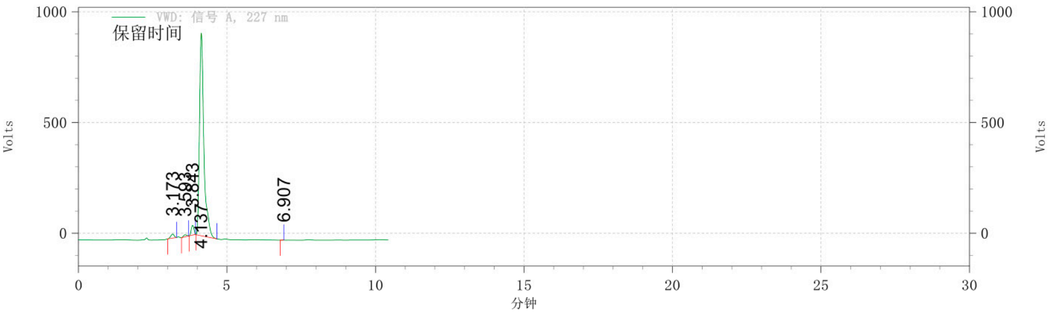

VWD: 信号 A,
227 nm 结果

保留时间	面积	面积百分比	峰高	峰高百分比
3.173	2447688	1.47	303910	1.84
3.593	1110912	0.67	149723	0.91
3.843	4681360	2.82	729642	4.41
4.137	157908940	95.04	15349572	92.84
6.907	1681	0.00	131	0.00

Figure S33. HPLC chromatograms of compound 6b

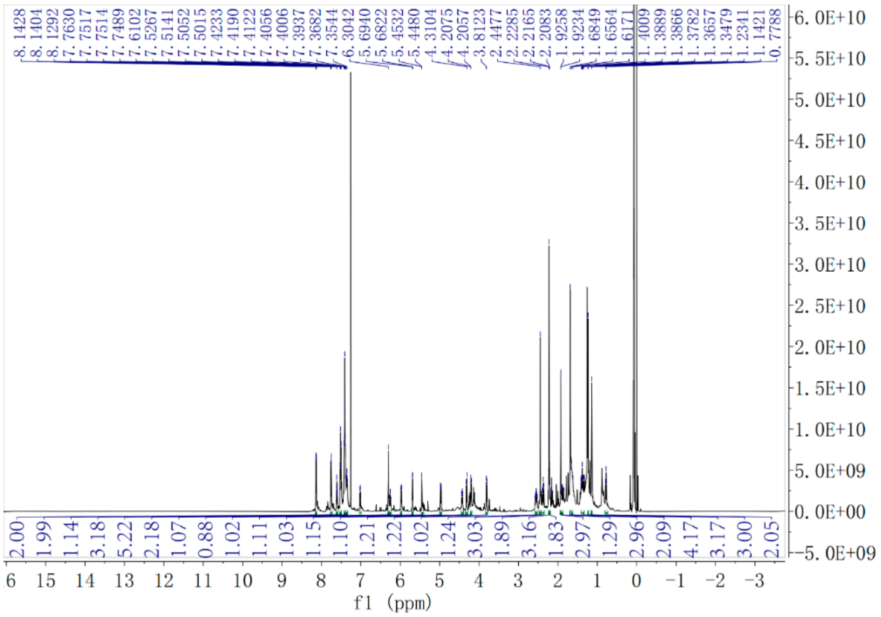

Figure S34. ^{1}H-NMR spectra of compound 10a

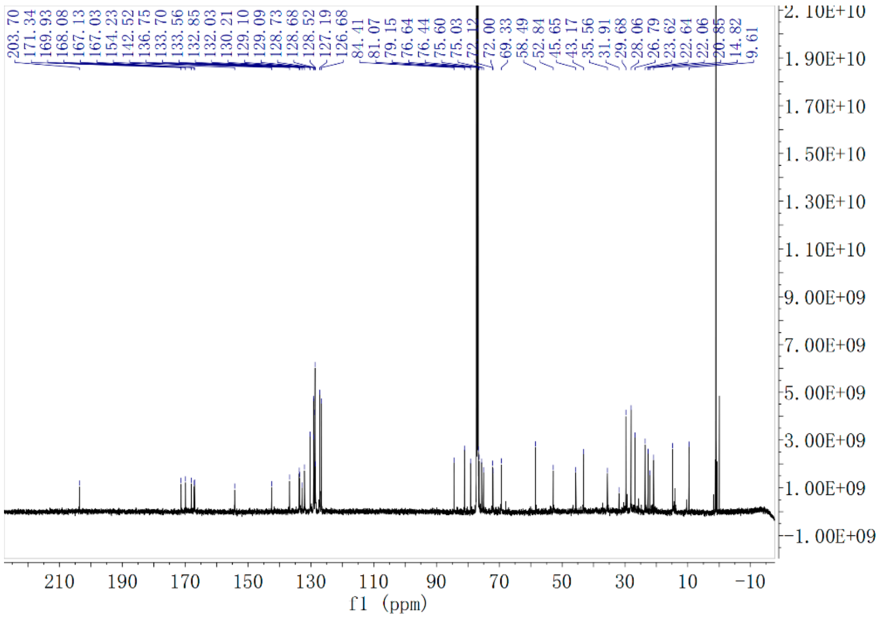

Figure S35. ^{13}C-NMR spectra of compound 10a

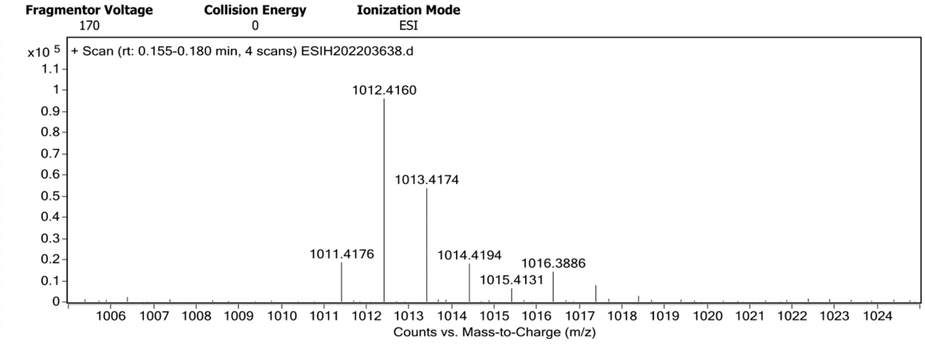

Formula Calculator Results

m/z	Calc m/z	Diff (mDa)	Diff (ppm)	Ion Formula	Ion
1012.416	1012.4133	-2.71	-2.68	C53 H63 [11B] N O18	(M+H)+

Figure S36. HR-MS spectra of compound 10a

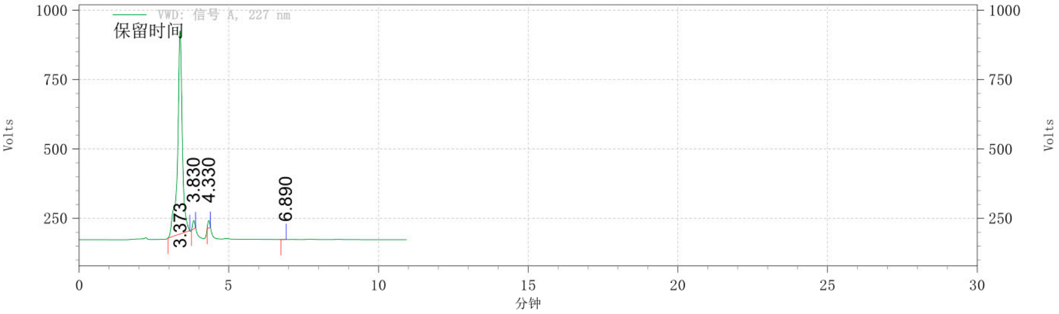

VWD: 信号 A,
227 nm 结果

保留时间	面积	面积百分比	峰高	峰高百分比
3.373	135097510	96.84	12276348	92.86
3.830	2511602	1.80	501128	3.79
4.330	1885796	1.35	442313	3.35
6.890	5270	0.00	653	0.00

Figure S37. HPLC chromatograms of compound 10a

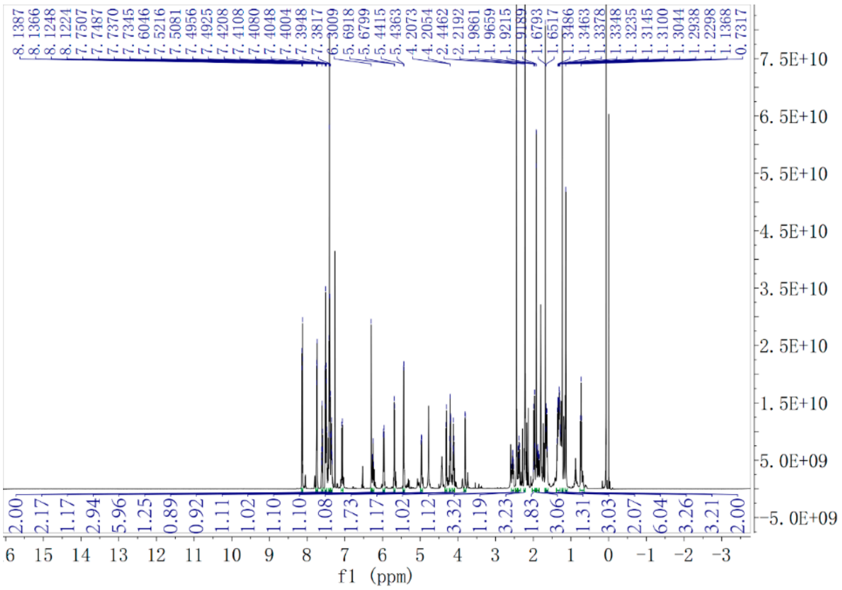

Figure S38. ^{1}H-NMR spectra of compound 10b

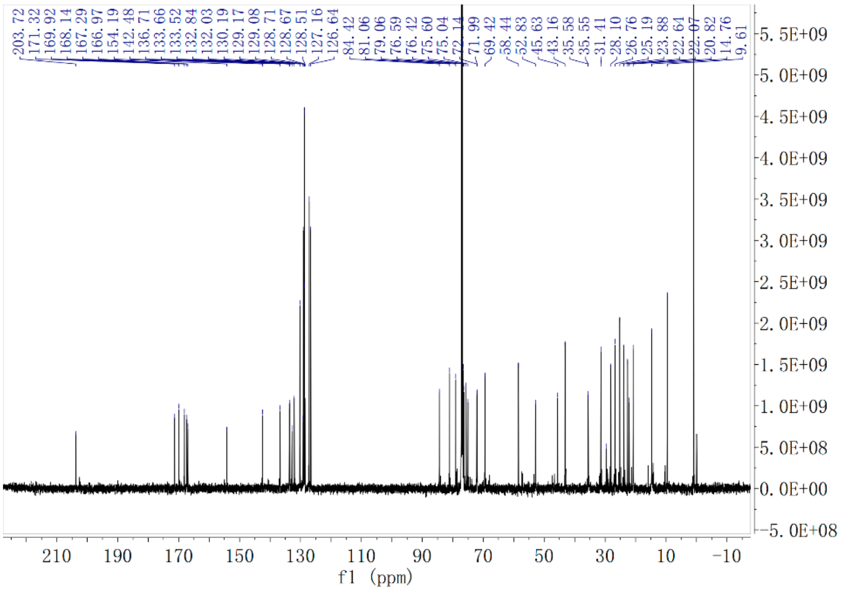

Figure S39. ^{13}C-NMR spectra of compound 10b

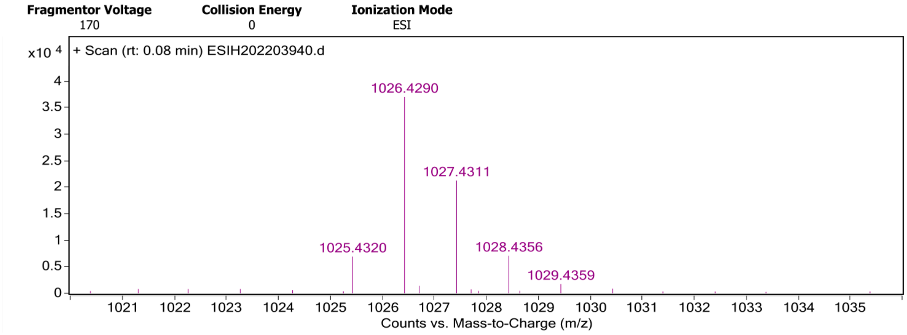

Formula Calculator Results

m/z	Calc m/z	Diff (mDa)	Diff (ppm)	Ion Formula	Ion
1026.429	1026.4289	-0.07	-0.07	C54 H65 [11B] N O18	(M+H)+

Figure S40. HR-MS spectra of compound 10b

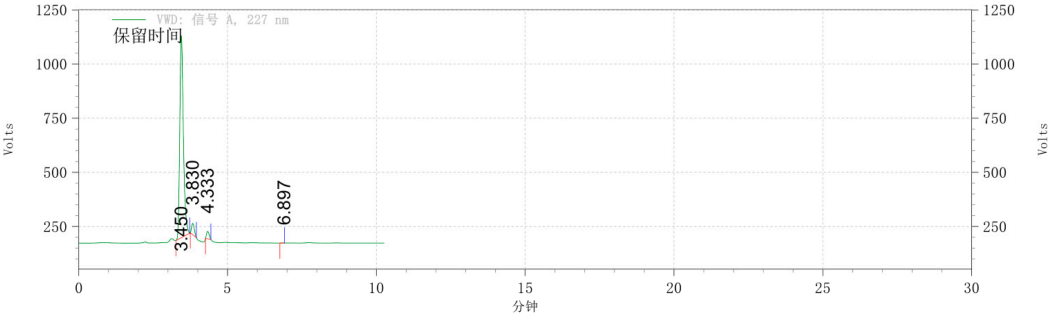

VWD: 信号 A,
227 nm 结果

保留时间	面积	面积百分比	峰高	峰高百分比
3.450	132217731	93.52	15658069	91.52
3.830	5631761	3.98	876780	5.12
4.333	3521082	2.49	574368	3.36
6.897	4433	0.00	488	0.00

Figure S41. HPLC chromatograms of compound 10b

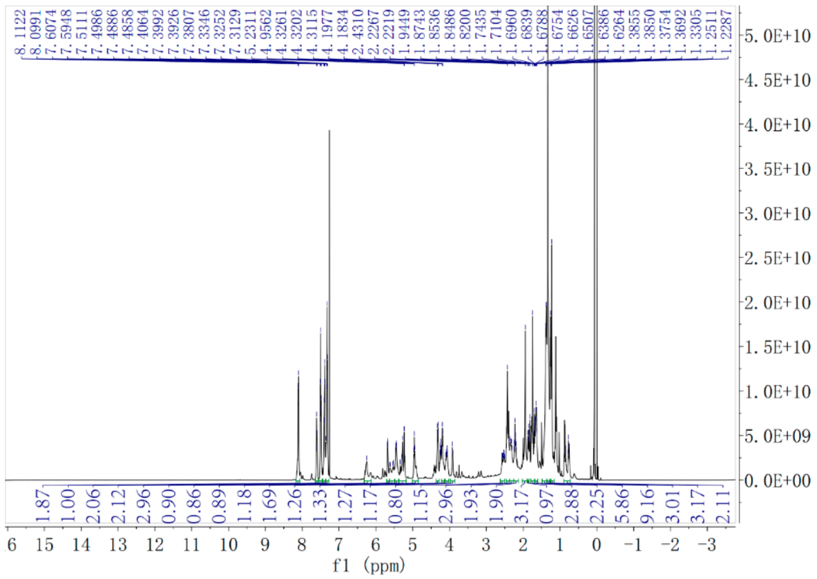

Figure S42. ^{1}H-NMR spectra of compound 10c

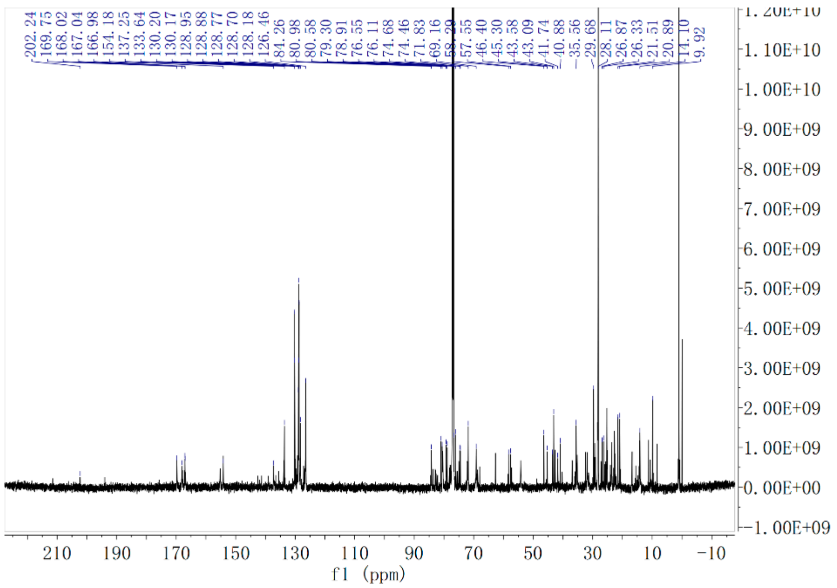

Figure S43. ^{13}C-NMR spectra of compound 10c

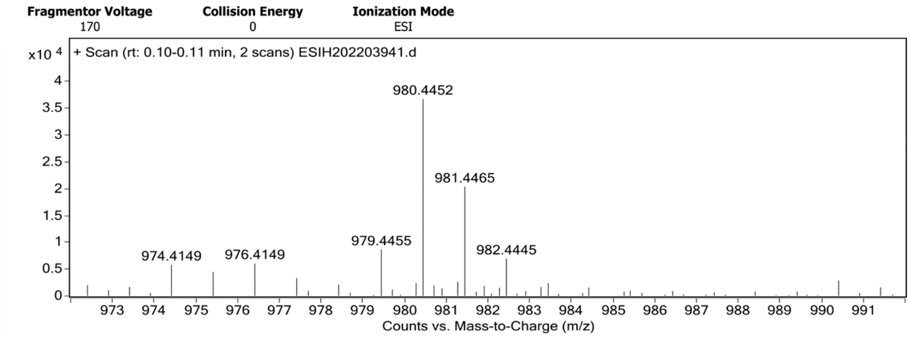

Formula Calculator Results

m/z	Calc m/z	Diff (mDa)	Diff (ppm)	Ion Formula	Ion
980.4452	980.4446	-0.6	-0.61	C50 H67 [11B] N O18	(M+H)+

Figure S44. HR-MS spectra of compound 10c

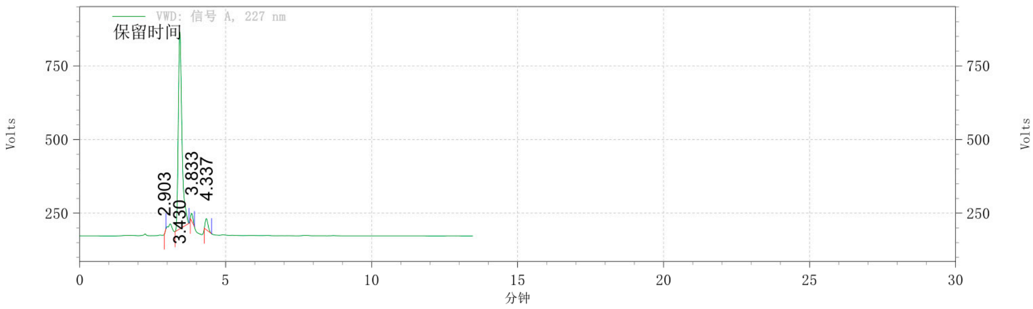

VWD: 信号 A,
227 nm 结果

保留时间	面积	面积百分比	峰高	峰高百分比
2.903	35410	0.03	0	0.00
3.430	97838091	93.86	11208798	91.38
3.833	2195015	2.11	429144	3.50
4.337	4164751	4.00	628861	5.13

Figure S45. HPLC chromatograms of compound 10c

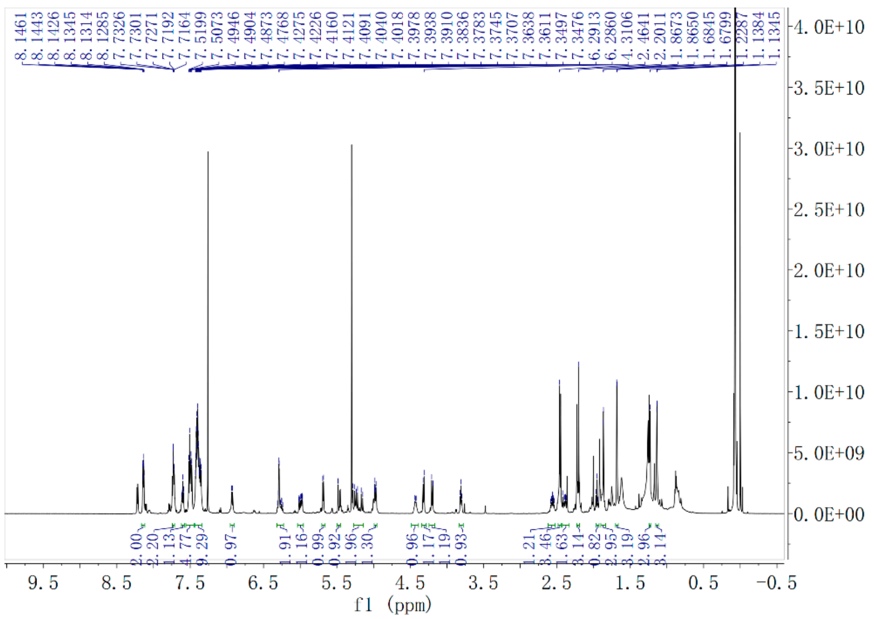

Figure S46. ^{1}H-NMR spectra of compound 13a

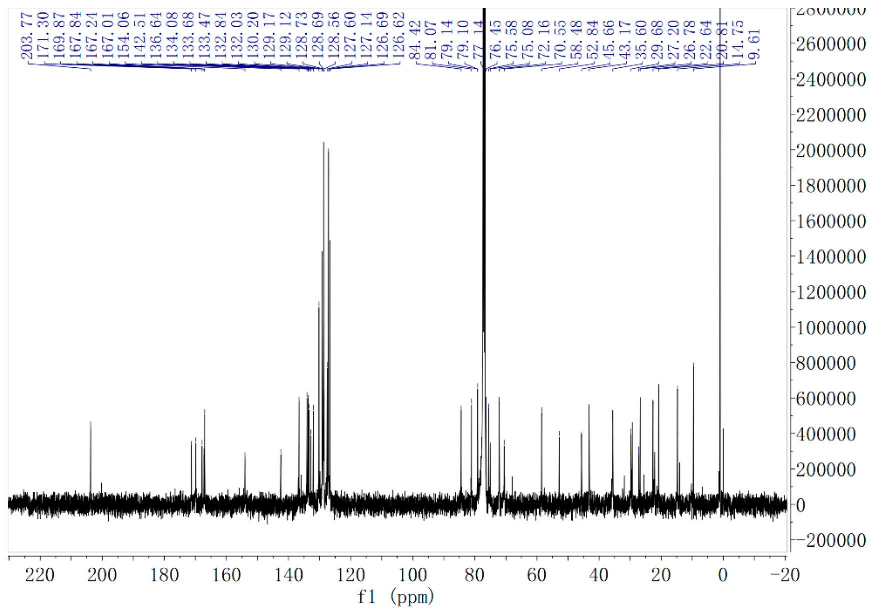

Figure S47. ^{13}C-NMR spectra of compound 13a

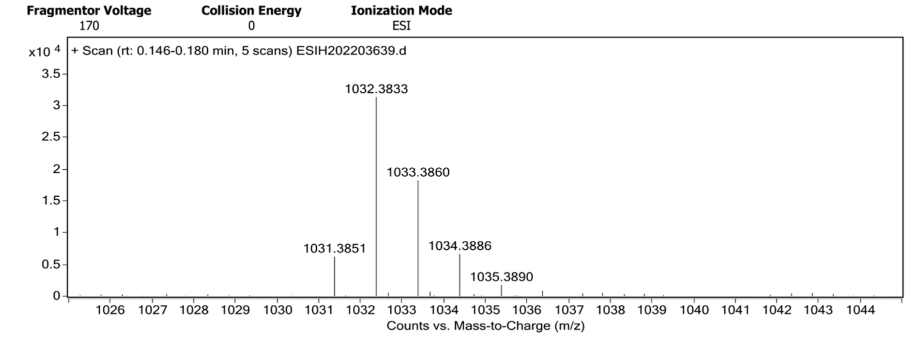

Formula Calculator Results

m/z	Calc m/z	Diff (mDa)	Diff (ppm)	Ion Formula	Ion
1032.3833	1032.382	-1.32	-1.28	C55 H59 [11B] N O18	(M+H)+

Figure S48. HR-MS spectra of compound 13a

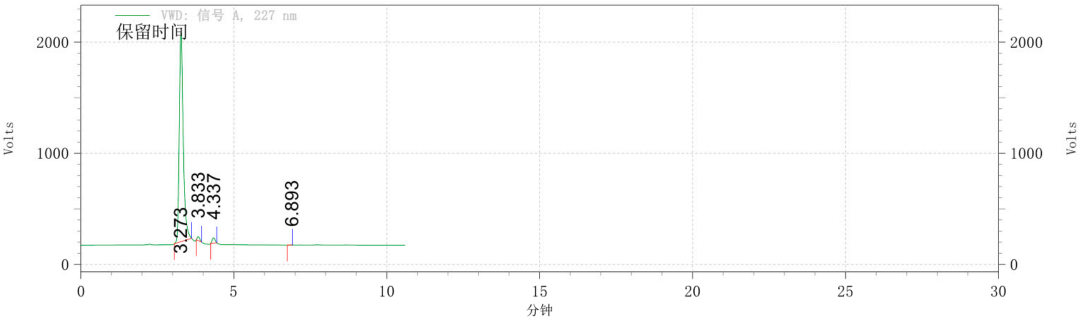

VWD: 信号 A,
227 nm 结果

保留时间	面积	面积百分比	峰高	峰高百分比
3.273	286625732	97.07	31681539	95.84
3.833	3412625	1.16	587542	1.78
4.337	5228631	1.77	788544	2.39
6.893	5550	0.00	558	0.00

Figure S49. HPLC chromatograms of compound 13a

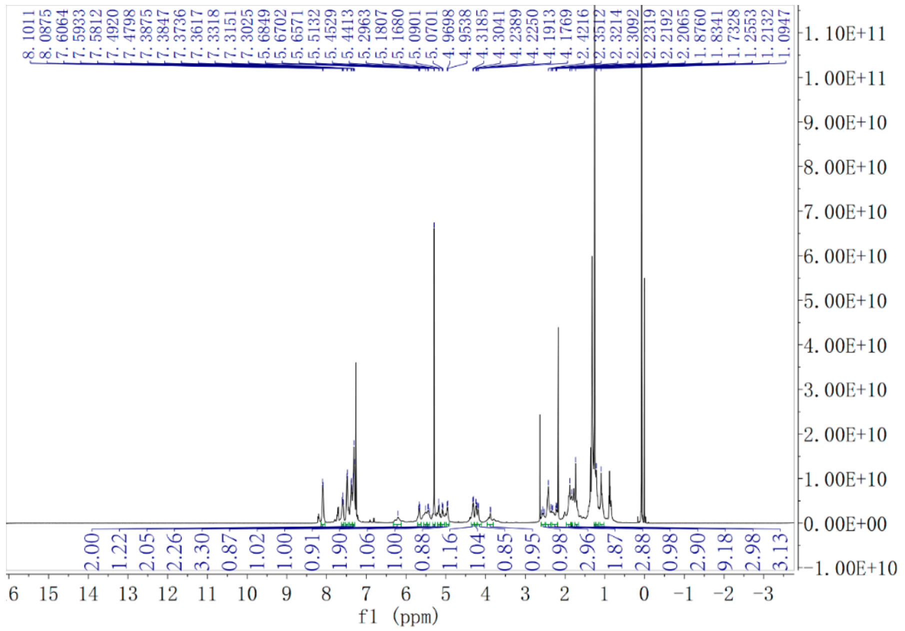

Figure S50. ^{1}H-NMR spectra of compound 13b

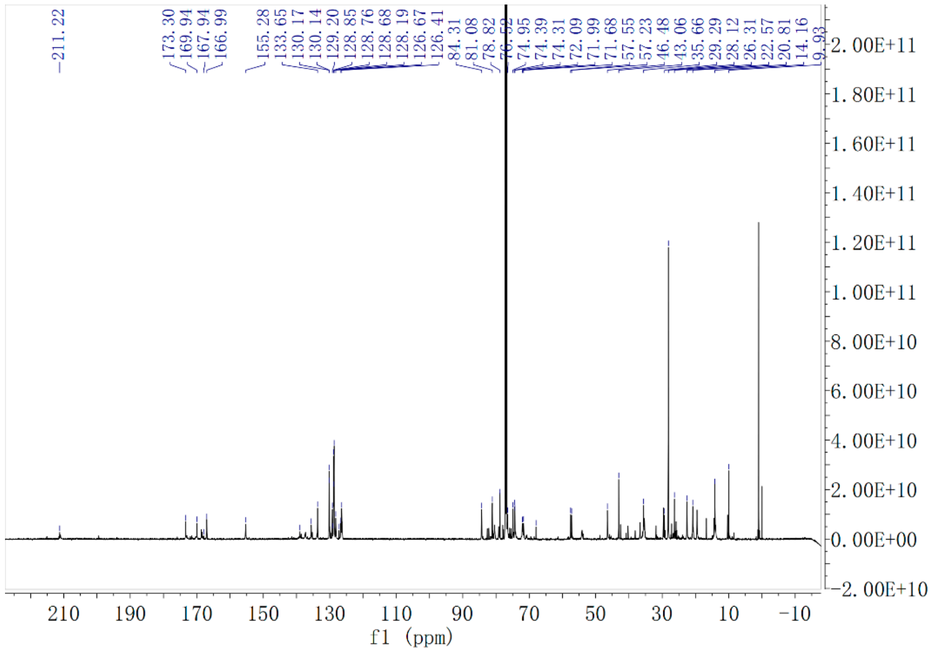

Figure S51. ^{13}C-NMR spectra of compound 13b

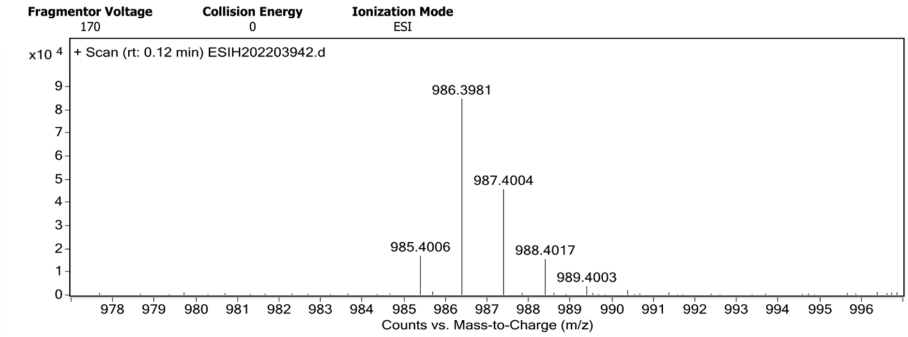

Formula Calculator Results

m/z	Calc m/z	Diff (mDa)	Diff (ppm)	Ion Formula	Ion
986.3981	986.3976	-0.45	-0.46	C51 H61 [11B] N O18	(M+H)+

Figure S52. HR-MS spectra of compound 13b

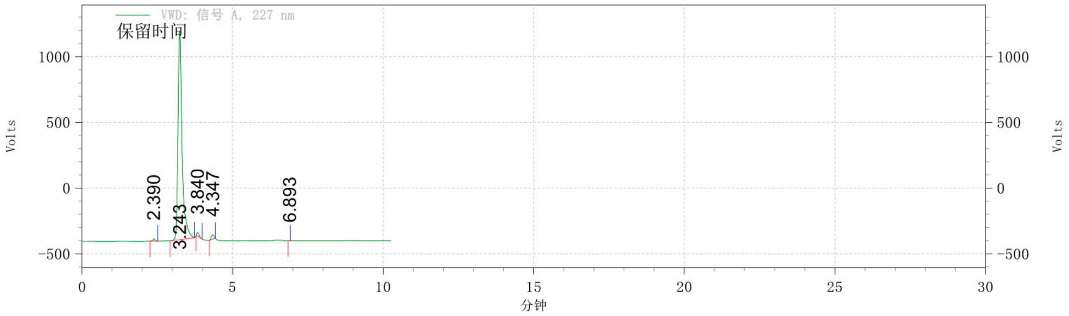

VWD: 信号 A,
227 nm 结果

保留时间	面积	面积百分比	峰高	峰高百分比
2.390	1209374	0.47	304538	1.09
3.243	252045449	97.25	26616135	95.37
3.840	2535035	0.98	453968	1.63
4.347	3375957	1.30	532825	1.91
6.893	375	0.00	121	0.00

Figure S53. HPLC chromatograms of compound 13b

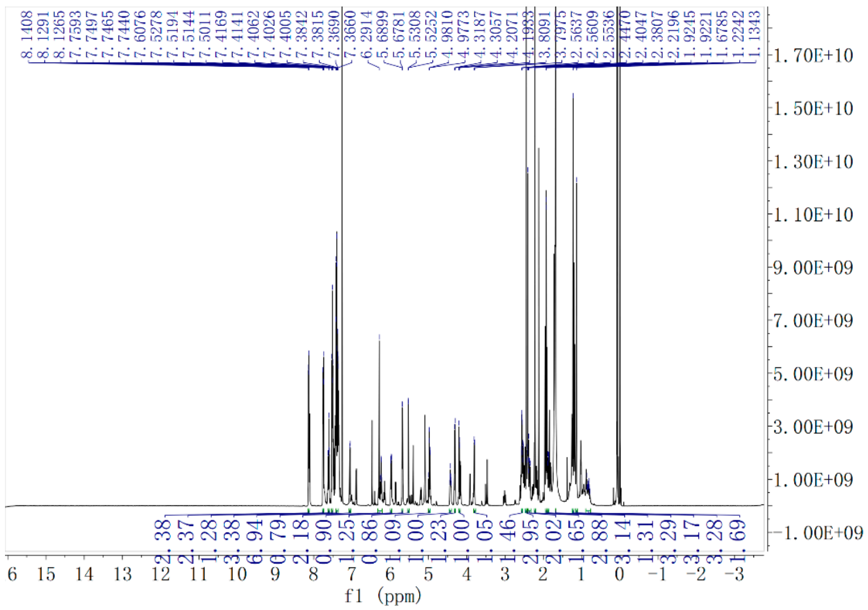

Figure S54. ^{1}H-NMR spectra of compound 17a

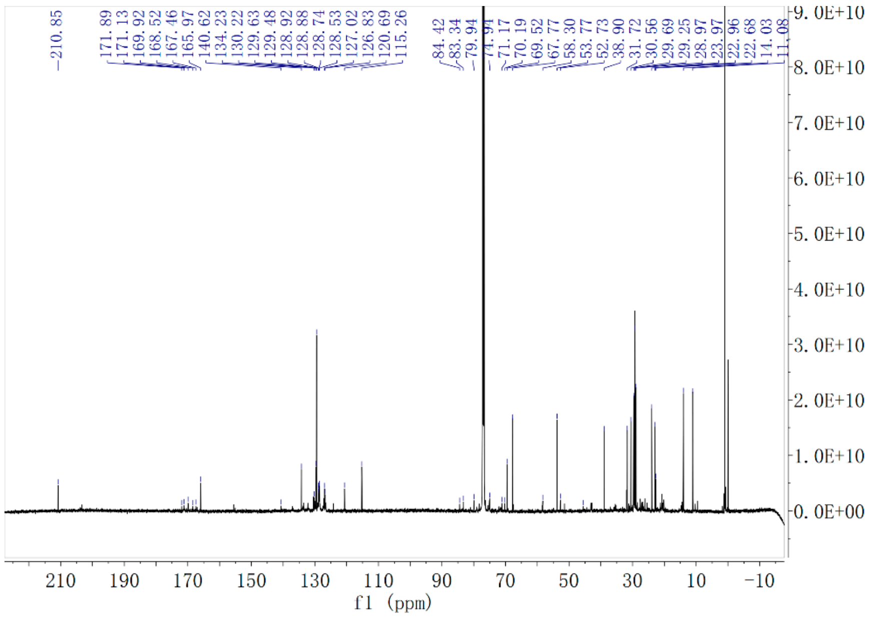

Figure S55. ^{13}C-NMR spectra of compound 17a

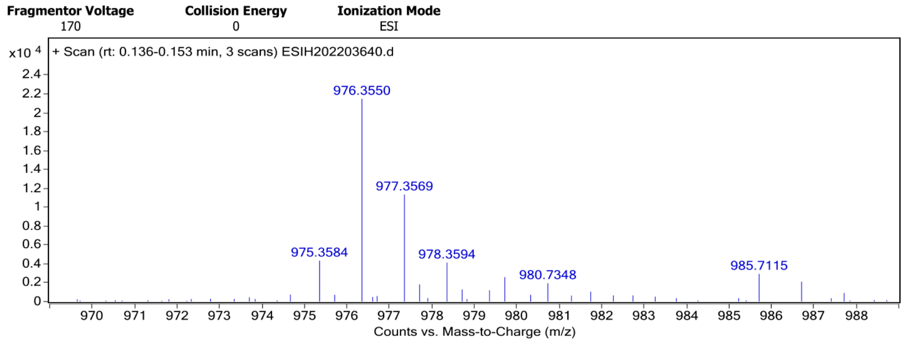

Formula Calculator Results

m/z	Calc m/z	Diff (mDa)	Diff (ppm)	Ion Formula	Ion
976.355	976.3534	-1.67	-1.71	C50 H56 [11B] N Na O17	(M+Na)+

Figure S56. HR-MS spectra of compound 17a

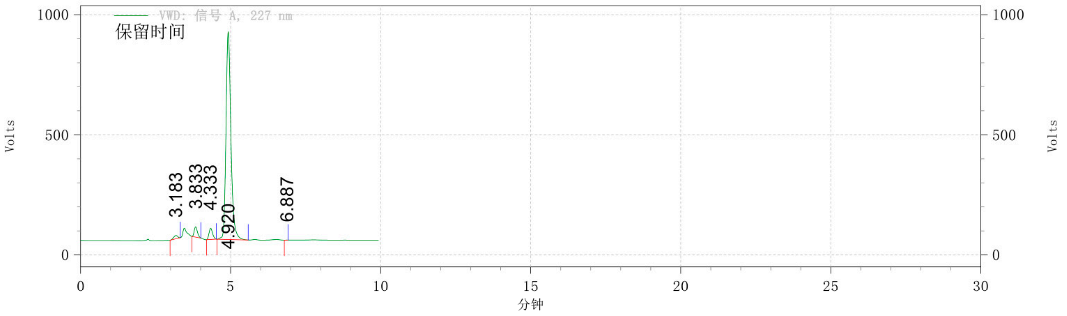

VWD: 信号 A,
227 nm 结果

保留时间	面积	面积百分比	峰高	峰高百分比
3.183	2090616	1.17	223368	1.38
3.833	5365137	3.01	716418	4.42
4.333	6426604	3.60	778456	4.80
4.920	164567361	92.22	14507453	89.41
6.887	2263	0.00	423	0.00

Figure S57. HPLC chromatograms of compound 17a

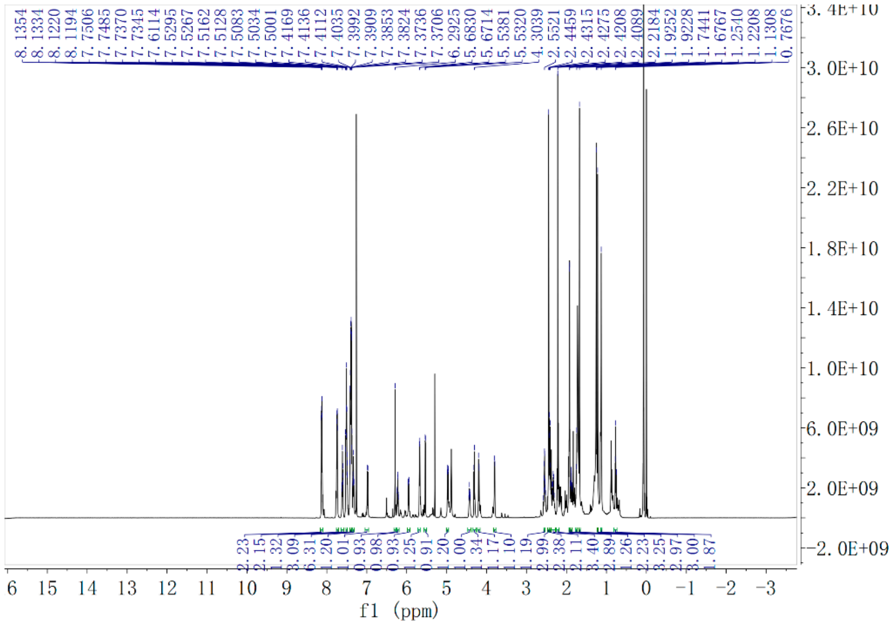

Figure S58. ^{1}H-NMR spectra of compound 17b

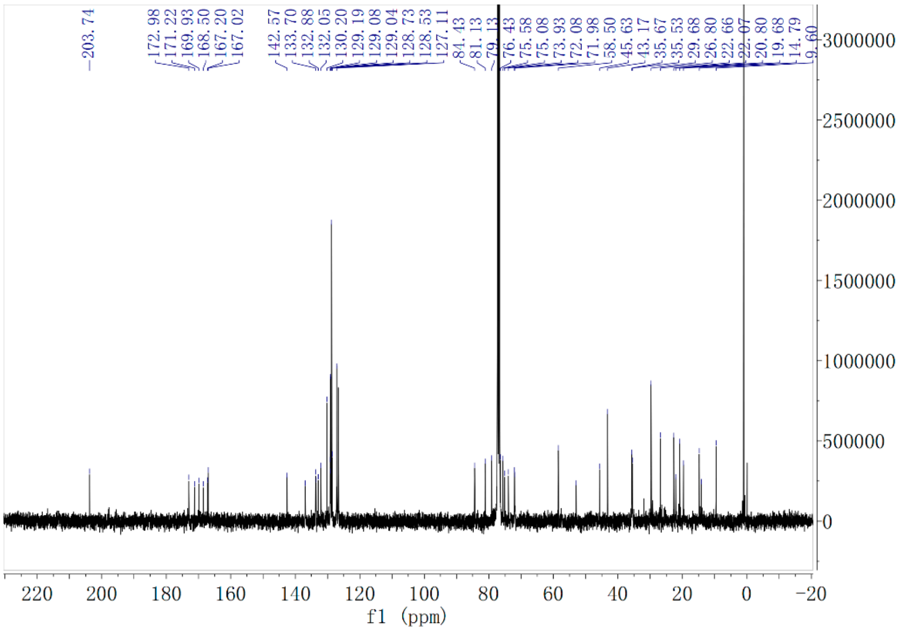

Figure S59. ^{13}C-NMR spectra of compound 17b

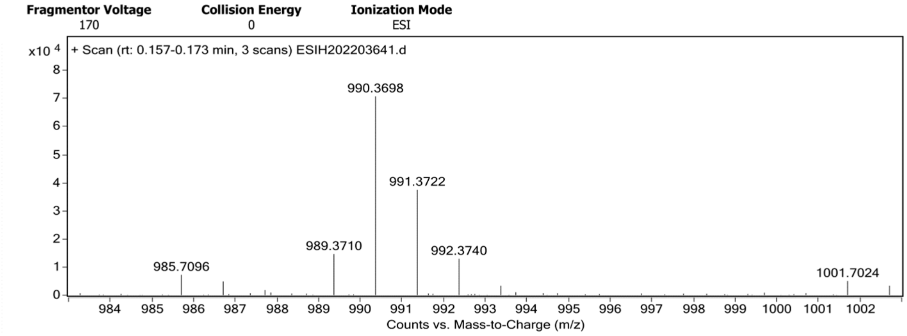

Formula Calculator Results

m/z	Calc m/z	Diff (mDa)	Diff (ppm)	Ion Formula	Ion
990.3698	990.369	-0.82	-0.82	C51 H58 [11B] N Na O17	(M+Na)+

Figure S60. HR-MS spectra of compound 17b

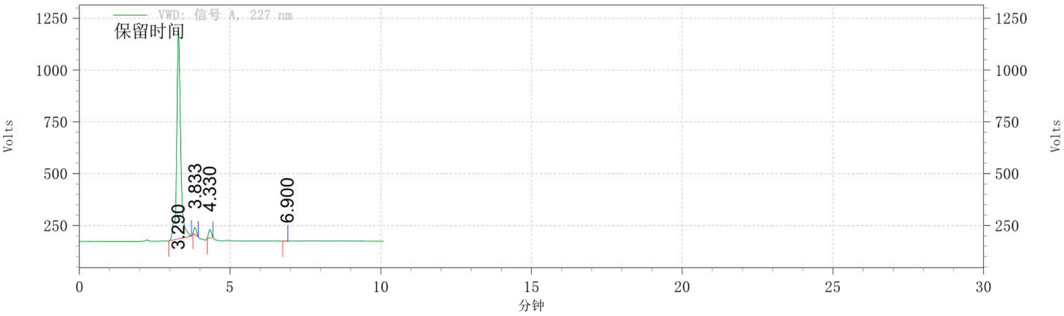

VWD: 信号 A,
227 nm 结果

保留时间	面积	面积百分比	峰高	峰高百分比
3.290	152199411	95.16	16818732	93.10
3.833	3396612	2.12	583200	3.23
4.330	4342471	2.72	662787	3.67
6.900	4388	0.00	310	0.00

Figure S61. HPLC chromatograms of compound 17b

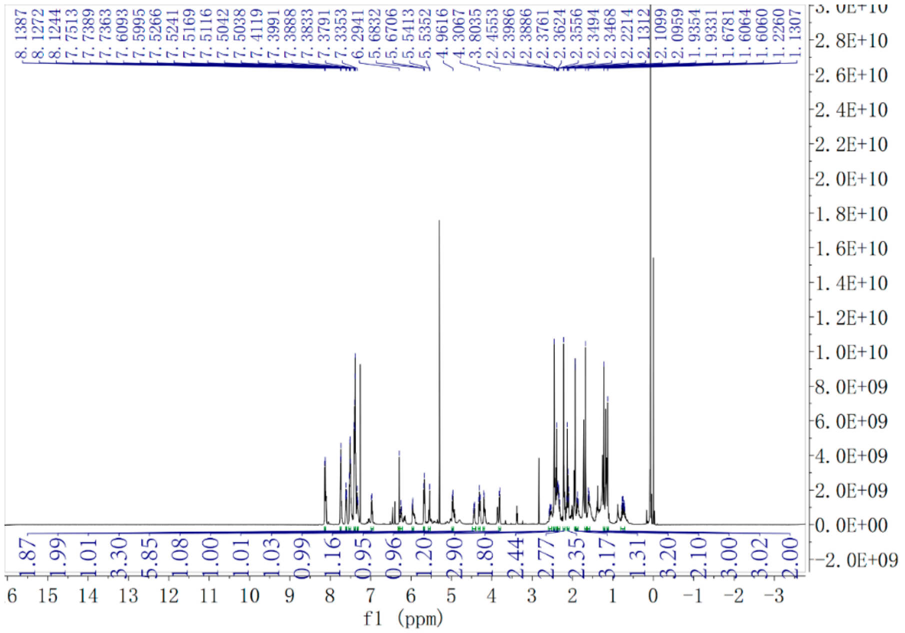

Figure S62. ^{1}H-NMR spectra of compound 17c

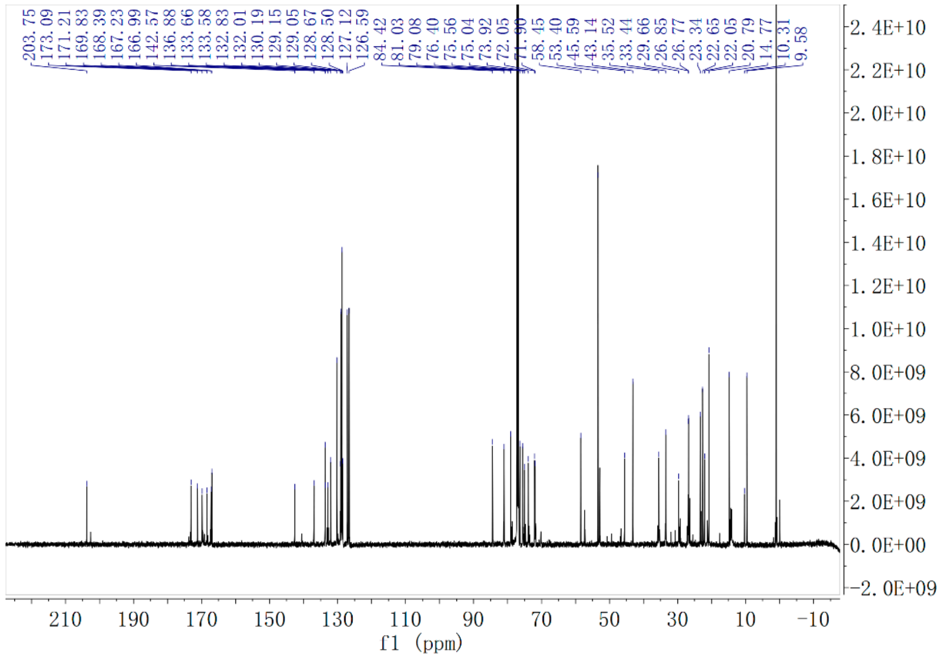

Figure S63. ^{13}C -NMR spectra of compound 17c

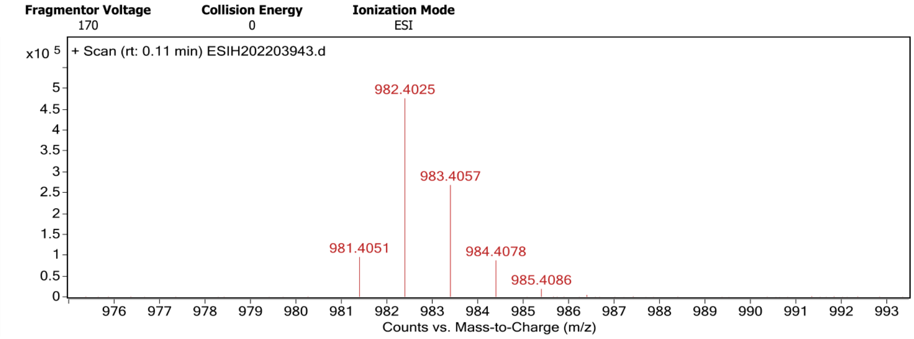

Formula Calculator Results

m/z	Calc m/z	Diff (mDa)	Diff (ppm)	Ion Formula	Ion
982.4025	982.4027	0.22	0.22	C52 H61 [11B] N O17	(M+H)+

Figure S64. HR-MS spectra of compound 17c

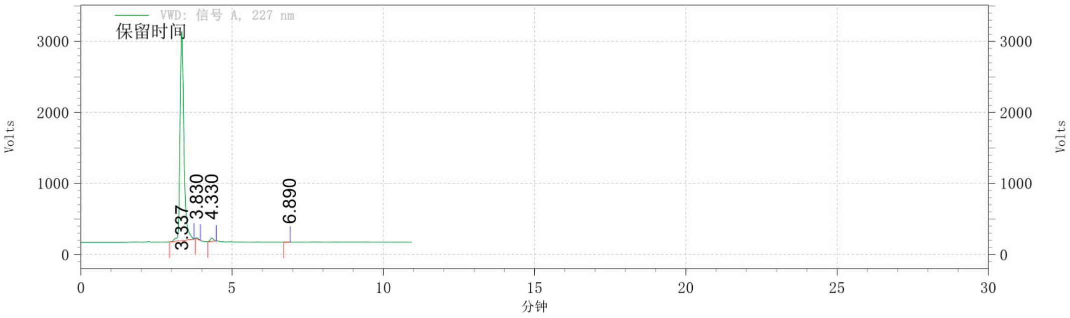

VWD: 信号 A,
227 nm 结果

保留时间	面积	面积百分比	峰高	峰高百分比
3.337	439891073	98.30	49421102	97.85
3.830	1604178	0.36	282477	0.56
4.330	5972852	1.33	800867	1.59
6.890	10452	0.00	869	0.00

Figure S65. HPLC chromatograms of compound 17c

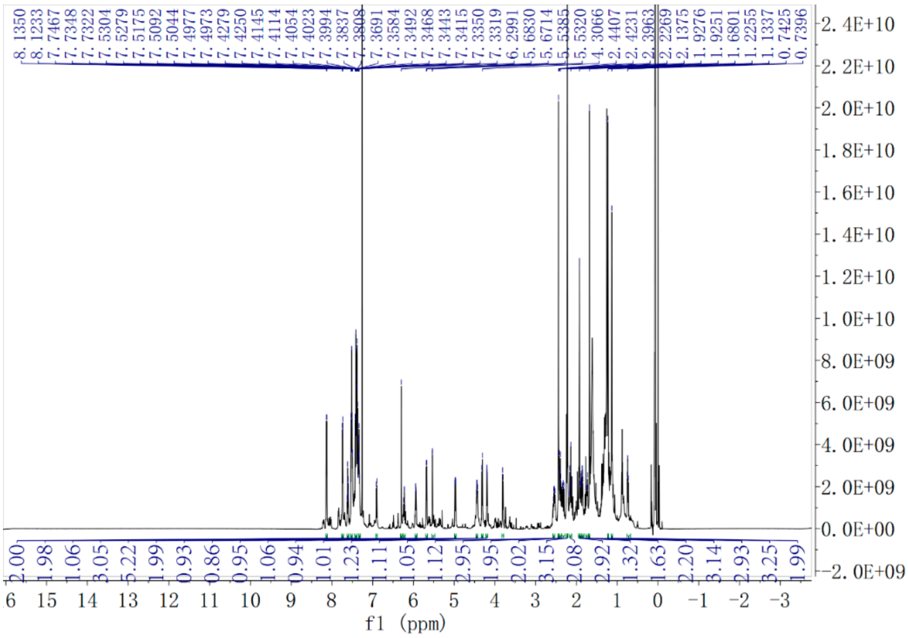

Figure S66. ^{1}H-NMR spectra of compound 17d

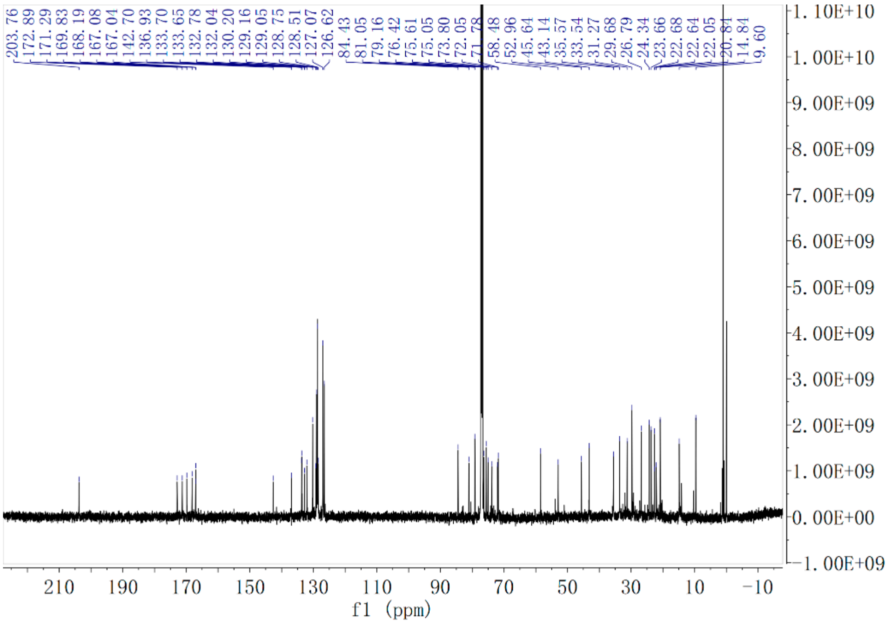

Figure S67. ^{13}C-NMR spectra of compound 17d

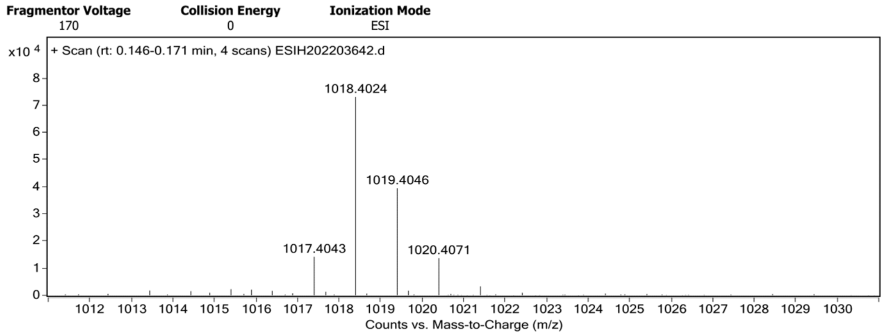

Formula Calculator Results

m/z	Calc m/z	Diff (mDa)	Diff (ppm)	Ion Formula	Ion
1018.4024	1018.4003	-2.15	-2.11	C53 H62 [11B] N Na O17	(M+Na)+

Figure S68. HR-MS spectra of compound 17d

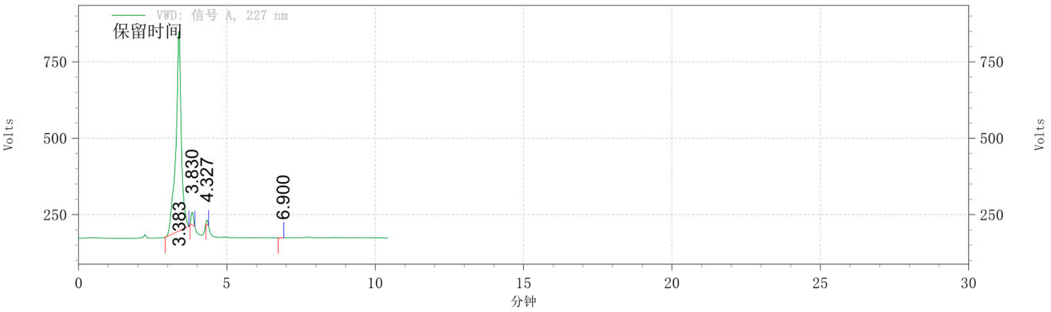

VWD: 信号 A,
227 nm 结果

保留时间	面积	面积百分比	峰高	峰高百分比
3.383	139634483	96.66	10968143	91.97
3.830	3861101	2.67	700425	5.87
4.327	960217	0.66	257259	2.16
6.900	8341	0.01	473	0.00

Figure S69. HPLC chromatograms of compound 17d

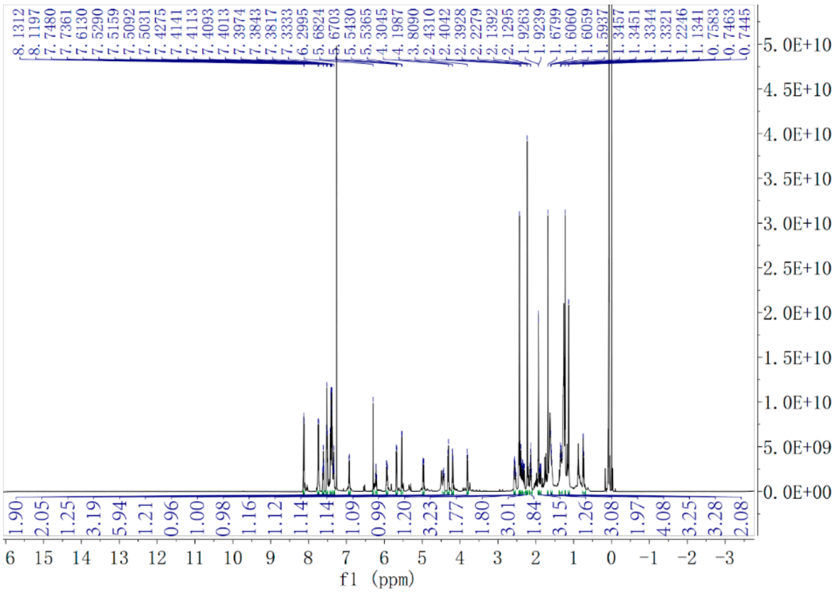

Figure S70. ^{1}H-NMR spectra of compound 17e

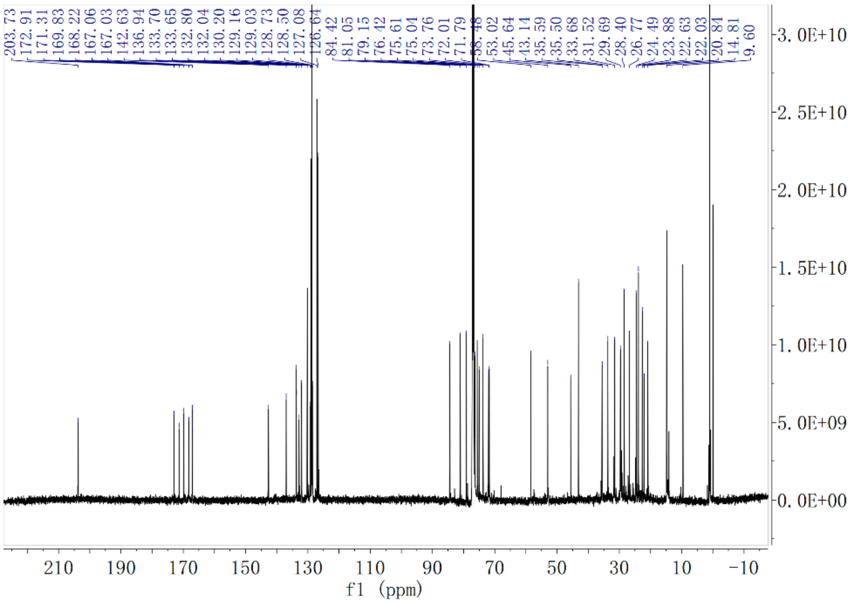

Figure S71. ^{13}C-NMR spectra of compound 17e

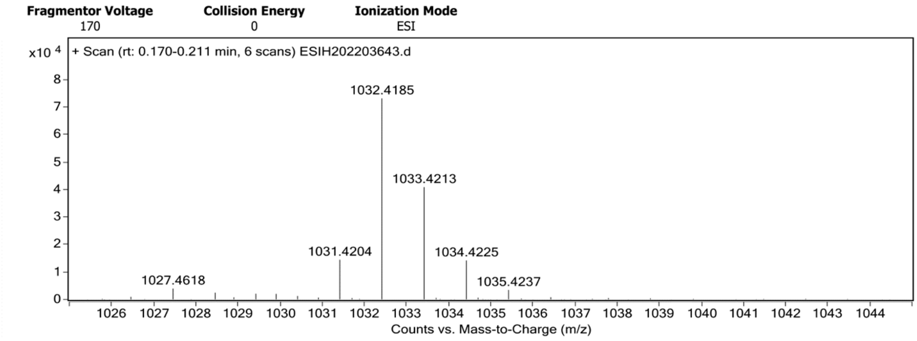

Formula Calculator Results

m/z	Calc m/z	Diff (mDa)	Diff (ppm)	Ion Formula	Ion
1032.4185	1032.416	-2.52	-2.44	C54 H64 [11B] N Na O17	(M+Na)+

Figure S72. HR-MS spectra of compound 17e

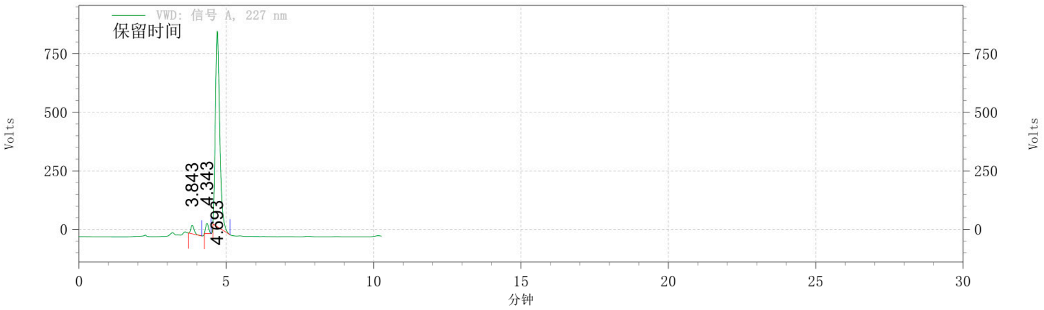

VWD: 信号 A,
227 nm 结果

保留时间	面积	面积百分比	峰高	峰高百分比
3.843	4811220	3.14	620811	4.04
4.343	5062974	3.30	732665	4.77
4.693	143362215	93.56	14006839	91.19

Figure S73. HPLC chromatograms of compound 17e

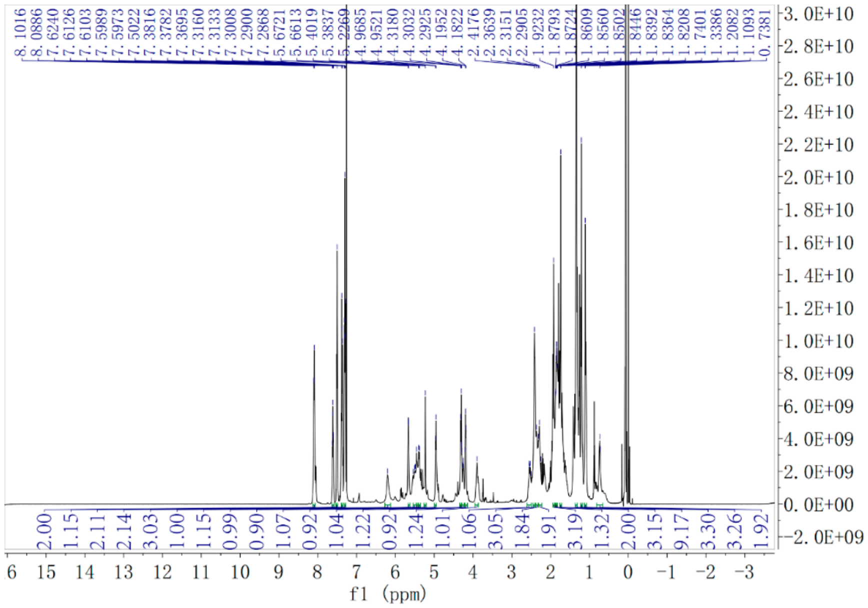

Figure S74. ^{1}H-NMR spectra of compound 17f

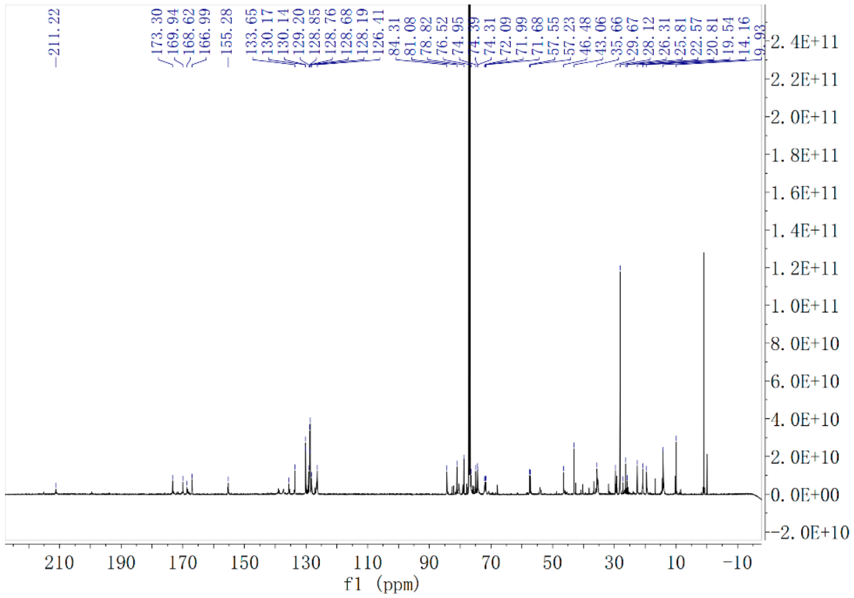

Figure S75. ^{13}C-NMR spectra of compound 17f

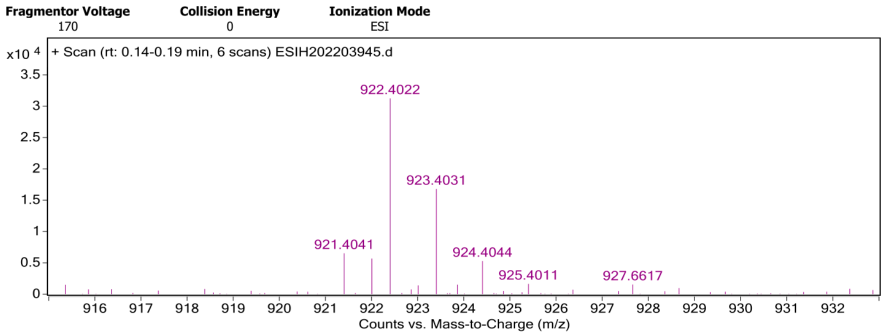

Formula Calculator Results

m/z	Calc m/z	Diff (mDa)	Diff (ppm)	Ion Formula	Ion
922.4022	922.4027	0.47	0.51	C47 H61 [11B] N O17	(M+H)+

Figure S76. HR-MS spectra of compound 17f

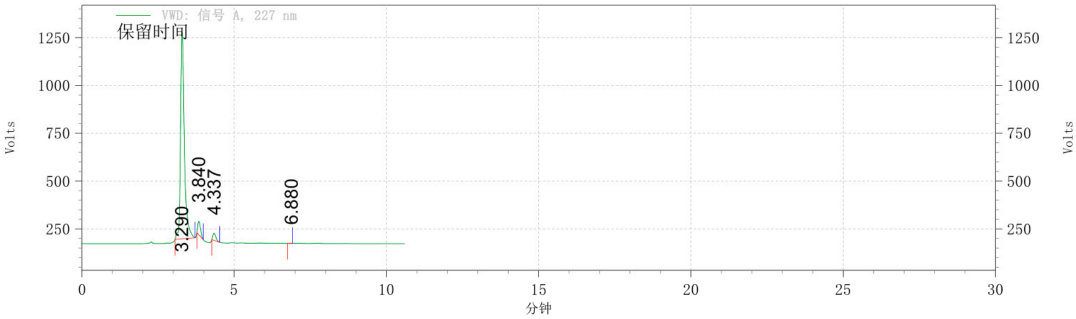

VWD: 信号 A,
227 nm 结果

保留时间	面积	面积百分比	峰高	峰高百分比
3.290	158533090	93.09	18174441	90.91
3.840	7386502	4.34	1187669	5.94
4.337	4380595	2.57	628962	3.15
6.880	4714	0.00	750	0.00

Figure S77. HPLC chromatograms of compound 17f

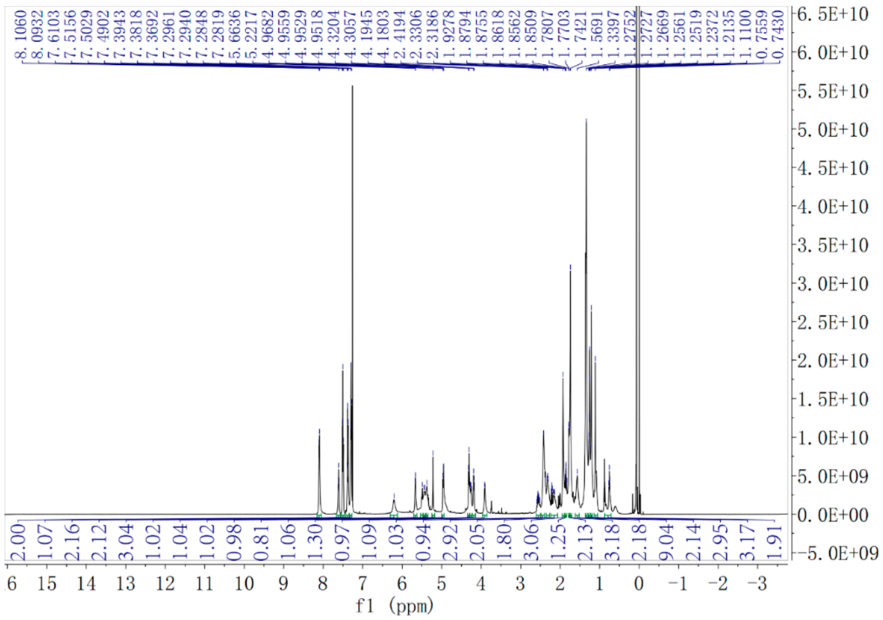

Figure S78. ^{1}H-NMR spectra of compound 17g

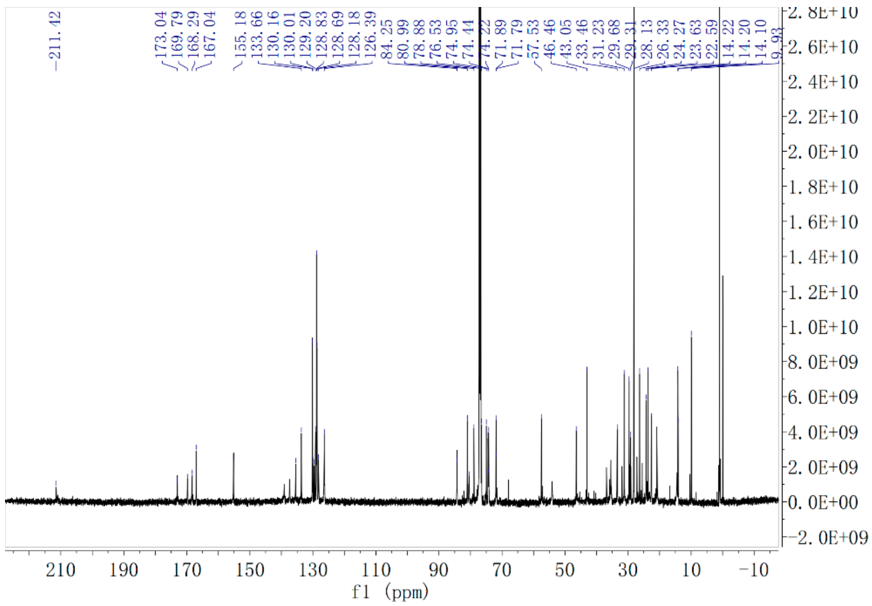

Figure S79. ^{13}C-NMR spectra of compound 17g

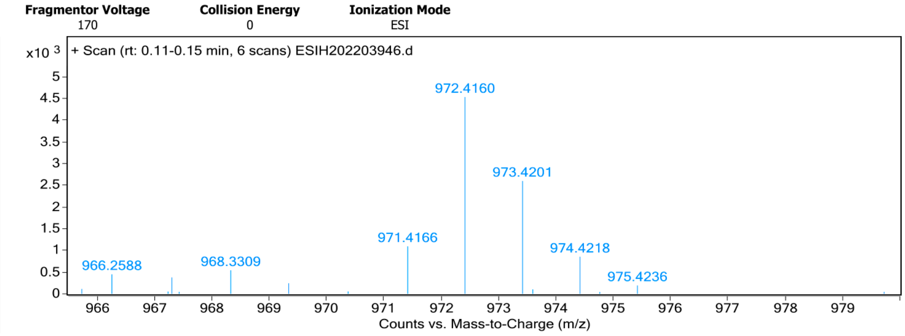

Formula Calculator Results

m/z	Calc m/z	Diff (mDa)	Diff (ppm)	Ion Formula	Ion
972.416	972.416	-0.05	-0.05	C49 H64 [11B] N Na O17	(M+Na)+

Figure S80. HR-MS spectra of compound 17g

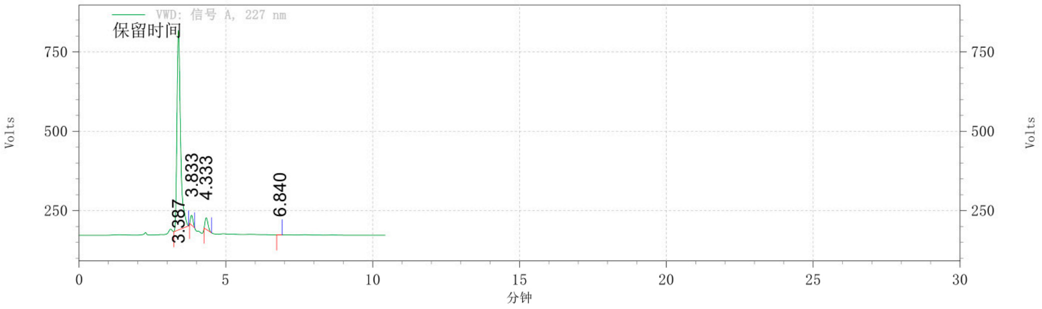

VWD: 信号 A,
227 nm 结果

保留时间	面积	面积百分比	峰高	峰高百分比
3.387	89930330	92.56	10538390	90.23
3.833	3033576	3.12	522508	4.47
4.333	4190658	4.31	616941	5.28
6.840	6514	0.01	1055	0.01

Figure S81. HPLC chromatograms of compound 17g
